# Supplementary material for: Women’s knowledge and its associated factors regarding optimum utilisation of antenatal care in rural Ghana: A cross-sectional study
Source: PLoS One. 2020 Jul 9;15(7):e0234575. doi: 10.1371/journal.pone.0234575 (PMC7347137; doi:10.1371/journal.pone.0234575)
Supplement: Attachment — Submitted filename: PONE-D-20-05929_reviewer paper for review AA review.pdf [file pone.0234575.s002.pdf]

PLOS ONE

Women's knowledge of and factors associated with the utilisation of antenatal care in rural Ghana: analysis of a community-based cross-sectional study

--Manuscript Draft--

**Manuscript Number:**	PONE-D-20-05929
**Article Type:**	Research Article
**Full Title:**	Women's knowledge of and factors associated with the utilisation of antenatal care in rural Ghana: analysis of a community-based cross-sectional study
**Short Title:**	Women's knowledge of and factors associated with the utilisation of antenatal care in rural Ghana.
**Corresponding Author:**	Agani Afaya<br>University of Health and Allied Sciences<br>Ho, GHANA
**Keywords:**	antenatal care utilization
**Abstract:**	Introduction<br><br>Improving maternal health is a global public health challenge especially sub-Saharan Africa. Utilizing antenatal care services is known to improve maternal health outcomes. Maternal morbidity and mortality rates in Ghana remains unacceptably high, particularly in rural settings. This study sought to assess women's knowledge and factors that influence the utilization of Antenatal care (ANC) services for at least four times or more in rural Ghana.<br><br>Methods<br><br>This is a cross-sectional analytical study conducted between October 2018 to January 2019. 322 women who gave birth and had attended the postnatal clinic for care were selected. Consecutive sampling was employed in recruiting participants. The associations between dependent (ANC service utilization and knowledge of ANC) and independent (socio-demographic characteristics) variables were examined using logistic regression at 95% confidence interval in STATA version 14.0.<br><br>Results<br><br>Of the 322 participants, 69.0% reported utilizing ANC services at least four times. Determinants of women attending ANC for four or more times was significantly associated with women's age [OR=4.36 (95%CI: 2.16-8.80), p<0.001], educational level [OR=10.18 (95%CI: 3.86-26.87), p<0.001], Husbands educational level [OR=8.09 (95%CI: 3.42 – 19.14), p<0.001], Insured with NHIS [OR=3.42 (95%CI: 1.72-6.82), p<0.001]. Not married [OR=0.65 (0.39 - 1.09), p=0.011] and divorced [OR=0.33 (95%CI: 0.13 – 0.83), p=0.019] were negatively associated with attending ANC services four or more times. Majority (79.2%) of the participants had a good level of knowledge regarding antenatal care.<br><br>Conclusion<br><br>Although the majority of the women had good knowledge of ANC services, a significant number of them do not complete the recommended number of ANC visits for at least four times during a normal pregnancy. The creation of awareness on adequate ANC attendance in rural settings is key in achieving high antenatal attendance and this should be championed by frontline healthcare professionals' especially nurses and midwives working in rural communities.
**Order of Authors:**	Agani Afaya
	Thomas Bavo Azongo
	Veronica Millicent Dzomeku

Powered by Editorial Manager® and ProduXion Manager® from Aries Systems Corporation

	Hyeonkyeong Lee
	Richard Adongo Afaya
	Solomon Mohammed Salia
	Peter Adatara
	Robert Kaba
	Martin Amogre Ayanoore
	Confidence alorse Atakro
	David Adadem
	Emmanuel Opoku Asiedu
	Paul Amuna
**Additional Information:**	
**Question**	**Response**
**Financial Disclosure**<br><br>Enter a financial disclosure statement that describes the sources of funding for the work included in this submission. Review the submission guidelines for detailed requirements. View published research articles from *PLOS ONE* for specific examples.<br><br>This statement is required for submission and **will appear in the published article** if the submission is accepted. Please make sure it is accurate.<br><br>**Unfunded studies**<br>Enter: *The author(s) received no specific funding for this work.*<br><br>**Funded studies**<br>Enter a statement with the following details:<br>• Initials of the authors who received each award<br>• Grant numbers awarded to each author<br>• The full name of each funder<br>• URL of each funder website<br>• Did the sponsors or funders play any role in the study design, data collection and analysis, decision to publish, or preparation of the manuscript?<br>• **NO** - Include this sentence at the end of your statement: *The funders had no role in study design, data collection and analysis, decision to publish, or preparation of the manuscript.*<br>• **YES** - Specify the role(s) played.	The author(s) received no specific funding for this work.

* typeset	
**Competing Interests**<br><br>Use the instructions below to enter a competing interest statement for this submission. On behalf of all authors, disclose any competing interests that could be perceived to bias this work—acknowledging all financial support and any other relevant financial or non-financial competing interests.<br><br>This statement **will appear in the published article** if the submission is accepted. Please make sure it is accurate. View published research articles from *PLOS ONE* for specific examples.<br><br>**NO authors have competing interests**<br><br>Enter: *The authors have declared that no competing interests exist.*<br><br>**Authors with competing interests**<br><br>Enter competing interest details beginning with this statement:<br><br>*I have read the journal's policy and the authors of this manuscript have the following competing interests: [insert competing interests here]*<br><br>* typeset	The authors have declared that no competing interests exist
**Ethics Statement**<br><br>Enter an ethics statement for this submission. This statement is required if the study involved:<br><br>• Human participants<br>• Human specimens or tissue<br>• Vertebrate animals or cephalopods<br>• Vertebrate embryos or tissues<br>• Field research<br><br>Write "N/A" if the submission does not require an ethics statement.	Ethical clearance was obtained from the University of Health and Allied Sciences Research Ethical Committee

General guidance is provided below. Consult the submission guidelines for detailed instructions. **Make sure that all information entered here is included in the Methods section of the manuscript.**<br><br>**Format for specific study types**<br><br>**Human Subject Research (involving human participants and/or tissue)**<br>• Give the name of the institutional review board or ethics committee that approved the study<br>• Include the approval number and/or a statement indicating approval of this research<br>• Indicate the form of consent obtained (written/oral) or the reason that consent was not obtained (e.g. the data were analyzed anonymously)<br><br>**Animal Research (involving vertebrate animals, embryos or tissues)**<br>• Provide the name of the Institutional Animal Care and Use Committee (IACUC) or other relevant ethics board that reviewed the study protocol, and indicate whether they approved this research or granted a formal waiver of ethical approval<br>• Include an approval number if one was obtained<br>• If the study involved *non-human primates*, add *additional details* about animal welfare and steps taken to ameliorate suffering<br>• If anesthesia, euthanasia, or any kind of animal sacrifice is part of the study, include briefly which substances and/or methods were applied<br><br>**Field Research**<br><br>Include the following details if this study involves the collection of plant, animal, or other materials from a natural setting:<br>• Field permit number<br>• Name of the institution or relevant body that granted permission	
**Data Availability**<br><br>Authors are required to make all data underlying the findings described fully	Yes - all data are fully available without restriction

Powered by Editorial Manager® and ProduXion Manager® from Aries Systems Corporation

available, without restriction, and from the time of publication. PLOS allows rare exceptions to address legal and ethical concerns. See the PLOS Data Policy and FAQ for detailed information.<br><br>A Data Availability Statement describing where the data can be found is required at submission. Your answers to this question constitute the Data Availability Statement and **will be published in the article**, if accepted.<br><br>**Important:** Stating 'data available on request from the author' is not sufficient. If your data are only available upon request, select 'No' for the first question and explain your exceptional situation in the text box.<br><br>Do the authors confirm that all data underlying the findings described in their manuscript are fully available without restriction?	
**Describe where the data may be found in full sentences. If you are copying our sample text, replace any instances of XXX with the appropriate details.**<br><br>• If the data are **held or will be held in a public repository**, include URLs, accession numbers or DOIs. If this information will only be available after acceptance, indicate this by ticking the box below. For example: *All XXX files are available from the XXX database (accession number(s) XXX, XXX.*).<br>• If the data are all contained **within the manuscript and/or Supporting Information files**, enter the following: *All relevant data are within the manuscript and its Supporting Information files.*<br>• If neither of these applies but you are able to provide **details of access elsewhere**, with or without limitations, please do so. For example:<br><br>*Data cannot be shared publicly because of [XXX]. Data are available from the XXX Institutional Data Access / Ethics*	Data will be available with the corresponding author based on reasonable request

Committee (contact via XXX) for researchers who meet the criteria for access to confidential data.<br><br>*The data underlying the results presented in the study are available from (include the name of the third party and contact information or URL).*<br>• This text is appropriate if the data are owned by a third party and authors do not have permission to share the data.<br><br>* typeset	
Additional data availability information:	

Powered by Editorial Manager® and ProduXion Manager® from Aries Systems Corporation

Manuscript Click here to download Manuscript ANC manuscript.docx

Title page

Manuscript Title: Women's knowledge of and factors associated with the utilization of antenatal care in rural Ghana: analysis of a community-based cross-sectional study

[1]Agani Afaya (MSc) [2]Thomas B. Azongo (PhD), [3]Veronica Millicent Dzomeku (PhD), [4]Hyeonkyeong Lee (PhD), [3]Richard Adongo Afaya (MPhil), [1]Solomon Mohammed Salia (MSc), [1]Peter Adatara (PhD), [5]Robert Kaba (PhD), [6]Martin Ayanoore (PhD), [7]Confidence alorse Atakro (MN), [1]David Adadem (BN),[1]Emmanuel Opoku Asiedu (BN), [6]Paul Amuna (MB ChB, PhD)

1. Department of Nursing, School of Nursing and Midwifery, University of Health and Allied Sciences, Ho, Ghana
2. Department of Public Health, School of Allied Health Sciences, University for Development Studies, Tamale, Ghana
3. Department of Nursing, Kwame Nkrumah University of Science and Technology, Ghana
4. College of Nursing, Yonsei University Seoul, South Korea
5. Centre for Health Policy and Implementation Research. Institute of Health Research University of Health and Allied Sciences, Ho, Ghana
6. School of Public Health, University of Health and Allied Sciences, Hohoe, Ghana
7. School of Nursing and Midwifery, Christian Service University College, Kumasi, Ghana

Correspondence

Agani Afaya (aagani@uhas.edu.gh)
Department of Nursing, School of Nursing and Midwifery, University of Health and Allied Sciences, Ho, Ghana

Abstract

Introduction: Improving maternal health is a global public health challenge especially in sub-Saharan Africa. Utilising antenatal care services is known to improve maternal health outcomes. Maternal morbidity and mortality rates in Ghana remains unacceptably high, particularly in rural settings. This study sought to assess women's knowledge and factors that influence the utilisation of Antenatal care (ANC) services for at least four times or more in rural Ghana.

Methods: This is a cross-sectional analytical study conducted between October 2018 to January 2019. Of 322 women who gave birth and had attended the postnatal clinic for care were selected. Consecutive sampling was employed in recruiting participants. The associations between dependent (ANC service utilization and knowledge of ANC) and independent (socio-demographic characteristics) variables were examined using logistic regression at 95% confidence interval in STATA version 14.0.

Results: Of the 322 participants, 69.0% reported utilising ANC services for at least four times. Determinants of women attending ANC for four or more times was significantly associated with women's age [OR=4.36 (95%CI: 2.16-8.80), p<0.001], women's educational level [OR=10.18 (95%CI: 3.86-26.87), p<0.001], Insured with NHIS [OR=3.42 (95%CI: 1.72-6.82), p<0.001]. Not married [OR=0.65 (0.39 - 1.09), p=0.011] and divorced [OR=0.33 (95%CI: 0.13 – 0.83), p=0.019] were negatively associated with attending ANC services for four or more times. Majority (79.2%) of the participants had a good level of knowledge regarding antenatal care.

Conclusion: Although the majority of the women had good knowledge of ANC services, a significant number of them do not complete the recommended number of ANC visits for at least four times during a normal pregnancy. The creation of awareness on adequate ANC attendance in rural settings is key in achieving high antenatal attendance and this should be

championed by frontline healthcare professionals' especially nurses and midwives working in rural communities.

Keywords Antenatal care utilisation, women's knowledge, factors, rural setting, Ghana

Introduction

Globally, about 303 000 women and adolescent girls die as a result of pregnancy and childbirth-related complications [1] of which an alarming 99% of these maternal deaths occur in low-resource settings. Though globally, the number of women and girls dying due to pregnancy-related complications and childbirth decreased by nearly half (50%) from 1990 to 2013, the number of deaths within the West African region remains unacceptably high with Maternal Mortality Ratio (MMR) of 679 deaths per 100,000 live births in 2015 [2-4].

According to the 2017 Ghana Maternal Health Survey (GMHS), the MMR in Ghana is 310 deaths per 100,000 live births, which remains unacceptably high. Data from the 2017 GMHS shows that nearly half of the women (50%) in rural settings of the Northern and Volta regions of Ghana deliver at home compared with the national average of 21% [5]. Delivering at home by a non-health professional may predispose the woman to some life-threatening conditions but delivering at a health facility allows the woman to receive emergency care by skilled birth health professionals when complications arise which can help save the lives of both baby and mother [5]. Antenatal care utilisation can pre-empt women the need for hospital delivery, thereby reducing possible pregnancy-related morbidities and mortalities.

Antenatal care (ANC) is the health care and education provided to pregnant women and adolescent girls by skilled health care professionals to ensure the best health conditions for the mother and the baby during pregnancy [1, 5]. Antenatal care utilisation is an important constituent of maternal health care, which reduces maternal and perinatal morbidity and

mortality both directly through identification and management of pregnancy-related complications, and indirectly through the identification of pregnant women and girls most likely to develop complications during labour and delivery, thus ensuring early referral to an appropriate health facility for further care [1]. Globally, while most women now attend at least one ANC visit (86%), only 62% attend four, with lower rates reported in sub-Saharan Africa and South Asia [6]. An analytical review of the recent WHO Global Health Observatory data repository shows that ANC coverage, between 2000 and 2017, was indirectly correlated with MMR worldwide [7, 8]. The evidence indicates that countries with poor ANC coverage are more likely to have high MMR [9-11]. For example, ANC utilisation in Australia is 94% with MMR of 6 per 100, 000, Finland has 99% ANC utilisation with MMR 3 per 100, 000 and France has 99% ANC utilisation with MMR 8 per 100000 live births. In comparison with sub-Saharan Africa, Nigeria has 49.1% ANC coverage with MMR of 917 per 100000, Cote d'Ivoire has 51.3% ANC coverage with MMR of 617 per 100,000, and Ghana has 89.3% ANC coverage with MMR 310 deaths per 100, 000 live births [7,8]. Ghana has a high ANC coverage but still has a high MMR. This poor maternal health outcome in Ghana could be due to inadequate ANC utilisation by women and possibly none facility-based delivery. This study sought to assess women's knowledge and factors that influence the utilisation of ANC services at least four times during a normal pregnancy

Conceptual framework

Taking the decision to utilise ANC services does not only depend on the individual's preference but also the factors that facilitate the accessibility of the facility (services) or the factors that impede the utilisation of ANC services [12]. WHO outlined that a woman's ability to access antenatal care services consisted of several factors including distance or time taken to a facility, availability of ANC services, social and cultural factors that can serve as

barriers to access, economic status, costs related with utilisation of services, and the quality of services provided [13].

A number of models have been developed over the last few decades to identify factors influencing healthcare service utilisation, and the most widely used is the Anderson Behavioral Health care model known as Behavioral Model of Health Services. This study was grounded on the basis of the Andersons Behavioral Model and sought to identify factors that facilitate or impede the use of healthcare and to understand discrepancies in the utilisation of care [14]. This model was first developed to determine why families utilise health care, to identify equitable access to health services, and to support in formulating policies that will promote equitable access [15, 16]. According to the model, utilisation of health services is determined by three dynamics: (1) the predisposing factors, (2) enabling factors, and (3) need factor. Predisposing factors include individual characteristics and health beliefs; enabling factors are the logistic aspect of obtaining care and need factors are the most immediate cause of healthcare utilisation such as the presence of disease condition and self-perceived functional or health status [15].

The current study sought to assess factors that facilitate or impede women from the use of ANC services; community-based factors that impede or motivate the use of ANC, facility-based factors, and adequacy of ANC visits. Despite the importance of these individual determinants affecting ANC utilisation among women, the current study also sought to assess women's knowledge of ANC services, as it is recognised that a woman's knowledge of ANC services has a significant correlation with her utilising ANC services [17] for at least four times as recommended by WHO. The study identified the following (as shown in figure 1) factors that influence ANC utilisation (either positive or negative) in the study setting among women.

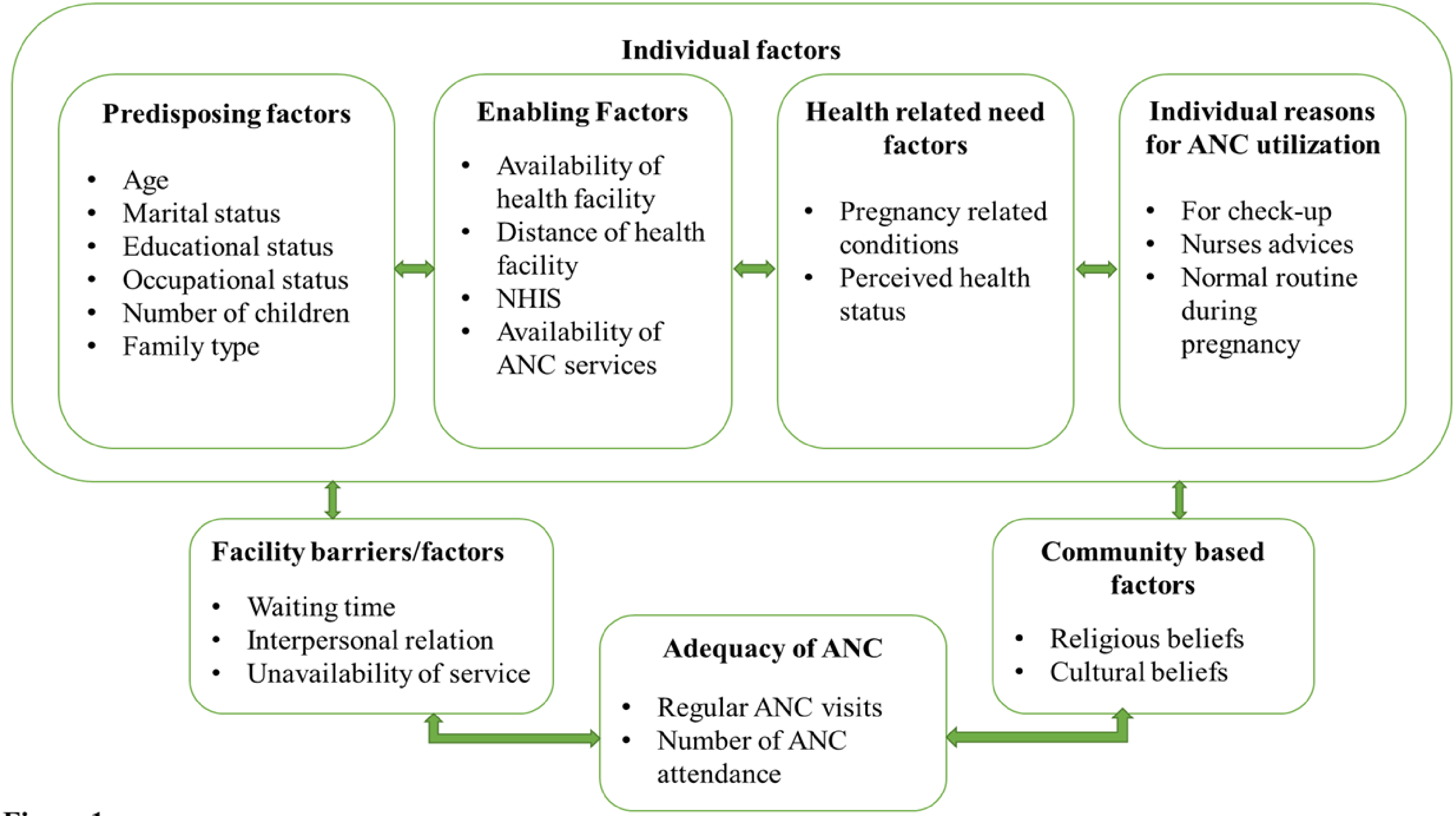

Figure 1:

- Conceptual framework for analyzing factors associated with ANC utilization among women in Northern Ghana
- Adapted from Andersen's behavioral model for health service use (Andersen, 1995)

Methods

Study Design

This was a cross-sectional analytical community-based study design conducted in Bamboi, a sub-district under the Bole district in the Savanah region of Ghana. The study employed quantitative research approaches for collecting data from the participants. The sub-district has semi-urban area but predominantly rural.

Study setting

The Bole District is one of the seven districts located in the newly created Savanah region of Ghana. The District is located at the extreme western part of the northern region of Ghana. The District is bordered to the North by Sawla / Tuna Kalba District, to the West by the Republic of Ivory Coast, to the East by West Gonja District and to the South by Wenchi and Kintampo Districts of Brong-Ahafo region. The District health services are divided into four sub-districts namely Bole, Tinga, Jama, and Bamboi. Each sub-district has an operational area served by a health facility. Bamboi is one of the sub-districts which is to the South of the Bole district. The Bamboi sub-district has one health center and three Community-Based Health Planning and Services (CHPS) serving over 26 communities within the sub-district. The facilities were categorized as (A, B, C & D). Facility A had two midwives, four Enrolled Nurses (EN) and a community health nurse (CHN), facility B had two CHNs, facility C had a midwife and one CHN and facility D had a male practicing midwifery. These health professionals provide maternal and child health services for mothers attending any of these facilities.

Study Population

The target study population were all reproductive-aged women in the Bambi sub-district who attended the health facilities within the sub-district for postnatal care.

Inclusion and Exclusion Criteria

Women of reproductive age, who had their ANC services, gave birth and received postnatal care services in the four health facilities in Bamboi sub-district and voluntarily consented to take part in this study were included. However, women who had their ANC and postnatal services outside of the Bamboi sub-district were excluded from the study. Women who did not have their ANC in any of the facilities in the Bamboi sub-district but had their postnatal services there were also excluded. Furthermore, women who met the inclusion criteria but did not consent willingly to take part in the study were as well excluded from the study.

Sampling Method and Sample Size Determination

Consecutive sampling was employed in recruiting participants. The average monthly attendance for the four facilities providing postnatal care services was estimated to be about 450. The total population for the period of four months was 1800. The sample size for the study was determined by using the sample size calculation by Yamane [18]. The minimum required sample size was 310 and a 5% non-response rate was added to get a sample size of 340. Finally, 322 questionnaires were considered valid to be included for analysis.

Outcome variables

During data analysis, the outcome variables were the utilisation of ANC services which was classified as adequate versus inadequate. The study assessed the overall adequacy of ANC visits by reviewing the mothers' ANC cards to determine the frequency of visits during the immediate past pregnancy. Antenatal care was classified as adequate if the woman had at least four or more antenatal visits during normal pregnancy and less than four visits were defined as inadequate. The knowledge of mothers on ANC services was classified as good knowledge and poor knowledge as one of the outcome variables. A mother was

knowledgeable if she was able to answer the questions correctly in figure 2 and scored 50% and above, a mother who scored below 50% was considered to have poor knowledge.

Explanatory variables

The explanatory variables of the study consisted of a number of covariates. These covariates were selected based on review of related literature and the modified version of the Anderson Healthcare Utilization model. *Socio-demographic characteristics;* Participants' age was categorised into <20yrs, 21-30yrs and 31-46 years. Marital status was categorised into not married, married and divorced. Education level of participants were categorised as none, basic, secondary and tertiary. Occupation of participants was classified into farmer, trader, civil servant, and others. The religious status was categorized under Christianity and Islam. Mo, Gonja, Ewe, Akan, and Dagaaba were the ethnic groups of participants. The family type was the nuclear and extended family. The number of children was dichotomised into 1-2, 3-4 and 5+ children. Participants were either insured or not insured with the National Health Insurance Scheme (NHIS). Other variables of interest were distance to health facility (far or near), person who provides healthcare, person who makes healthcare decisions and means of transportation to the facility.

Data collection procedure

Data collection lasted for four months from October 2018 to January 2019. A self-administered semi-structured and pretested questionnaire was used to collect data from mothers seeking postnatal care. Four trained research assistants (nurses) were used to gather data from mothers in the four health facilities. The research assistants were trained for 2days and were supervised for a week by the principal investigator during data collection. Challenges at the initial stages were quickly identified and corrected for the smooth running of data collection. The four facilities were visited every week during the postnatal days for

questionnaire administration. Mothers were approached during postnatal care and assisted to answer the questionnaire after which the completed questionnaires were cross-checked for completeness. Immediate corrective measures were taken with participants to ensure validity and to prevent missing information.

Data Analysis

Data were entered into SPSS software, cleaned and validated to ensure quality before analysis. The cleaned data were exported to STATA version 14.0 for analysis. Simple frequencies and percentages were used for categorical variables. Chi-square test of association was used to determine the relationship between dependent variables (ANC service utilization and knowledge of ANC) and the independent variables (socio-demographic characteristics). Utilisation of ANC was measured using the number of antennal visits. Those who sought ANC services less than four times were classified as having poor ANC service utilisation (inadequate) and those who had ANC visits of 4 or more were considered as having good (adequate) ANC service utilisation. The overall level of knowledge on antenatal care was measured by scoring responses that measured participants' knowledge. A score of '1' was assigned if the participant had knowledge and '0' if they had no knowledge. Total score and mean scores were computed and a score below the mean was considered poor knowledge whiles a mean score or above was considered to be a good knowledge. Bivariate logistic regression analysis, computing odds ratio, was used to determine the strength of the association between dependent variables (ANC service utilisation and knowledge of ANC) and socio-demographic characteristics. Statistical significance was considered based on a p-value of less than 0.05 at a 95% confidence level. For the purpose of the logistic regression, ANC service utilisation was coded as 0 for poor utilisation (<4 ANC visits) and 1 for good utilisation (4+

ANC visits). Overall level of knowledge was coded zero for poor level of knowledge and 1 for good level of knowledge.

Validity and reliability of the study instrument

The study instrument was developed after reviewing relevant related literature and also adopting Anderson health care behavioral model. Preceding data collection, the validity and reliability of the study instrument were assessed through various stages. To assess the content validity, a group of researchers and clinicians were made to review and refine the items in the questionnaire. This group comprised of two senior community midwives and two researchers in the field of maternal and child health. Further, the Pearson correlation coefficient was used to determine the test-retest reliability of the study with a sample of 20 participants. The instrument was administered to the same participants' weeks late. The test-retest correlation coefficient for the instrument was 0.73 ($p < 0.001$), which is indicative of acceptable stability over time.

Ethical consideration

Ethics approval for the study was obtained from the University of Health and Allied Sciences Research Ethical Committee. Permission to conduct the study was sought from the various facilities before the commencement of data collection. Both verbal and written consent was obtained from participants after agreeing to participate in the study. Participants were assured of anonymity, confidentially and privacy. Participants were made aware of the right to withdraw from the study at any particular point in time if they wished to do so.

Questions asked on ANC knowledge

1. ANC can prevent complications in pregnancy?
2. Pregnant women may have problems without ANC?
3. Regular ANC medications can promote optimal growth of unborn child?
4. Health facility delivery is safer and better than home delivery?
5. Do you know the recommended place for delivery?
6. Do you know the recommended frequency and timing of ANC visits?
7. ANC is recommended regardless of complications?
8. Do you know items to prepare for before delivery?
9. Do have knowledge of family planning?
10. Do have knowledge of malaria prevention?

Figure 2: Key items used to assess women's knowledge of ANC services

Results

Participants' socio-demographic characteristics

The majority, 177 (55.0%) of participants were aged 21-30 years while about half, 167 (51.9%) were not married. Most of the women, 111 (34.5%) had basic and secondary education and the majority 188 (58.4%) engaged in trading. About 144 (44.7%) of them belonged to the Dagaaba ethnic group. The majority 288 (89.4%) had the nuclear family type and over two-thirds had 1-2 children. Majority 277 (86.1%) trekked on foot to access healthcare; 102 (31.7%) had health facilities that were far from their residence. Most, 210 (65.2%) made their own decision regarding their healthcare needs (Table 1).

Table 1: Participants' socio demographic characteristics

Variable	Frequency (N=322)	Percentage (%)
**Age**		
≤ 20	44	13.6
21-30	177	55.0
31-40	101	31.4
**Marital Status**		
Married	133	41.3
Not married	167	51.9
Divorced	22	6.8
**Educational status**		
None	33	10.2
Basic	111	34.5
Secondary	111	34.5
Tertiary	67	6.8
**Employment**		
Employed	122	37.9
Unemployed	189	58.7
Student	11	3.4
**Occupation**		
Farmer	56	17.4
Trader	188	58.4
Civil servant	56	17.4
Others	22	6.8
**Religion**		
Christianity	255	79.2
Islam	67	20.8
**Ethnicity**		
Mo	100	31.1
Gonja	33	10.3
Ewe	11	3.4
Akan	34	10.5
Dagaaba	144	44.7
**Family type**		
Nuclear	288	89.4

Extended	34	10.6
**Number of children**		
1-2 children	245	76.1
3-4 children	55	17.1
5+ children	22	6.8
**Number of ANC visits**		
<4	100	31.0
4+	222	69.0
**Health insurance**		
Insured	245	76.1
Not insured	77	23.9
**Distance to health facility**		
Short	220	68.3
Far	102	31.7
**Transportation to ANC**		
Motorcycle	23	7.1
Foot	277	86.1
Others	22	6.8
**Person who provides for healthcare**		
Self	90	27.9
Partner	166	51.6
Parents	66	20.5
**Person who makes healthcare decision**		
Self	210	65.2
Partner	101	31.4
Parents	11	3.4

Factors associated with the utilization of antenatal care services

Logistic regression analysis showed that women who were aged 21-30 years were 4 times more likely to utilize ANC services four or more times compared to those aged 20 years and below [OR=4.36 (95%CI: 2.16-8.80), p<0.001] and those who were divorced were 67% times less likely to utilise ANC services at least four times compared to those who were married [OR=0.33 (95%CI: 0.13 – 0.83), p=0.019]. Women who had basic, secondary and tertiary education were 8.1, 2.9 and 10.2 times more likely to utilise ANC services compared to those who had no education [OR=8.09 (95%CI: 3.42 – 19.14), p<0.001; OR=2.93 (95%CI: 1.29 – 6.64), p=0.010; OR=10.18 (95%CI: 3.86-26.87), p<0.001] respectively. Participants who were insured with the NHIS were 3 times more likely to utilize the ANC compared to those who were not-insured [OR=3.42 (95%CI: 1.72-6.82), p<0.001]. Those who assessed care at CHPS were 90% times less likely to utilize ANC compared to those who assessed care at the clinics [OR=0.10 (95%CI:0.05 – 0.22), p<0.001], those with far distance to the health facility were 80% times less likely to utilize ANC compared to those with short distance [OR=0.20 (95%CI: 0.12-0.34), p<0.001]. Participants whose partners provided for their healthcare were twice more likely to utilize ANC services [OR=2.44 (95%CI:1.38-4.33), p=0.002]. The factors that are associated with the utilisation ANC services are shown in table 2.

Table 2: Regression analysis results for ANC attendance

Variable	Usage of ANC Services		χ2	p-value	COR [95% CI], p-value
	<4=**100 n (%)**	**4+=222 n (%)**			
**Age**					
≤20	22 (22.0)	22 (9.9)			
21-30	33 (33.0)	144 (64.9)			4.36 [2.16 - 8.80], <0.001
31-46	45 (45.0)	56 (25.2)	28.71	**<0.001**	1.24 [0.61 - 2.53], 0.546
**Marital Status**					
Married	33 (33.0)	100 (45.0)			
Not married	56 (56.0)	111 (50.0)			0.65 [0.39 - 1.09], 0.011
Divorced	11 (11.0)	11 (5.0)	6.59	**<0.001**	0.33 [0.13 - 0.83], 0.019
**Educational status**					
None	22 (22.0)	11 (5.0)			
Basic	22 (22.0)	89 (40.1)			8.09 [3.42 - 19.14], <0.001
Secondary	45 (45.0)	66 (29.7)			2.93 [1.29 - 6.64], 0.010
Tertiary	11 (11.0)	56 (25.2)	37.46	**<0.001**	10.18 [3.86-26.87], <0.001
**Employment**					
Employed	33 (33.0)	89 (40.1)			
Unemployed	67 (67.0)	122 (55.0)			0.67 [0.41 - 1.11], 0.122
Student	0 (0.00)	11 (4.9)	7.57	0.023	1 (empty)
**Occupation**					
Farmer	33 (33.0)	23 (10.4)			
Trader	45 (45.0)	143 (64.4)			4.55 [2.43 - 8.55], <0.001
Civil servant	11 (11.0)	45 (20.3)			5.87 [2.51 - 13.69], <0.001
Others	11 (11.0)	11 (4.9)	31.86	0.064	1.43 [0.53 - 3.86], 0.475
**Religion**					
Christianity	44 (44.0)	211 (95.1)			
Islam	56 (56.0)	11 (4.9)	109.02	0.079	0.04 [0.02 - 0.08], <0.001
**Ethnicity**					
Mo	22 (22.0)	78 (35.2)			
Gonja	22 (22.0)	11 (4.9)			0.14 [0.06 - 0.33], <0.001
Ewe	0 (0.00)	11 (4.9)			1 (empty)
Akan	12 (12.0)	22 (9.9)			0.52 [0.22 - 1.21], 0.127

Dagaaba	44 (44.0)	100 (45.1)	28.63	**<0.001**	0.64 [0.35 - 1.16], 0.140
**Family type**					
Nuclear	88 (88.0)	200 (90.1)			
Extended	12 (12.0)	22 (9.9)	0.32	0.572	0.81 [0.38 - 1.70], 0.573
**Health insurance**					
Not Insured	11 (11.0)	66 (29.7)			
Insured	89 (89.0)	156 (70.3)	13.29	**<0.001**	3.42 [1.72 - 6.82], <0.001
**Distance to health facility**					
Short	44 (44.0)	176 (79.3)			
Far	56 (56.0)	46 (20.7)	39.65	**<0.001**	0.20 [0.12 - 0.34], <0.001
**Transportation to ANC**					
Motorcycle	11 (11.0)	12 (5.4)			
Foot	67 (67.0)	210 (94.6)			2.87 [1.21 - 6.81], 0.017
Others	22 (22.0)	0 (0.00)	57.96	0.081	1 (empty)
**Person who provides for healthcare**					
Self	34 (34.0)	56 (25.2)			
Partner	33 (33.0)	133 (59.9)			2.44 [1.38 - 4.33], 0.002
Parents	33 (33.0)	33 (14.9)	22.65	**<0.001**	0.60 [0.32 - 1.16], 0.129
**Person who makes healthcare decision**					
Self	55 (55.0)	155 (69.8)			
Partner	34 (34.0)	67 (30.2)			0.69 [0.42 - 1.17], 0.173
Parents	11 (11.0)	0 (0.00)	27.06	0.051	1 (empty)

Empty: perfect success or failure predicted by the model.

Participants Knowledge of Antenatal Care Services

Figure 3 shows that 79.2% of the participants had good level of knowledge regarding antenatal care.

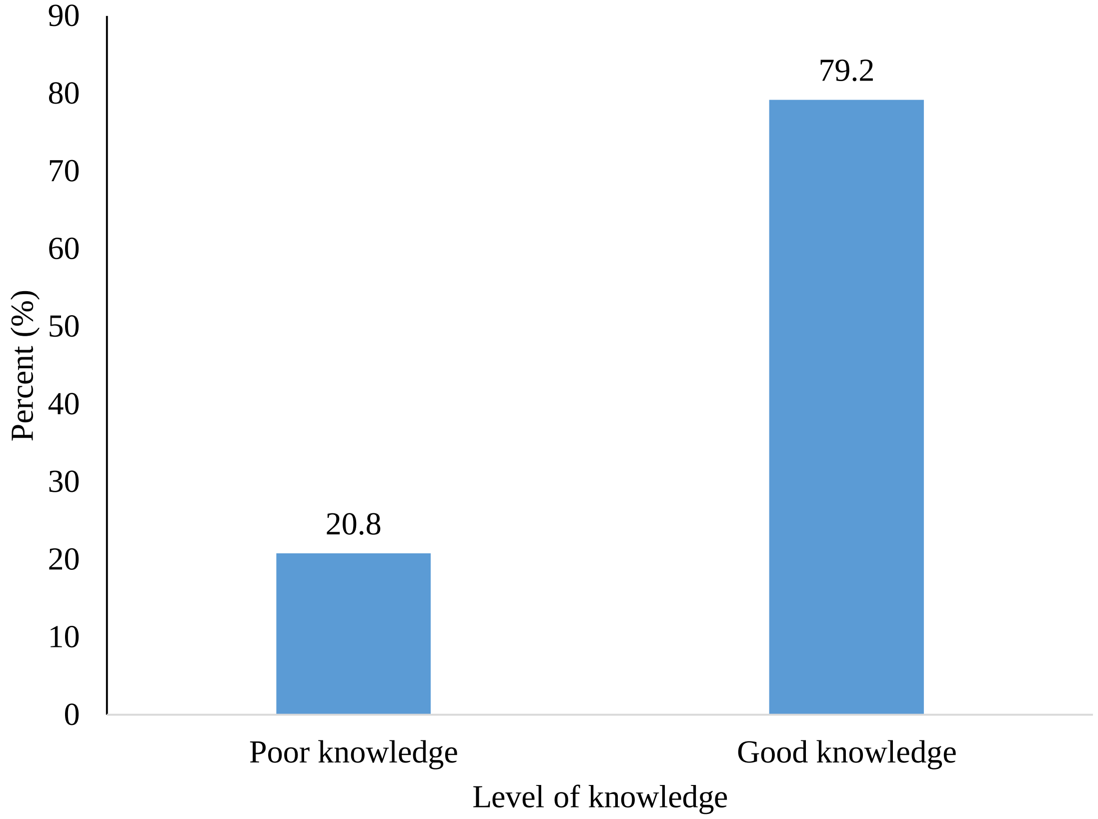

Figure 3: Overall level of knowledge regarding ANC

Association between Socio-demographics characteristics of participants and knowledge of antenatal care

Women aged 21-30 years and those above 30 years were 95% and 98% less likely to have good knowledge of ANC compared to those aged less than 20 years [OR=0.05, (95%CI: 0.00 – 0.81), p=0.03] and [OR=0.02, (95%CI: 0.00 – 0.36), p=0.008] respectively. Unmarried and divorced women were 75% and 91% respectively less likely to have good knowledge regarding ANC [OR=0.25, (95%CI: 0.13 – 0.51), p<0.001; OR=0.09, (95%CI: 0.03 – 0.26), p<0.001]. Women who attained basic school education were 4.47 times more likely to have good knowledge of ANC compared to those with no education [OR=4.47, (95%CI: 1.75 – 11.39), p=0.002].

Islamic respondents were 85% less likely to have good knowledge compared to Christians [OR=0.15, (95%CI: 0.08 – 0.27), $p<0.001$]. Those of the extended family were 99% less likely to have good knowledge compared to those who were of the nuclear family [OR=0.01, (95%CI: 0.00 – 0.03), $p< 0.001$] (see table 3).

Table 3: Factors associated with knowledge of antenatal care

Variable	Knowledge of ANC Services		χ2	p-value	OR [95% CI], p-value
	**Poor n=67 (%)**	**Good n=255 (%)**			
**Age (years)**					
≤ 20	0 (0.00)	44 (17.2)			
21-30	33 (49.3)	144 (56.5)			0.05 [0.00 – 0.81] 0.035
31-46	34 (50.7)	67 (26.3)	22.19	<0.001	0.02 [0.00 – 0.36] 0.008
**Marital Status**					
Married	11 (16.4)	122 (47.8)			
Not married	45 (67.2)	122 (47.8)			0.25 [0.13 – 0.51] <0.001
Divorced	11 (16.4)	11 (4.4)	27.88	<0.001	0.09 [0.03 – 0.26] <0.001
**Educational status**					
None	11 (16.4)	22 (8.6)			
Basic	11 (16.4)	100 (39.2)			4.47 [1.75 – 11.39] 0.002
Secondary	34 (50.8)	77 (30.2)			1.15 [0.51 – 2.60] 0.740
Tertiary	11 (16.4)	56 (22.0)	18.43	<0.001	2.51 [0.96 – 6.50] 0.058
**Occupation**					
Farmer	11 (16.4)	45 (17.6)			
Trader	45 (67.2)	43 (56.1)			0.80 [0.38 - 1.65] 0.541
Civil servant	11 (16.4)	45 (17.7)			1 [0.40 – 2.50] 1.000
Others	0 (0.00)	22 (8.6)	6.99	0.072	11.37 [0.64–201.83] 0.098
**Religion**					
Christianity	33 (49.3)	222 (87.1)			
Islam	34 (50.7)	33 (12.9)	46.02	<0.001	0.15 [0.08 – 0.27] <0.001
**Ethnicity**					
Mo	0 (0.00)	100 (39.2)			
Gonja	22 (32.8)	11 (4.3)			0.01 [0.00 – 0.04] <0.001
Ewe	0 (0.00)	11 (4.3)			0.11 [0.00 – 6.04] 0.280
Akan	12 (17.9)	22 (8.7)			0.01 [0.00 – 0.16] 0.001
Dagaaba	33 (49.3)	111 (43.5)	76.00	<0.001	0.02 [0.00 – 0.27] 0.004
**Family type**					

Nuclear	33 (49.2)	255 (100)			
Extended	34 (50.7)	0 (0.00)	144.68	<0.001	0.01 [0.00 – 0.03] <0.001
**Number of children**					
≤ 2 children	45 (67.1)	200 (78.4)			
3-4 children	11 (16.4)	44 (17.3)			0.89 [0.42 – 1.81] 0.725
5+ children	11 (16.4)	11 (4.3)	12.29	0.002	0.23 [0.09 – 0.55] 0.001

Community and individual challenges affecting antenatal utilization

In this study, long distance from their homes to the health facility (31.1%) and their partner's perception of the importance of ANC were the leading barriers to the effective utilisation of ANC services by the women. Also, about 27.6% indicated not having enough money to attend antenatal clinic and cultural beliefs (7.9%) as some of the challenges. This is shown in (Figure 4).

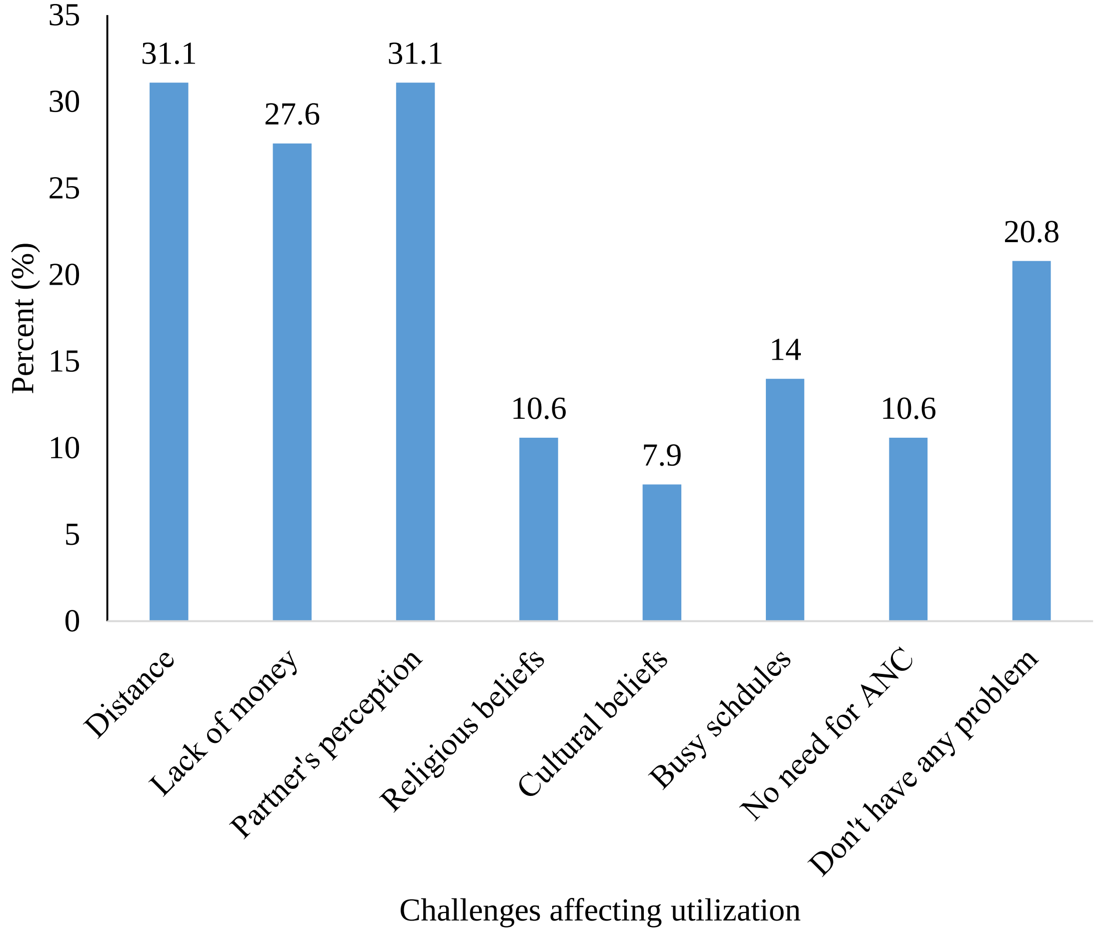

Figure 4: Challenges affecting utilization of ANC

Facility related barriers to ANC attendance

Figure 5 showed that most, 38.2% of the participants were faced with long waiting times in health facilities where they assessed care, followed by 28% who were scolded by staff and 24.5% who had inconvenient service hours.

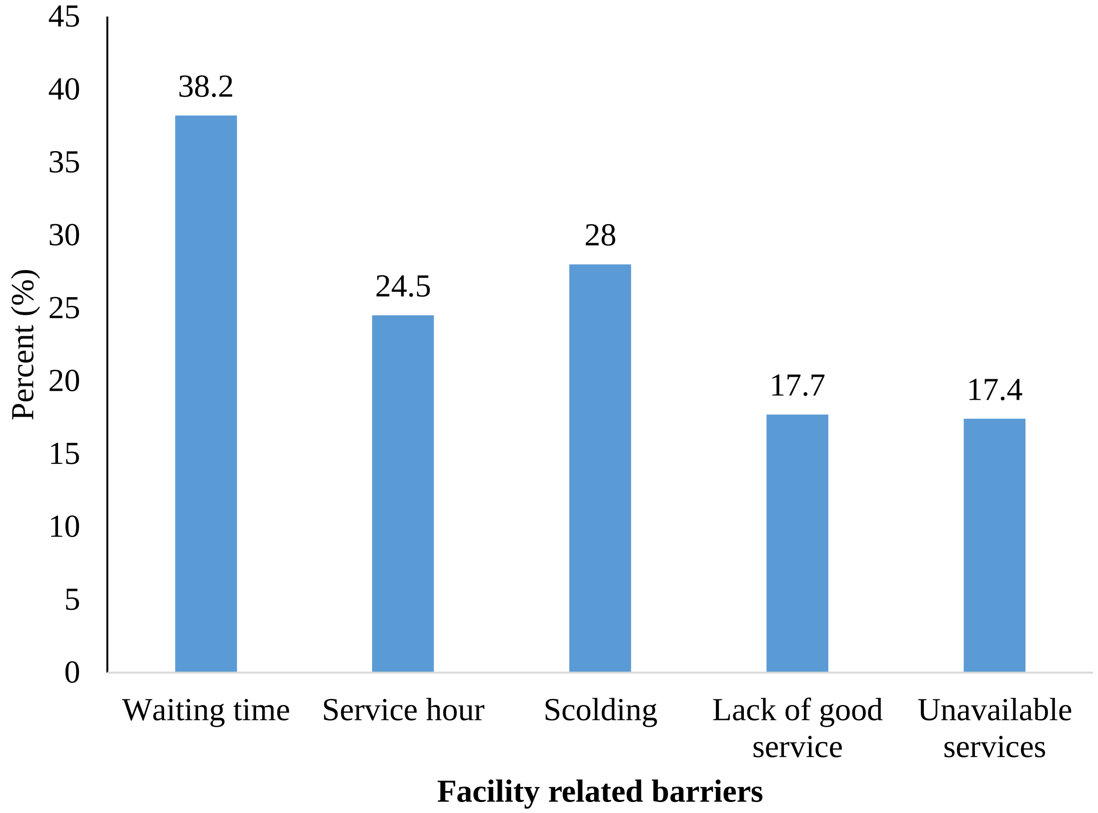

Figure 5: Facility related barriers to ANC utilization

Reasons for ANC Attendance

Figure 6 also showed that majority, 76.1% of the respondents went for antennal services for check-ups, while in 24.2% of them, it was because of normal routine during pregnancy and about one-tenth, 10.6% went for antenatal care services as a result of health workers' advice.

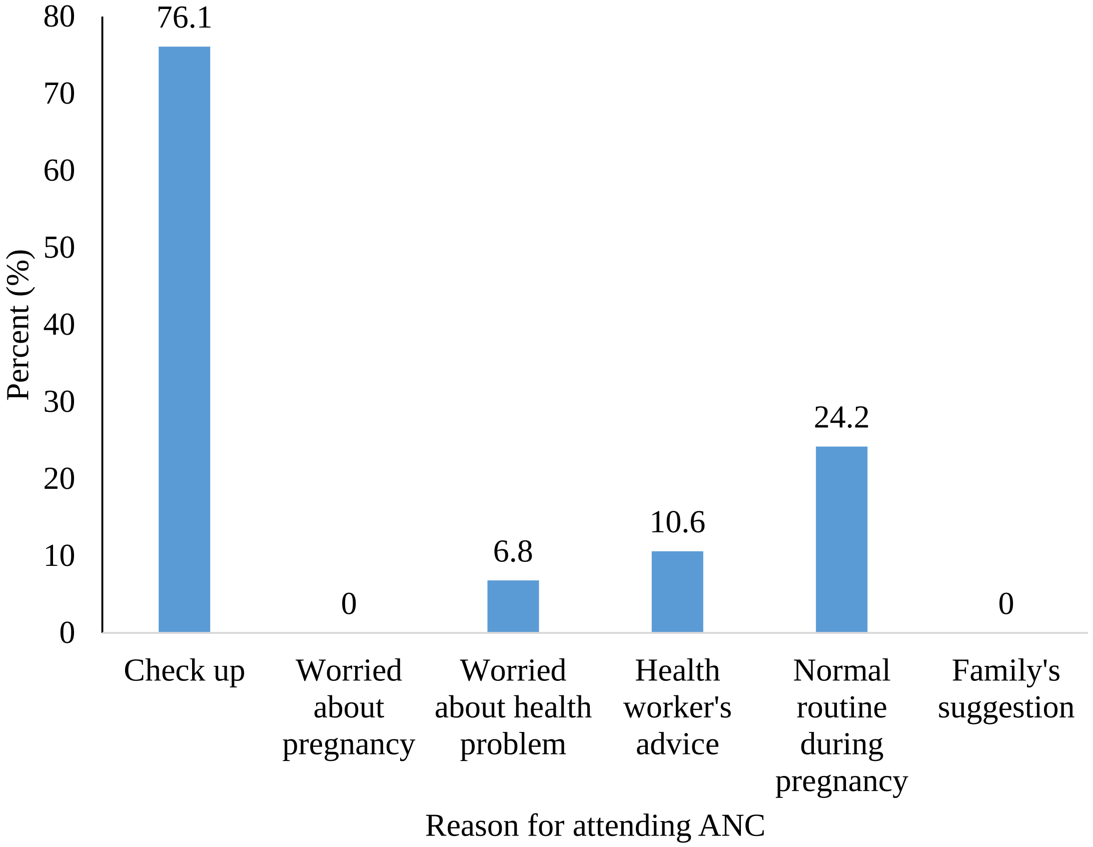

Figure 6: Reason for attending ANC

Discussion

The World Health Organization recommends four or more ANC visits for women with normal pregnancy [19]. The current study finding revealed that 69.0% of the women attended ANC at least four or more times. This study corroborates results in Ethiopia 66% [20] but higher than some prior studies in Rwanda 46% [3], Ethiopia 35.3% [21], and Kenya 52% [22]. Though the current result is high as compared to other countries, it is, however, lower than findings in previous studies in Ghana 86% [23], Nepal 87% [24] and Nigeria 81.5% [9]. This divergence could be due to socio-cultural, economic and awareness differences among the study populations as purported by other studies [21, 25]. The present finding of 69% is below the national coverage of at least four ANC visits of 75.9% [26]. Therefore, there is the need to intensify the interventions implemented by the Ghana Health Service over the years which include; Social and Behaviour Change Communication (SBCC) to create awareness (early initiation) and regular ANC visits, the Community-based Health Planning Services (CHPS) strategy to improve access to maternal health and capacity building for improved client-service provider relationship [26].

Participants' educational level was found to be a strong predictor of women utilizing ANC services for four or more times. Participants who had basic, secondary and tertiary education were more likely to use ANC services compared to those who had no education. This finding is not counter-intuitive because previous studies in developing countries have reported that educational level of women increases the odds of utilising ANC services for four or more times [9, 21, 27-30]. Probably the educated women are more inclined to issues of their health (identifying danger signs) thereby placing more priority on their health and that of the unborn child. The major cause of health inequality in ANC coverage has been strongly associated with

women’s poor/lack of education [31]. This highlights the need for the health sector to collaborate with the educational sector to promote female education to the highest level. Participants who were not married or divorced were less likely to attend ANC for at least four or more times relative to participants who were married. In a Ghanaian community, women who are not married are expected to remain virtuous until marriage [32] in order to prevent public ridicule, so women who get pregnant without a husband could avoid ANC visits [23] to prevent public ridicule. On the other hand, women without partners and without a sustainable job might have been experiencing financial challenges that may prevent them from utilizing ANC services.

Women who were insured with the NHIS were three times more likely to utilise ANC services for four or more times compared to those who were not insured. Generally, it is reported that the low socio-economic status of women results in poor health-seeking behavior and health outcomes. Instead, the current study found women with low socioeconomic status more likely to utilize ANC services at least four times due to their enrolment into the national health insurance scheme. The finding is consistent with other studies conducted in Ghana [23, 33-35], where the findings of these studies show the importance of health insurance in women seeking and receiving healthcare. The implementation of the free maternal health care policy under the NHIS instituted by the government of Ghana is key to achieving increased utilisation of ANC services for at least four times during every normal pregnancy [36]. In this policy, all pregnant women access ANC services until they deliver without having to pay for the services. Therefore, the call for all pregnant women to be insured by the government where they can access quality healthcare without paying for it is imperative.

Women who trek far distances to the health facility for ANC services were 80% less likely to utilise ANC services for four or more times compared to those with trek shorter distances [OR=0.20 (95%CI: 0.12-0.34), p<0.001]. The current study finding corroborates studies in Uganda and Pakistan where incomplete ANC attendance was attributed to long distance to the health facility [37, 38]. Establishment of health facilities within catchment areas in rural communities, employing more qualified health personnel to deliver healthcare to the women in the communities and access to good roads among others by the government of Ghana is significant in ensuring increased ANC utilisation.

Women's knowledge of ANC is crucial in the utilisation of ANC services during pregnancy. This study revealed that 79.2% of the women had good knowledge of ANC services. The determinants of women having knowledge of ANC services included; woman's age, woman's educational status, husband's educational status, religion, ethnicity and the number of children. Our study finding is consistent with a study conducted in the southwest of Nigeria [39] but disagrees with a study conducted in Mozambique where the researchers found women's knowledge of ANC services not to have significant importance in their utilisation of ANC services [40]. It is established that women with good knowledge of ANC services have a better understanding and acceptance of the services provided during ANC [41], and this knowledge will, therefore, be a catalyst for the utilising ANC services during pregnancy.

On the aspect of facility-based factors that hinders ANC utilisation, the study found that waiting for a long time in the facilities to receive care, was the major implicating factor for facility-based barriers to ANC utilisation. This finding is congruent with a study conducted in Mozambique where the women identified long waiting times as a barrier to ANC utilisation [41]. The long waiting time identified by participants as a barrier to ANC service utilisation could be due to the

shortage of qualified healthcare professionals needed to provide care to over 26 communities in the study setting. Pregnant women might find it so distressing waiting for long hours without receiving care and forgoing certain duties which might have financial implications on them and this may demotivate women for subsequent ANC service utilisation. Gong et al., [41] further support our assertion by illustrating that long waiting times have direct and indirect cost implications for clients seeking care and that attending to clients on schedule mitigates cost and also improves women accessing ANC services.

A major drawback of this study was that participants were purposively sampled based on their availability at the facilities without being randomly chosen. Notwithstanding, this study provides useful information on women's knowledge and factors that influence antenatal care utilisation in rural settings in Ghana.

Conclusion

Although the majority of the women had good knowledge of ANC services, a significant number of them do not still complete the recommended number of ANC visits for at least four times during a normal pregnancy. The creation of awareness of adequate ANC attendance in rural settings should be emphasized by health workers especially nurses and midwives working in these communities. Ghanaian women who were not registered with the NHIS and were economically disadvantaged had a lower odd of completing the recommended four visits by WHO. We, therefore, recommend that the government of Ghana should automatically enroll all pregnant women in rural settings into the NHIS.

Further research is recommended to determine sociocultural, traditional, religious and other related practices at the community level on the utilisation of antenatal care services.

Abreviations

ANC: Antenatal care

CHPS: Community-based Health Planning Services

GMHS: Ghana Maternal Health Survey

MMR: Maternal Mortality Ratio

NHIS: National Health Insurance scheme

SBCC: Social and Behaviour Change Communication

WHO: World Health Organisation

Conflict of interest

Authors declared no competing interest.

Data Availability Statement:

All relevant data are within the manuscript.

Funding

The study received no external funding

Acknowledgments

Our profound gratitude goes to the health facilities for their support and co-operation during data collection. We are also grateful to the women who took time off their busy schedules to participate in the study.

Author Contributions:

Conceptualization: Agani Afaya, Thomas B. Azongo, Veronica Millicent Dzomeku, Richard Adongo Afaya, Solomon Mohammed Salia

Data curation: Agani Afaya, Richard Adongo Afaya, Solomon Mohammed Salia

Formal analysis: Agani Afaya Peter Adatara, Robert Kaba Confidence alorse Atakro

Funding acquisition: No funding

Investigation: Agani Afaya Thomas B. Azongo, Veronica Millicent Dzomeku, Hyeonkyeong Lee, Richard Adongo Afaya, [1]Solomon Mohammed Salia, Peter Adatara, Robert Kaba, Confidence alorse Atakro, Paul Amuna

Methodology: Agani Afaya Thomas B. Azongo, Veronica Millicent Dzomeku, Hyeonkyeong Lee, Richard Adongo Afaya, [1]Solomon Mohammed Salia, Peter Adatara, Robert Kaba, Confidence alorse Atakro, Paul Amuna

Project administration: Agani Afaya Thomas B. Azongo, Veronica Millicent Dzomeku, Hyeonkyeong Lee, Richard Adongo Afaya, [1]Solomon Mohammed Salia, Peter Adatara, Robert Kaba, Confidence alorse Atakro, Paul Amuna

Resources: Agani Afaya, Richard Adongo Afaya

Software: Agani Afaya

Supervision: Agani Afaya, Thomas B. Azongo, Veronica Millicent Dzomeku, Hyeonkyeong Lee, Peter Adatara, Robert Kaba, Paul Amuna

Validation: Agani Afaya, Thomas B. Azongo, Veronica Millicent Dzomeku, Hyeonkyeong Lee, Peter Adatara, Robert Kaba, Paul Amuna

Visualization: Agani Afaya Thomas B. Azongo, Veronica Millicent Dzomeku, Hyeonkyeong Lee, Richard Adongo Afaya, [1]Solomon Mohammed Salia, Peter Adatara, Robert Kaba, Confidence alorse Atakro, Paul Amuna

Writing – original draft: Agani Afaya, Richard Adongo Afaya

Writing – review & editing: Agani Afaya Thomas B. Azongo, Veronica Millicent Dzomeku, Hyeonkyeong Lee, Richard Adongo Afaya, Solomon Mohammed Salia, Peter Adatara, Robert Kaba, Confidence alorse Atakro, Paul Amuna

Reference

1. World Health Organization. WHO recommendations on antenatal care for a positive pregnancy experience. World Health Organization; 2016.
2. World Health Organization. Trends in maternal mortality: 1990-2015: estimates from WHO, UNICEF, UNFPA, World Bank Group and the United Nations Population Division: executive summary. World Health Organization; 2015.
3. Rurangirwa AA, Mogren I, Nyirazinyoye L, Ntaganira J, Krantz G. Determinants of poor utilization of antenatal care services among recently delivered women in Rwanda; a population based study. BMC pregnancy and childbirth. 2017 Dec 1;17(1):142.
4. Gunawardena N, Bishwajit G, Yaya S. Facility-Based maternal death in Western Africa: a systematic review. Frontiers in public health. 2018 Feb 26;6:48.
5. Ghana Statistical Service (GSS), Ghana Health Service (GHS), and ICF. 2017 Ghana Maternal Health Survey Research Policy Briefs. Accra, Ghana: GSS, GHS, and ICF. 2019
6. UNICEF. Antenatal care. 2018. Available from; https://data.unicef.org/topic/maternal-health/antenatal-care/ (accessed March 7, 2019).
7. WHO: Global Health Observatory data repository**:** Antenatal care coverage Data by country Available from;

http://apps.who.int/gho/data/node.main.ANTENATALCARECOVERAGE4 (accessed 17 January 2020)

8. WHO: Global Health Observatory (GHO) data; Country specific: Available from; https://www.who.int/gho/countries/en/
9. Fagbamigbe AF, Idemudia ES. Barriers to antenatal care use in Nigeria: evidences from non-users and implications for maternal health programming. BMC pregnancy and childbirth. 2015 Dec 1;15(1):95.
10. Bustreo F, Say L, Koblinsky M, Pullum TW, Temmerman M, Pablos-Mendez A. Ending preventable maternal deaths: the time is now. Lancet, Glob Heal. 2013;1:e176–7.
11. Ashir GM, Doctor HV, Afenyadu GY. Performance based financing and uptake of maternal and child health services in yobe sate, northern Nigeria. Glob J Heal Sci. 2013;5:34–41
12. Hijazi HH, Alyahya MS, Sindiani AM, Saqan RS, Okour AM. Determinants of antenatal care attendance among women residing in highly disadvantaged communities in northern Jordan: a cross-sectional study. Reproductive health. 2018 Dec;15(1):106.
13. World Health Organization. Antenatal care in developing countries: promises, achievements and missed opportunities. 2003. Available from; http://apps.who.int/iris/bitstream/handle/10665/42784/9241590947.pdf?sequence=1.
14. Babitsch B, Gohl D, Von Lengerke T. Re-revisiting Andersen's Behavioral Model of Health Services Use: a systematic review of studies from 1998–2011. GMS Psycho-Social-Medicine. 2012;9.
15. Andersen RM. Revisiting the behavioural model and access to medical care: does it matter? J Health Soc Behav. 1995;3(6):1–10.

16. Andersen R, Newman JF. Societal and Individual Determinants of Medical Care Utilization in the United States. Milbank Q. 2005;83(4):1–28.
17. Ogunba BO, Abiodun OB. Knowledge and attitude of women and its influence on antenatal care attendance in Southwestern Nigeria. J Nutr Health Sci. 2017;4(2):207.
18. Yamane T. Statistics, An Introductory Analysis, 2nd Ed., 1967. New York: Harper and Row.
19. World Health Organization, United Nations Population Fund, United Nations International Children's Emergency Fund, The World Bank. Pregnancy, childbirth, postpartum and newborn care: a guide for essential practice (3rd edition). World Health Organization. Available from: http://www.who.int/maternal_child_adolescent/documents/924159084x/en/
20. Birmeta K, Dibaba Y, Woldeyohannes D. Determinants of maternal health care utilization in Holeta town, central Ethiopia. BMC Health Serv Res. 2013;13(1):256.
21. Ayalew TW, Nigatu AM. Focused antenatal care utilization and associated factors in Debre Tabor Town, northwest Ethiopia, 2017. BMC research notes. 2018 Dec 1;11(1):819.
22. Gitonga E. Determinants of focused antenatal care uptake among women in tharaka nithi county, Kenya. Adv Public Health. 2017;2017:4.
23. Sakeah E, Okawa S, Rexford Oduro A, Shibanuma A, Ansah E, Kikuchi K, Gyapong M, Owusu-Agyei S, Williams J, Debpuur C, Yeji F. Determinants of attending antenatal care at least four times in rural Ghana: analysis of a cross-sectional survey. Global health action. 2017 Jan 1;10(1):1291879.

24. Tuladhar H, Dhakal N. Impact of antenatal care on maternal and perinatal outcome: a study at Nepal Medical College Teaching Hospital. Nepal J Obstet Gynaecol. 2011;6(2):37–43.

25. Manyeh AK, Amu A, Williams J, Gyapong M. Factors associated with the timing of antenatal clinic attendance among first-time mothers in rural southern Ghana. BMC Pregnancy and Childbirth. 2020 Dec 1;20(1):47.

26. Ghana Health Service, Family Health Division, Annual Report (2016). https://www.ghanahealthservice.org/downloads/FHD_2016_ANNUAL_REPORT_Final_June%2019.2017%20nat%20final.pdf

27. Nebeb GT, Salgedo WB, Alemayehu YK. Antenatal care utilization in Debre Tabor, North West Ethiopia. Gynecol Obstet. 2015;5:339. https ://doi.org/10.4172/2161-0932.10003 39.

28. Kalule-Sabiti I, Amoateng AY, Ngake M. The effect of socio-demographic factors on the utilization of maternal health care services in Uganda. Afr Popul Stud. 2014;28(1):515–25.

29. Tiruaynet K, Muchie KF. Determinants of utilization of antenatal care services in Benishangul Gumuz Region, Western Ethiopia: a study based on demographic and health survey. BMC pregnancy and childbirth. 2019 Dec;19(1):115.

30. Okedo-Alex IN, Akamike IC, Ezeanosike OB, Uneke CJ. Determinants of antenatal care utilisation in sub-Saharan Africa: a systematic review. BMJ open. 2019 Oct 1;9(10):e031890.

31. Ayamolowo SJ, Irinoye O, Oladoyin MA. Job satisfaction and work environment of primary health care nurses in Ekiti state, Nigeria: an exploratory study. *Internatinal Journal of Caring Sciences.* 2013;6:531–42.

32. Anarfi KJ. To Change or not to change: obstacles and resistance to sexual behavioural change among the youth in Ghana in the era of AIDS. Res Rev. 2003; 19:27–45.

33. Nketiah-Amponsah E, Senadza B, Arthur E. Determinants of utilization of antenatal care services in developing countries: recent evidence from Ghana. Afr J Econ Manag Stud. 2013;4:58–73.

34. Amoakoh-Coleman M, Ansah EK, Agyepong IA, Grobbee DE, Kayode GA, Klipstein-Grobusch K. Predictors of skilled attendance at delivery among antenatal clinic attendants in Ghana: a cross-sectional study of population data. BMJ open. 2015 May 1;5(5):e007810. e007810.

35. Arthur E. Wealth and antenatal care use: implications for maternal health care utilisation in Ghana. Health Econ Rev. 2012;2:14.

36. Afaya A, Dzomeku VM, Baku EA, Afaya RA, Ofori M, Agyeibi S, Boateng F, Gamor RO, Gyasi-Kwofie E, Nyaledzigbor PP. Women's experiences of midwifery care immediately before and after caesarean section deliveries at a public Hospital in the Western Region of Ghana. BMC Pregnancy and Childbirth. 2020 Dec;20(1):1-9.

37. Kawungezi PC, AkiiBua D, Aleni C, Chitayi M, Niwaha A, Kazibwe A, Sunya E, Mumbere EW, Mutesi C, Tukei C, Kasangaki A. Attendance and utilization of antenatal care (ANC) services: multi-center study in upcountry areas of Uganda. Open journal of preventive medicine. 2015 Mar 1;5(3):132.

38. Ali SA, Dero AA, Ali SA. Factors affecting the utilization of antenatal care among pregnant women: a literature review. J Preg Neonatal Med 2018; 2 (2): 41-45. 42 J Preg Neonatal Med 2018 Volume 2 Issue. 2018;2.
39. Fagbamigbe AF, Akanbiemu FA, Adebowale AS, Manuwa-Olumide A, Korter G. Practice, knowledge and perceptions of antenatal care services among pregnant women and nursing mothers in Southwest Nigeria. International Journal of Maternal and Child Health. 2013;1(1):7-16.
40. Gong E, Dula J, Alberto C, de Albuquerque A, Steenland M, Fernandes Q, Cuco RM, Sequeira S, Chicumbe S, Gudo ES, McConnell M. Client experiences with antenatal care waiting times in southern Mozambique. BMC health services research. 2019 Dec;19(1):538.
41. Gupta RK, Shora TN, Verma AK, Jan R. Knowledge regarding antenatal care services, its utilization, and delivery practices in mothers (aged 15-49 years) in a rural area of North India. Tropical Journal of Medical Research. 2015 Jul 1;18(2):89.
