# Supplementary material for: Women’s knowledge and its associated factors regarding optimum utilisation of antenatal care in rural Ghana: A cross-sectional study
Source: PLoS One. 2020 Jul 9;15(7):e0234575. doi: 10.1371/journal.pone.0234575 (PMC7347137; doi:10.1371/journal.pone.0234575)
Supplement: Attachment — Submitted filename: PONE-D-20-05929_R1 AA review.pdf [file pone.0234575.s004.pdf]

PLOS ONE

Women's knowledge and its associated factors regarding optimum utilisation of antenatal care in rural Ghana: a cross-sectional study

--Manuscript Draft--

**Manuscript Number:**	PONE-D-20-05929R1
**Article Type:**	Research Article
**Full Title:**	Women's knowledge and its associated factors regarding optimum utilisation of antenatal care in rural Ghana: a cross-sectional study
**Short Title:**	Women's knowledge and its associated factors regarding optimum utilisation of antenatal care in rural Ghana
**Corresponding Author:**	Agani Afaya<br>University of Health and Allied Sciences<br>Ho, GHANA
**Keywords:**	antenatal care utilization
**Abstract:**	Introduction: Improving maternal health is a global public health challenge especially in sub-Saharan Africa. The optimum utilisation of antenatal care by pregnant women is known to improve maternal health outcomes. Maternal morbidity and mortality rates in Ghana remain unacceptably high, particularly in rural settings where skilled delivery care often times is disproportionally low. This study assessed factors associated with optimum utilisation of antenatal care in rural Ghana.<br>Methods: A cross-sectional design was applied to collect data among eligible participants between October 2018 and January 2019. A total of 322 women who gave birth and attended the postnatal clinic were recruited for the study. Consecutive sampling was employed in recruiting participants. The associations between the dependent variables (ANC service utilization and knowledge of ANC) and independent variables (socio-demographic characteristics) were examined using ordinary least squares logistic regression at 95% confidence interval in STATA version 14.0.<br>Results: Of the 322 participants, 69.0% reported utilising at least four or more times ANC services. Determinants of women attending ANC for four or more times was significantly associated with age [OR=4.36 (95%CI: 2.16-8.80), p<0.001], educational level [OR=10.18 (95%CI: 3.86-26.87), p<0.001], and insured with National Health Insurance Scheme [OR=3.42 (95%CI: 1.72-6.82), p<0.001]. Not married [OR=0.65 (0.39 - 1.09), p=0.011] or divorced [OR=0.33 (95%CI: 0.13 – 0.83), p=0.019] was negatively associated with utilisation of four or more ANC services. The majority (79.0%) of the participants had a good level of knowledge regarding antenatal care.<br>Conclusion: Although the majority of women in this study had good knowledge of ANC services, a significant number of them did not complete the recommended number of ANC visits for at least four times during a normal pregnancy. Awareness and further education to reproductive-age women on the significant role adequate ANC attendance plays in advancing health and well-being require further investments, particularly among rural women in Ghana.
**Order of Authors:**	Agani Afaya
	Thomas Bavo Azongo
	Veronica Millicent Dzomeku
	Hyeonkyeong Lee
	Richard Adongo Afaya
	Solomon Mohammed Salia
	Peter Adatara
	Robert Kaba
	Martin Amogre Ayanoore
	Confidence alorse Atakro

Powered by Editorial Manager® and ProduXion Manager® from Aries Systems Corporation

	David Adadem
	Emmanuel Opoku Asiedu
	Paul Amuna
	Abigail Kusi-Amponsah Diji
Response to Reviewers:	PONE-D-20-05929<br>Women's knowledge and its associated factors regarding optimum utilisation of antenatal care in rural Ghana: a cross-sectional study<br>Agani Afaya (MSc), Thomas B. Azongo (PhD), Veronica Millicent Dzomeku (PhD), Hyeonkyeong Lee (PhD), Richard Adongo Afaya (MPhil), Solomon Mohammed Salia (MSc), Peter Adatara (PhD), Robert Kaba Alhassan (PhD), 6Martin Amogre Ayanore (PhD), Abigail Kusi-Amponsah Diji, Confidence Alorse Atakro (MN), David Adadem (BN),Emmanuel Opoku Asiedu (BN), Paul Amuna (MB ChB, PhD)<br><br>Dear Editor,<br>We would like to thank you sincerely for the insightful reviewer comments on our manuscript and for the opportunity to resubmit the manuscript for another round of review. We find the comments very useful and have responded to them to the best of our knowledge. We acknowledge that the comments have no doubt helped improve the quality of our manuscript.<br>We herein provide further details by showing a point-by-point feedback on how each of the comments received were addressed. For easy identification, the reviewers' comments have been repeated while Authors' responses appear in BOLD text.<br>Amendment of Authorship<br>I would like to amend authorship to include Dr. Abigail Kusi-Amponsah Diji who contributed to conceiving and designing the study and contributed enormously to this paper. Her name has been included in the manuscript and highlighted in yellow ink.<br>I apologise as this omission occurred during the initial submission process.<br>Thank you.<br>Review comments<br><br>Reviewer #1<br>Review comments<br>1. Line numbering would have made it easy to referencing.<br>Authors response<br>The Manuscript was formatted according to the Journal specifications. However, in view of the comment, line numbering has been included in the revised manuscript for easy referencing.<br><br>Review comment<br>The statement "The sub-district has semi-urban area but predominantly rural" under study design section should be put under study settings.<br>Authors response<br>Changes have been affected as suggested and now appears in the revised manuscript in page 8, lines 154-155.<br><br>Review comment<br>3. The variable "Number of Children" can't be dichotomy when the categories are more than two. "The number of children was dichotomised into 1-2, 3-4 and 5+ children"<br>Authors response<br>The sentence has been revised: dichotomized has been changed to categorized. This is can be found in page 10, line 189 of the revised manuscript.<br><br>Review comments<br>4. How was the distance to health facility measured? Distance from where to health facility? What distance is categorized as "far" and what is "near"?<br>Authors response<br>For CHPS, the distance was between a radius of 5 km (GHS, 2002; MoH, 2016) and for Health Centres between 8 and 16 km radius (MEST, 2011). Women who trekked more than 5km and also 16km where considered far and those within the recommended limit were considered short. See page 10 lines 191-193.<br>[Ministry of Health, (2016). National Community-Based Health Planning and Services Policy. Ghana, Accra.

	Ghana Health Service (GHS), (2002). The Community-Based Health Planning and Services initiative: Concepts and plans for implementation. Accra, Ghana.<br>Ministry of Environment Science and Technology (MEST), 2011. Zoning guidelines and planning standards.<br><br>Review comments<br>5. “Those who sought ANC services less than four times were classified as having poor ANC service utilisation (inadequate) and those who had ANC visits of 4 or more were considered as having good (adequate) ANC service utilisation.” This information has been captured under the section on outcome variables already. Please delete it.<br>Authors response<br>Repetition deleted under data analysis section see page 10, lines…<br><br>Review comment<br>6. Revise this statement: “Bivariate logistic regression analysis, computing odds ratio, was used to determine the strength of the association…” Because Logistic regression will report odds ratio. Bivariate logistic regression analysis reports more than strength of association. What about direction of the association?<br>Authors response<br>In page , lines this has been revised and now reads as; “Ordinary least square logistic regression analysis was applied where odds ratios were computed to determine how the independent variables is associated with the dependent variables (ANC service utilisation and knowledge of ANC)”<br><br>Review comments<br>7. The statement “ANC service utilisation was coded as 0 for poor utilisation (<4 ANC visits) and 1 for good untilisation (4+ ANC visits)” has been repeated under the outcome variables and twice under data analysis sections. Please check and delete where appropriate.<br>Authors response<br>The repetition highlighted by the reviewer under data analysis have been deleted in the revised submission.<br><br>Review comments<br>8. Please provide the Ethics approval number.<br>Authors response<br>This has been provided in page line and now reads as “Ethics approval for the study was obtained from the University of Health and Allied Sciences Research Ethical Committee (UHAS-REC A. 10[27] 17-18)”.<br><br>Review comments<br>9. What is purpose of running a chi-square test and crude logistic regression as reported in table 2?<br>Authors response<br>Chi-square results has been removed and replaced with tables only highlighting analysis of logistic regression (for adjusted and unadjusted)<br><br>Review comments<br>10. Table 2 is titled “Regression analysis results for ANC attendance” but reported Chi-square results in addition. I suggest the authors present the results of the logistics regression only in tables 2 and 3<br>Authors response<br>As suggested by the reviewer, authors have re-presented the results of the logistics regression only in tables 2 and 3.<br><br>Review comments<br>11. How were confounding effects accounted for in establishing the association between the dependent and independent variables? Think the authors have to explore association in presence of other explanatory variables. This part of the analysis is not clear.<br>Authors response<br>National health insurance was controlled for in the adjusted model. When NHIS was controlled for in the adjusted model there was a significant relationship with optimum ANC and these explanatory variables (Age, marital status, educational level, religion,

Powered by Editorial Manager® and ProduXion Manager® from Aries Systems Corporation

and distance to health facility). There for NHIS was found to have confounder effect when included in the regression model.
Review comments
12. How was the Family type defined? how was the question asked?
Authors response
Nuclear family was termed as a family group consisting of two parents and their children (one or more). Extended family: consisting of parents like father, mother, and their children, aunts, uncles, grandparents, and cousins, all living in the same household.
Do you live with your husband and children alone or with your other extended family (grandfather etc)

Review comments
13. In Figure 6, how is “Check-up” different from “Normal routine during pregnancy”? How were those two questions asked?
Authors response
Check-up: the main reason behind they attending ANC was to check their health status and that of their unborn baby. “Normal routine during pregnancy” mothers that were categorized here where those who mainly said their reason for attending ANC was because it is the routine thing to do during pregnancy and has been asked to come on this date by nurses.

Review comments
14. The statistical association cannot be strongly established with only crude results without accounting for explanatory variables. I suggest a revision of the statistical analysis to inform the conclusion of the study.
Authors response
Reviewer concerns have been addressed in table 2 and 3

Reviewer # 2

Review comments
The study seems to assess optimal utilization (four or more visits) of ANC among utilizers of ANC and not utilization of ANC as suggested by the topic.
Authors response
Taking the reviewer suggestion into consideration, authors have revised the title as found in the revised manuscript submitted. See page 1 lines 2-3

Review comments
Also, the authors should be adjusted the title to reflect that they only assessed socio-demographic factors.
Authors response
While acknowledging that this is a fair comment from reviewers, other factors outside individual socio-demographic factors were assessed i.e community and individual challenges affecting antenatal utilization; facility related barriers to ANC attendance; reasons for ANC Attendance etc. Hence, adjusting the title to reflect socio-demographic factors has a potential to limit the scope of factors considered in this study.
Given the title change in this revised submission, authors think we have adequately addressed any concerns regarding the suitability of the title and that the title now reflects the aim/objectives of the study.

Introduction:
review comment
Some of the text require references.
Authors response
References have been provided in relevant areas in the introduction section.

Review comment
The justification is not compelling enough. According to you, ANC attendance is high but utilization for delivery is low. Isn't this a more interesting topic than exploring factors for ANC attendance? The authors need to better justify the investigation.
Authors response
This has been addressed in page 4 and 5 lines 89-104. The highlighted portions

	provide further clarity on areas these have been addressed.<br><br>Review comments<br>Conceptual framework:<br>Authors had a beautiful conceptual framework but limited the investigation to a few socio-demographic correlates of optimal ANC attendance. The reason for this is not clear.<br>The authors seem to suggest that attendance of four or more ANC visits is synonymous with utilization rather than optimal utilization.<br><br>Authors response<br>We adapted the behavioural model framework of Andersen for use of health services to identify the factors that potentially facilitate or impede minimum number of antenatal health services seeking behavior at individuals and community levels.<br><br>In considering the review suggestion, authors have made changes and have maintained optimal utilisation instead of utilisation in this revised submission of the manuscript.<br><br>Review comments<br>Could the authors briefly explain the reasons for its (Anderson Behavioral Health care model) wide applicability? Why choose this model for the inquiry? Why not any other model?<br>Authors response<br>The frame work is widely used because the three factors identified by Andersons model to facilitate or impede healthcare access cuts across almost every health system across the globe including the developed and developing countries. And several studies have used the model to identify facilitating and impeding factors to healthcare access.<br><br>The framework predicts that a series of factors predisposing, enabling and need factors influence the utilization of health services by people. According to the model, predisposing factors are demographics and social structures. Enabling factors facilitates individuals to use services for example, availability of resources such as income, access to free services, availability and access to the service. Need factors motivates service use. We chose this model because we sought to determine factors that impede or facilitate ANC services utilization in health facilities. And Anderson behavioral model best suit our current study objective.<br><br>Methods:<br>Review comments<br>Study design:<br>I am not convinced that the method is adequate to answer the research question. Assessing factors related to utilization of ANC services would be better answered by comparing utilizers and non-utilizers. Rather the design addresses correlates of optimum utilization (four or more) among utilizers. The authors need to clarify this.<br>Authors response<br>Reviewer suggestion and comments was helpful in making revisions to the design section of the revised manuscript. This study truly addresses correlates of optimum utilization among users and this has reflected in the revised manuscript under the study design section. See page 8 lines 137-140.<br>Outcome variable:<br>Review comments<br>How many outcome variables did this study have? The first sentence suggests more than one but itemizes just one. Kindly correct.<br>Authors response<br>The outcome variables were optimum utilisation and Knowledge of ANC. This has been addressed under outcome variables. See page 9 lines 172-173.<br>Review comments<br>The authors should use “good” or “bad” knowledge for the sake of consistency and not knowledgeable or not.

Powered by Editorial Manager® and ProduXion Manager® from Aries Systems Corporation

Authors response
Authors acknowledge this as a fair suggestion. However, to ensure consistency in the revised manuscript, authors have used good and poor knowledge.

Review comments
The characterization of participants seems unclear. A part of the manuscript suggests that 50% was used as cut-off, while another part suggests that the participants' mean score was used as cut-off. Kindly clarify and justify.
Authors response
Authors acknowledge the inconsistent reporting in the methods section with regard to the cut-off point in knowledge assessment. Author has corrected this inconsistency by deleting the 50% cut-off point.

Review comments
Validity and reliability of the study instrument:
How was construct validity assured?
Authors response
construct validity pertains to a specific use of a scale and can often be context or population dependent. The current study instrument was contextually structured to identify factors that facilitate or impede optimal utilization of ANC services within the study population.

Multi-collinearity diagnostics were done and found that the Variance Inflation Factors (VIFs) were all below the 5-10 rule of thumb range, suggesting there is no collinearity among the independent variables to be fitted in the regression model! Effects of multi-collinearity was therefore ruled out and did not have any effect on the validity of the study outcomes.

Review comments
What is the level of expertise of these researchers and clinicians? It may be helpful to state their qualification and give further background about their experience.
Authors response
Thomas B. Azongo (PhD) is a snr lecturer and a public health expert; Veronica Millicent Dzomeku (PhD) is a midwife and a Snr Lecturer, Hyeonkyeong Lee (PhD) is a professor in community health nursing; Peter Adatara (PhD) is a snr lecturer and his specialty area is in maternal and child health; Robert Kaba Alhassan (PhD), Snr research fellow; Martin Amogre Ayanore (PhD) is a Health Economist and a Public health expert, Paul Amuna (MB ChB, PhD) is a professor and his area of specialty is maternal and child health
Clinicians: They both have degree in midwifery and have been practicing midwifery for over 10 years

Review comments
Questions 5, 6, 8, 9, 10 do not actually test what the women know?
Authors response
These questions were added to assess mothers knowledge on the other activities carried during ANC: Mothers that utilize ANC are taught these and they are expected to have basic knowledge in relation to the education or things taught during ANC attendance: for example during ANC, mothers are informed of the recommended ANC visits, so we expected mothers to know these. Additionally, mothers who utilized ANC are educated on the recommended place for safe delivery and the necessary items required for delivery. The items for assessing the mother's knowledge of ANC was developed in context with the study population

Review comments
Ethical consideration:
Could you include the reference number for the IRB approval?
Authors response
Ethics approval for the study was obtained from the University of Health and Allied Sciences Research Ethical Committee (UHAS-REC A. 10[27] 17-18).

Results:
Review comments
Authors did not report results of bivariate analysis despite proposing to do them in the

	methods section.<br>Authors response<br>The results section has been updated and review concerns above addressed. See table 2 and 3 on pages 16 &17 lines 284-285<br><br>Review comment<br>There is a need to report the salient findings even if all has been presented on table 2.<br>Authors response<br>Authors acknowledge this and have done the needful by focusing on only the salient findings. See page 15 lines 278-283.<br><br>Review comment<br>What is the mean age of the participants?<br>Authors response<br>Age of participants were categorized during data collection therefore the mean age could not be calculated.<br><br>Review comments<br>The title of table 2 need to be updated to bivariate and regression analysis. The authors need to make some comments about results of bivariate analysis in prose.<br>Authors response<br>Table 2 and 3 have been updated.<br><br>Review comments<br>Figure 3 is better presented as a pie chart.<br>Authors response<br>Figure 3 has been changed to a pie chart. Refer to page 18.<br><br>Review comment<br>On table 3 and age factor, if the reference group is the first group, then the Odds ratio need to be reviewed.<br>Authors response<br>This critical suggestion was reviewed and significant changes made to the odds ratios. Table 3 provide further details on the revised submission after addressing this concerns. See page 20.<br><br>Review comments<br>For figure 6, kindly remove the bars with zero responses.<br>Authors response<br>Changes has been made as suggested. Refer to page 14<br><br>Discussion:<br><br>Review comment<br>“The present finding of 69% is below the national coverage of at least four ANC visits of 75.9%”. Is there something about the study population that could account for this disparity?<br>Authors response<br>This has been addressed in page 25 line 348-351 and now reads as;<br>This difference may be due to the lack of awareness of optimal ANC visits. Also, the differences could have been caused by other potential inhibiting factors such as distance to health facilities and socio-economic factors.<br><br>Review comments<br>“Our study finding is consistent with a study conducted in the southwest of Nigeria [39] but disagrees with a study conducted in Mozambique where the researchers found women’s knowledge of ANC services not to have significant importance in their utilisation of ANC services [40]. It is established that women with good knowledge of ANC services have a better understanding and acceptance of the services provided during ANC [41], and this knowledge will, therefore, be a catalyst for the utilising ANC services during pregnancy.” It seems more plausible that utilization of ANC services would result in better knowledge of ANC not the other way round as suggested by the authors. Anyway, the study is cross-sectional, hence the use of the word determinant is better replaced with association.

Authors response
We totally agree with assertion (It seems more plausible that utilisation of ANC services would result in better knowledge of ANC). In the discussion section, this emphasizes has been made in relation to the findings of the study.

Limitations:
Review comments
I can immediately think of some other limitations of this study e.g. cross-sectional nature, therefore, just associations can be measured, bias due to self-report, non-exploration of many other factors, etc. Kindly develop this section further.
Authors response
A section on limitations has been added and some potential limitation of the study acknowledged. See page 29 lines 448-458

Review comments
Other comments"
I have made some further suggestions on the text of manuscript
Authors response
Review concerns on inconsistencies in the manuscript have been addressed. Authors read thoroughly through the revised manuscript to identify these inconsistencies and sentences that were not clear and corrections effected.

Reviewer #3:
Manuscript Title
Women's knowledge of and factors associated with the utilization of antenatal care in rural Ghana: analysis of a community-based cross-sectional study

Review

General Comments
Organization of the various sections need clarity and simplification of sentences. all sections must conform to the STROBE checklist for presenting prevalence studies.
Authors response
We acknowledge and used the STROBE checklist to guide the study (methods)

Specific Comments
Review comments
• Study Setting- is this a community based or facility-based study? The title says community but the methods state facility based.
Authors response
The title has been revised to reflect these concerns. See page 1
Review comment
• Questionnaire administration- were the questionnaires self-administered or was administered by a research assistant or both?
Authors response
The questionnaires were administered by only the four research assistants trained for data collection

Review comment
• Table 2 needs to be simplified, Summarized; the detailed version can be attached as an appendix.
Authors response
Authors acknowledge and have addressed it. Authors reported on only salient findings of the study but not every variable see table 2 on pages 16 & 17 lines 284-285.

Review comment
• "Instead, the current study found women with low socioeconomic status more likely to utilize ANC services at least four times due to their enrolment into the national health insurance

Powered by Editorial Manager® and ProduXion Manager® from Aries Systems Corporation

	scheme" where was this result presented?<br>Authors response<br>This was a general assumption for the study participants. This have been deleted because it lacked evidence from the current study.<br><br>Review comments<br>Are there any reasons why Bole had a lower than National ANC4+, What is the regional average?<br>Authors response<br>The difference may be due to the lack of awareness of optimal ANC visits. Also, the differences could have been caused by other potential inhibiting factors such as distance to health facilities and socio-economic factors. Refer to page 25 line 348-351.
**Additional Information:**	
**Question**	**Response**
**Financial Disclosure**<br><br>Enter a financial disclosure statement that describes the sources of funding for the work included in this submission. Review the submission guidelines for detailed requirements. View published research articles from *PLOS ONE* for specific examples.<br><br>This statement is required for submission and **will appear in the published article** if the submission is accepted. Please make sure it is accurate.<br><br>**Unfunded studies**<br>Enter: *The author(s) received no specific funding for this work.*<br><br>**Funded studies**<br>Enter a statement with the following details:<br>• Initials of the authors who received each award<br>• Grant numbers awarded to each author<br>• The full name of each funder<br>• URL of each funder website<br>• Did the sponsors or funders play any role in the study design, data collection and analysis, decision to publish, or preparation of the manuscript?<br>• **NO** - Include this sentence at the end of your statement: *The funders had no role in study design, data collection and analysis, decision to publish, or preparation of the manuscript.*<br>• **YES** - Specify the role(s) played.<br><br>* typeset	The author(s) received no specific funding for this work.
**Competing Interests**	The authors have declared that no competing interests exist

Use the instructions below to enter a competing interest statement for this submission. On behalf of all authors, disclose any competing interests that could be perceived to bias this work—acknowledging all financial support and any other relevant financial or non-financial competing interests.<br><br>This statement **will appear in the published article** if the submission is accepted. Please make sure it is accurate. View published research articles from *PLOS ONE* for specific examples.<br><br>**NO authors have competing interests**<br><br>Enter: *The authors have declared that no competing interests exist.*<br><br>**Authors with competing interests**<br><br>Enter competing interest details beginning with this statement:<br><br>*I have read the journal's policy and the authors of this manuscript have the following competing interests: [insert competing interests here]*<br><br>* typeset	
**Ethics Statement**<br><br>Enter an ethics statement for this submission. This statement is required if the study involved:<br><br>• Human participants<br>• Human specimens or tissue<br>• Vertebrate animals or cephalopods<br>• Vertebrate embryos or tissues<br>• Field research<br><br>Write "N/A" if the submission does not require an ethics statement.<br><br>General guidance is provided below. Consult the submission guidelines for	Ethical clearance was obtained from the University of Health and Allied Sciences Research Ethical Committee

detailed instructions. **Make sure that all information entered here is included in the Methods section of the manuscript.**<br><br>**Format for specific study types**<br><br>**Human Subject Research (involving human participants and/or tissue)**<br>• Give the name of the institutional review board or ethics committee that approved the study<br>• Include the approval number and/or a statement indicating approval of this research<br>• Indicate the form of consent obtained (written/oral) or the reason that consent was not obtained (e.g. the data were analyzed anonymously)<br><br>**Animal Research (involving vertebrate animals, embryos or tissues)**<br>• Provide the name of the Institutional Animal Care and Use Committee (IACUC) or other relevant ethics board that reviewed the study protocol, and indicate whether they approved this research or granted a formal waiver of ethical approval<br>• Include an approval number if one was obtained<br>• If the study involved *non-human primates*, add *additional details* about animal welfare and steps taken to ameliorate suffering<br>• If anesthesia, euthanasia, or any kind of animal sacrifice is part of the study, include briefly which substances and/or methods were applied<br><br>**Field Research**<br><br>Include the following details if this study involves the collection of plant, animal, or other materials from a natural setting:<br>• Field permit number<br>• Name of the institution or relevant body that granted permission	
**Data Availability**<br><br>Authors are required to make all data underlying the findings described fully available, without restriction, and from the time of publication. PLOS allows rare	Yes - all data are fully available without restriction

exceptions to address legal and ethical concerns. See the PLOS Data Policy and FAQ for detailed information.<br><br>A Data Availability Statement describing where the data can be found is required at submission. Your answers to this question constitute the Data Availability Statement and **will be published in the article**, if accepted.<br><br>**Important:** Stating 'data available on request from the author' is not sufficient. If your data are only available upon request, select 'No' for the first question and explain your exceptional situation in the text box.<br><br>Do the authors confirm that all data underlying the findings described in their manuscript are fully available without restriction?	
**Describe where the data may be found in full sentences. If you are copying our sample text, replace any instances of XXX with the appropriate details.**<br><br>• If the data are **held or will be held in a public repository**, include URLs, accession numbers or DOIs. If this information will only be available after acceptance, indicate this by ticking the box below. For example: *All XXX files are available from the XXX database (accession number(s) XXX, XXX.*).<br>• If the data are all contained **within the manuscript and/or Supporting Information files**, enter the following: *All relevant data are within the manuscript and its Supporting Information files.*<br>• If neither of these applies but you are able to provide **details of access elsewhere**, with or without limitations, please do so. For example:<br><br>*Data cannot be shared publicly because of [XXX]. Data are available from the XXX Institutional Data Access / Ethics Committee (contact via XXX) for researchers who meet the criteria for*	All relevant data are within the manuscript and its Supporting Information files.

access to confidential data.<br><br>*The data underlying the results presented in the study are available from (include the name of the third party and contact information or URL).*<br>• This text is appropriate if the data are owned by a third party and authors do not have permission to share the data.<br><br>* typeset	
Additional data availability information:	

Powered by Editorial Manager® and ProduXion Manager® from Aries Systems Corporation

Manuscript
Click here to access/download;Manuscript;Manuscript.docx

Title page

Women's knowledge and its associated factors regarding optimum utilisation of antenatal care in rural Ghana: a cross-sectional study

[1]Agani Afaya (MSc) [2]Thomas Bavo Azongo (PhD), [3]Veronica Millicent Dzomeku (PhD), [4]Hyeonkyeong Lee (PhD), [3]Richard Adongo Afaya (MPhil), [1]Solomon Mohammed Salia (MSc), [1]Peter Adatara (PhD), [5]Robert Kaba Alhassan (PhD), [6]Martin Amogre Ayanore (PhD), [3]Abigail Kusi-Amponsah Diji[1] (PhD) [7]Confidence Alorse Atakro (MN), [1]David Adadem (BN),[1]Emmanuel Opoku Asiedu (BN), [6]Paul Amuna (MB ChB, PhD)

[1]Department of Nursing, School of Nursing and Midwifery, University of Health and Allied Sciences, Ho, Ghana
[2]Department of Public Health, School of Allied Health Sciences, University for Development Studies, Tamale, Ghana
[3]Department of Nursing, Kwame Nkrumah University of Science and Technology, Kumasi, Ghana
[4]College of Nursing, Yonsei University, Seoul, South Korea
[5]Centre for Health Policy and Implementation Research. Institute of Health Research University of Health and Allied Sciences, Ho, Ghana
[6] Department of Health Policy Planning and Management, School of Public Health, University of Health and Allied Sciences, Ho, Ghana
[7]School of Nursing and Midwifery, Christian Service University College, Kumasi, Ghana

Correspondence

Agani Afaya (aagani@uhas.edu.gh)
Department of Nursing, School of Nursing and Midwifery, University of Health and Allied Sciences, Ho, Ghana

Abstract

Introduction: Improving maternal health is a global public health challenge especially in sub-Saharan Africa. The optimum utilisation of antenatal care by pregnant women is known to improve maternal health outcomes. Maternal morbidity and mortality rates in Ghana remain unacceptably high, particularly in rural settings where skilled delivery care often times is disproportionally low. This study assessed factors associated with optimum utilisation of antenatal care in rural Ghana.

Methods: A cross-sectional design was applied to collect data among eligible participants between October 2018 and January 2019. A total of 322 women who gave birth and attended the postnatal clinic were recruited for the study. Consecutive sampling was employed in recruiting participants. The associations between the dependent variables (ANC service utilization and knowledge of ANC) and independent variables (socio-demographic characteristics) were examined using ordinary least squares logistic regression at 95% confidence interval in STATA version 14.0.

Results: Of the 322 participants, 69.0% reported utilising at least four or more times ANC services. Determinants of women attending ANC for four or more times was significantly associated with age [OR=4.36 (95%CI: 2.16-8.80), p<0.001], educational level [OR=10.18 (95%CI: 3.86-26.87), p<0.001], and insured with National Health Insurance Scheme [OR=3.42 (95%CI: 1.72-6.82), p<0.001]. Not married [OR=0.65 (0.39 - 1.09), p=0.011] or divorced [OR=0.33 (95%CI: 0.13 – 0.83), p=0.019] was negatively associated with utilisation of four or more ANC services. The majority (79.0%) of the participants had a good level of knowledge regarding antenatal care.

Conclusion: Although the majority of women in this study had good knowledge of ANC services, a significant number of them did not complete the recommended number of ANC visits for at least four times during a normal pregnancy. Awareness and further education to

reproductive-age women on the significant role adequate ANC attendance plays in advancing health and well-being require further investments, particularly among rural women in Ghana.

Keywords Antenatal care utilisation, women's knowledge, factors, rural setting, Ghana

Introduction

Globally, about 303, 000 women and adolescent girls die as a result of pregnancy and childbirth-related complications [1] of which an alarming 99% of these maternal deaths occur in low-resource settings. Though globally, the number of women and girls dying due to pregnancy-related complications and childbirth decreased by nearly half (50%) from 1990 to 2013, the number of deaths within the West African region remains unacceptably high with Maternal Mortality Ratio (MMR) of 679 deaths per 100,000 live births in 2015 [2-4].

According to the 2017 Ghana Maternal Health Survey (GMHS), the MMR in Ghana is 310 per 100,000 live births, which remains unacceptably high. Data from the 2017 GMHS shows that nearly half of the women (50%) in rural settings of the Northern and Volta regions of Ghana deliver at home compared with the national average of 21% [5]. Delivering at home without the presence of a skilled birth attendant predisposes pregnant women to some life-threatening conditions. Facility-based delivery allows pregnant women to receive emergency care by skilled health professionals when complications arise which can help save the lives of both baby and mother [5]. Optimum antenatal care utilisation can pre-empt women the need for hospital delivery, thereby reducing possible pregnancy-related morbidities and mortalities.

Antenatal care (ANC) is the health care and education provided to pregnant women and adolescent girls by skilled health care professionals to ensure the best health conditions for the mother and the baby during pregnancy [1, 5]. Antenatal care utilisation is an important constituent of maternal health care, which reduces maternal and perinatal morbidity and

mortality both directly through identification and management of pregnancy-related complications, and indirectly through the identification of pregnant women and girls most likely to develop complications during labour and delivery, thus ensuring early referral to an appropriate health facility for further care [1]. Globally, while most women now attend at least one ANC visit (86%), only 62% attend four, with lower rates reported in sub-Saharan Africa and South Asia [6]. An analytical review of the recent WHO Global Health Observatory data repository shows that ANC coverage, between 2000 and 2017, was indirectly correlated with MMR worldwide [7, 8]. The evidence indicates that countries with poor ANC coverage are more likely to have high MMR [9-11]. For example, ANC utilisation in Australia is 94% with MMR of 6 per 100, 000, Finland has 99% ANC utilisation with MMR 3 per 100, 000 and France has 99% ANC utilisation with MMR 8 per 100000 live births. In comparison with sub-Saharan Africa, Nigeria has 49.1% ANC coverage with MMR of 917 per 100000, Cote d'Ivoire has 51.3% ANC coverage with MMR of 617 per 100,000, and Ghana has 89.3% ANC coverage with MMR 310 deaths per 100, 000 live births [7,8].

While ANC coverage remains high in Ghana, the coverage of at least four ANC visits remains lower at approximately 76% [12]. During pregnancy, the WHO recommends at least 4 ANC visits for antenatal care by a skilled health care professional for advice and monitoring of the health and well-being of both the mother and the developing foetus. A minimum of 4 visits constitutes receiving optimum ANC care. In this study, optimum ANC refers to a woman who made at least 4 visits for ANC during pregnancy. Geographically, despite the national rate of 76% for ANC care, disparities exist particularly among rural and poor socio-economic groups in Ghana on health care utilisation. To advance maternal and reproductive health outcomes for women throughout their life cycle and contribute to reducing current MMR, there is a need for more context evidence across Ghana in relation to an important outcome measure such as pregnant women's knowledge and optimum ANC during pregnancy. This study, therefore,

sought to assess women’s knowledge and its associated factors that influence optimum utilisation of ANC services (4+ visits) in a context that presents opportunity for health systems learning and policy recommendations as Ghana steps up efforts to ensure universal access to reproductive health care and for the attainment of the Sustainable Development Goals (SDGs) targets on maternal and reproductive health.

Conceptual framework

Deciding to utilise ANC services does not only depend on the individual’s preference but also the factors that facilitate the accessibility of the health facility (services) or the factors that impede the utilisation of ANC services [13]. WHO outlined that a woman’s ability to access antenatal care services is influenced by several factors that include distance or time taken to travel to a health facility, availability of ANC services, social and cultural factors that can serve as barriers to access, economic status, costs related with utilisation of services, and the ANC quality of services provided [14].

Several models have been developed over the last few decades to identify factors influencing healthcare service utilisation, and the most widely used is the Anderson Behavioral Health care model known as Behavioral Model of Health Services. This study was grounded based on the Andersons Behavioral Model and sought to identify factors that facilitate or impede the use of healthcare and to understand discrepancies in the utilisation of care [15]. This model was first developed to determine why families utilise health care, to identify equitable access to health services, and to support in formulating policies that will promote equitable access [16, 17]. According to the model, utilisation of health services is determined by three dynamics: (1) the predisposing factors, (2) enabling factors, and (3) need factor. Predisposing factors include individual characteristics and health beliefs; enabling factors are the logistic aspect of obtaining

care and need factors are the most immediate cause of healthcare utilisation such as the presence of disease condition and self-perceived functional or health status [16].

The current study sought to assess factors that influence (facilitate or impede) women's use of ANC services; community-based factors that impede or motivate the use of ANC, facility-based factors, and adequacy of ANC visits. Despite the importance of these individual determinants affecting ANC utilisation among women, the current study also sought to assess women's knowledge of ANC services and how that influence ANC use as evident in literature [18] recommended by WHO for pregnant women. The study identified the following (as shown in Figure 1) factors that influence ANC utilisation (either positive or negative) in the study setting among women.

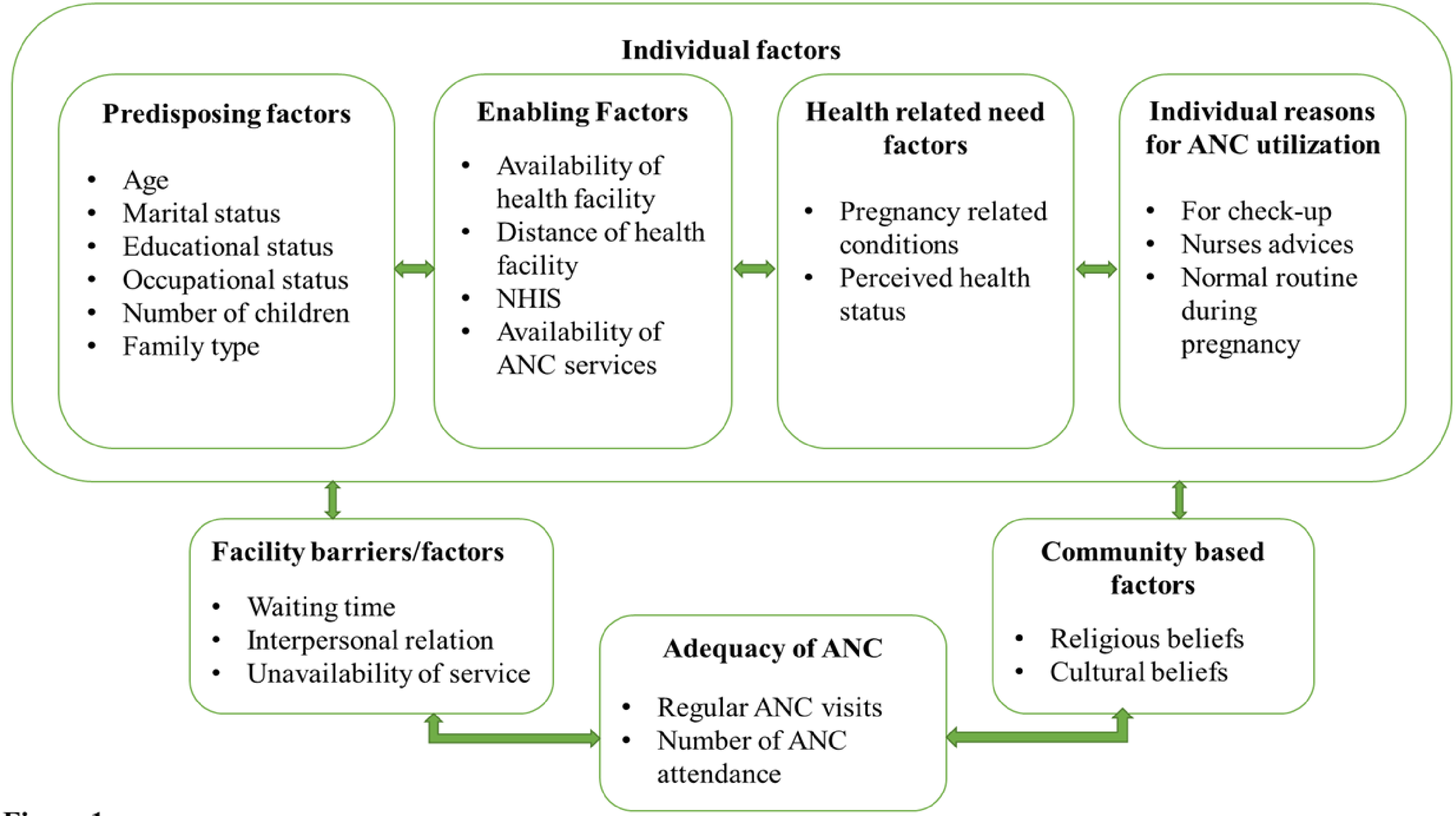

Figure 1:

- Conceptual framework for analyzing factors associated with ANC utilization among women in Northern Ghana
- Adapted from Andersen's behavioral model for health service use (Andersen, 1995)

134

Methods

Study Design

The study applied a cross-sectional analytical facility-based study design to recruit the participants. Given the design type, quantitative data was collected among eligible participants after applying the appropriate sampling methodology. This design was appropriate to enable the study team to elicit answers to key questions that underpin the study.

Study setting and population

The study was conducted at four health facilities in Bamboi, a sub-district jurisdiction under the Bole district in the Savanah region of Ghana. The health facilities were categorised as (A, B, C & D). Facility A had two midwives, four Enrolled Nurses (EN) and a community health nurse (CHN), facility B had two CHNs, facility C had a midwife and one CHN and facility D had male practicing midwifery. These health professionals provide maternal and child health services for mothers attending any of these facilities. The Bole District is one of the seven districts located in the newly created Savanah region of Ghana. The District is located at the extreme western part of the northern region of Ghana. The District is bordered to the North by Sawla / Tuna Kalba District, to the West by the Republic of Ivory Coast, to the East by West Gonja District and to the South by Wenchi and Kintampo Districts of Brong-Ahafo region. The District health services are divided into four sub-districts namely Bole, Tinga, Jama, and Bamboi. Each sub-district has an operational area served by a health facility. Bamboi is one of the sub-districts which is to the South of the Bole district. The sub-district, Bamboi where this study was conducted has a relatively small semi-urban area while a large proportion is rural. The Bamboi sub-district has one health center and three Community-Based Health Planning and Services (CHPS) zones serving over 26 communities within the sub-district. In Ghana, CHPS is the lowest level under the health level structures where health care is provided at the community level, closer to the people. The eligible study population were all reproductive-

aged women in the Bambi sub-district who attended the study health facilities in Bamboi sub-district for postnatal care during the time of the study.

Sampling Method and Sample Size Determination

Consecutive sampling was employed in recruiting participants. The average monthly attendance for the four facilities providing postnatal care services was estimated to be about 450. The total population for the four facilities for four months was 1800. The sample size for the study was determined by using the sample size calculation by Yamane [19]. The minimum required sample size was 310 and a 5% non-response rate was added to get a sample size of 340. Finally, 322 questionnaires were considered valid to be included for analysis.

Outcome variables

During data analysis, the outcome variables of interest were knowledge and utilisation of ANC services. The utilisation of ANC services was classified as adequate versus inadequate. The study assessed the overall adequacy of ANC visits by reviewing the mothers' ANC cards to determine the frequency of visits during the immediate past pregnancy. Antenatal care was classified as adequate if the woman had at least four or more antenatal visits during normal pregnancy and less than four visits were defined as inadequate. The knowledge of mothers on ANC services was classified as good knowledge and bad knowledge.

Explanatory variables

The explanatory variables were selected based on review of related literature and the modified version of the Anderson Healthcare Utilization model. Socio-demographic characteristics; Participants' age was categorised into <20yrs, 21-30yrs and 31-46 years. Marital status was categirised into not married, and married. Educational level of participants was categorised as

none, basic, secondary and tertiary. Occupation of participants was classified into farmer, trader, civil servant, and others. The religious status was categorised under Christianity and Islam. Mo, Gonja, Ewe, Akan, and Dagaaba were the ethnic groups of participants. The family type was the nuclear and extended family. The number of children was categorised into 1-2, 3-4 and 5+ children. Participants were either insured or not insured with the National Health Insurance Scheme (NHIS). Other variables of interest were distance to health facility (For CHPS, the distance was between a radius of 5 km (and for Health Centres between 8 and 16 km radius [20-22]. Women who trekked more than 5km and also 16km where considered far and those within the recommended limit were considered short), person who provides healthcare, person who makes healthcare decisions and means of transportation to the facility.

Data collection procedure

Data collection lasted for four months from October 2018 to January 2019. A self-administered semi-structured and pretested questionnaire was used to collect data from eligible participants. Four trained research assistants (nurses) were assisted to collect data in the four study health facilities. The research assistants were trained for 2 days and were supervised for a week by the principal investigator during data collection. The four facilities were visited every week during the postnatal days for questionnaire administration. Eligible participants were approached after receiving postnatal care and provided with further education on the study's aim and objectives. Eligible participants who were willing to participate were offered consent forms to endorse. Questionnaires were interviewer-administered by the study team. Completeness of questionnaires was checked daily after completing each questionnaire before participants were asked to leave. Where minor omissions were detected, immediate corrective measures were taken with participants to ensure validity and to prevent missing information.

Validity and reliability of the study instrument

The study instrument was developed after reviewing relevant related literature and also adopting Anderson health care behavioral model. Preceding data collection, the validity and reliability of the study instrument were assessed through various stages. To assess the content validity, a group of researchers and clinicians were made to review and refine the items in the questionnaire. This group comprised of two senior community midwives and two researchers in the field of maternal and child health. Further, the Pearson correlation coefficient was used to determine the test-retest reliability of the study with a sample of 20 participants. The instrument was administered to the same participants' after one week. The test-retest correlation coefficient for the instrument was 0.73 ($p < 0.001$), which is indicative of acceptable stability over time.

Questions asked on ANC knowledge

1. ANC can prevent complications in pregnancy?
2. Pregnant women may have problems without ANC?
3. Regular ANC medications can promote optimal growth of unborn child?
4. Health facility delivery is safer and better than home delivery?
5. Do you know the recommended place for delivery?
6. Do you know the recommended frequency and timing of ANC visits?
7. ANC is recommended regardless of complications?
8. Do you know items to prepare for before delivery?
9. Do have knowledge of family planning?
10. Do have knowledge of malaria prevention?

Figure 2: Key items used to assess women's knowledge of ANC services

Ethical consideration

Ethics approval for the study was obtained from the University of Health and Allied Sciences Research Ethical Committee (UHAS-REC A. 10[27] 17-18). Permission to conduct the study

was sought from the various facilities before the commencement of data collection. Both verbal and written consent was obtained from participants after agreeing to participate in the study. Participants were assured of anonymity, confidentially and privacy. Participants were made aware of the right to withdraw from the study at any particular point in time if they wished to do so.

Data Analysis

Data were entered into SPSS software, cleaned and validated to ensure completeness and quality before analysis. The cleaned data were exported to STATA version 14.0 for analysis. Descriptive statistics were applied to represent frequencies and percentages. The overall level of knowledge on antenatal care was measured by scoring responses that measured participants' knowledge (figure 2) using the following descriptions; a score of 1 was assigned if the participant had knowledge and 0 if they had no knowledge. Total score and mean scores were computed and a score below the mean was considered poor knowledge whiles a mean score or above was considered to be a good knowledge. Ordinary least square logistic regression analysis, computing odds ratio, was used to determine the association between dependent variables (ANC service utilisation and knowledge of ANC) and socio-demographic characteristics. Statistical significance was determined at 95% confidence level.

Results

Participants' socio-demographic characteristics

Slightly more than half, 177 (55.0%) of participants were aged 21-30 years while about half, 167 (51.9%) were not married. A quarter of the women, 111 (34.5%) had basic and secondary education, and more than half, 188 (58.4%) engaged in trading. About 144 (44.7%) belonged to the Dagaaba ethnic group. The majority, 288 (89.4%) had the nuclear family type and over two-thirds had 1-2 children. The majority, 277 (86.1%) had to walk to access healthcare. Most, 210 (65.2%) made their own decision regarding their healthcare needs (Table 1).

Table 1: Participants' socio-demographic characteristics (N=322)

Variable	Frequency (f)	Percentage (%)
**Age**		
≤ 20	44	13.6
21-30	177	55.0
31-40	101	31.4
**Marital Status**		
Married	133	41.3
Not married	189	58.7
**Educational status**		
None	33	10.2
Basic	111	34.5
Secondary	111	34.5
Tertiary	67	6.8
**Employment**		
Employed	122	37.9
Unemployed	189	58.7
Student	11	3.4
**Occupation**		
Farmer	56	17.4
Trader	188	58.4
Civil servant	56	17.4
Others	22	6.8
**Religion**		
Christianity	255	79.2
Islam	67	20.8
**Ethnicity**		
Mo	100	31.1
Gonja	33	10.3
Ewe	11	3.4
Akan	34	10.5
Dagaaba	144	44.7
**Family type**		
Nuclear	288	89.4
Extended	34	10.6
**Number of children**		

1-2 children	245	76.1
3-4 children	55	17.1
5+ children	22	6.8
**Number of ANC visits**		
<4	100	31.0
4+	222	69.0
**Health insurance**		
Insured	245	76.1
Not insured	77	23.9
**Distance to health facility**		
Short	220	68.3
Far	102	31.7
**Transportation to ANC**		
Motorcycle	23	7.1
Foot	277	86.1
Others	22	6.8
**Person who provides for healthcare**		
Self	90	27.9
Partner	166	51.6
Parents	66	20.5
**Person who makes healthcare decision**		
Self	210	65.2
Partner	101	31.4
Parents	11	3.4

Factors associated with the utilisation of antenatal care services

In table 2, the logistic regression analysis showed that women who were aged 21-30 years were 4 times more likely to utilise ANC services four or more times compared to those aged 20 years and below [OR=4.36 (95%CI: 2.16-8.80), p<0.001] and those who were not married were 70% less likely to utilise ANC services at least four times compared to those who were married [OR=0.30 (95%CI: 0.18 – 0.50), p<0.001]. Women who had tertiary education were 10 times more likely to utilise ANC services four or more times than those without education [OR=10.18 (95%CI: 3.86-26.87) p<0.001]. Participants who were insured with the NHIS were 3 times more likely to utilise the ANC compared to those who were not-insured [OR=3.42 (95%CI: 1.72-6.82), p<0.001]. Regarding distance to health facilities, women who reported tha they

stayed far from health facilities were 80% less likely to utilise ANC compared to women who stayed closer to health facilities [OR=0.20 (95%CI: 0.12-0.34), p<0.001].

After adjusting for other explanatory variables women who were not married were 84% less likely to utilise ANC services four or more times [AOR=0.16 (95%CI: 0.01 – 0.75), p=0.03]. Women who had tertiary education were 10 times more likely to utilise ANC services four or more times than those without education [AOR=5.26 (95%CI: 2.15-17.28) p<0.001]. Women who stayed far from the health facilities were 65% less likely to attend ANC for at least four times [AOR=0.35 (95%CI: 0.02-0.13), P=0.001]

Table 2: Logistic regression results showing optimum utilization of ANC services

**Variable**	**Usage of ANC Services**		**COR [95% CI]**	**p-value**	**AOR [95% CI]**	**p-value**
	<4=**100 n(%)**	**4+=222 n(%)**				
**Age**						
≤20	22 (22.0)	22 (9.9)	**Ref**		**Ref**	
21-30	33 (33.0)	144 (64.9)	4.36[2.16-8.80]	<0.001	2.01[0.32-5.70]	0.043
31-46	45 (45.0)	56 (25.2)	1.24[0.61-2.53]	0.546	0.24[2.16-8.80]	0.067
**Marital Status**						
Married	33 (33.0)	100 (45.0)	**Ref**			
Not married	67 (67.0)	122 (55.0)	0.30 [0.18 – 0.50]	<0.001	0.16 [0.01 – 0.75]	0.030
**Educational status**						
None	22 (22.0)	11 (5.0)	**Ref**		**Ref**	
Basic	22 (22.0)	89 (40.1)	8.09 [3.42 -19.14]	<0.001	3.19 [2.35-16.75]	0.056
Secondary	45 (45.0)	66 (29.7)	2.93 [1.29- 6.64]	0.010	1.29 [1.29- 6.64]	0.045
Tertiary	11 (11.0)	56 (25.2)	10.18[3.86-26.87]	<0.001	5.26[2.15-17.28]	<0.001
**Occupation**						
Farmer	33 (33.0)	23 (10.4)	**Ref**			
Trader	45 (45.0)	143 (64.4)	4.55[2.40- 8.55]	<0.001		
Civil servant	11 (11.0)	45 (20.3)	5.87[2.51-13.69]	<0.001		
Others	11 (11.0)	11 (4.9)	1.43[0.53-3.86]	0.475		
**Religion**						
Christianity	44 (44.0)	211 (95.1)	**Ref**		**Ref**	
Islam	56 (56.0)	11 (4.9)	0.04[0.02-0.08]	<0.001	0.017[0.01-0.29]	0.088
**Family type**						
Nuclear	88 (88.0)	200 (90.1)	**Ref**			
Extended	12 (12.0)	22 (9.9)	0.81[0.38-1.70]	0.573		
**Health insurance**						
Not Insured	11 (11.0)	66 (29.7)	**Ref**			

Insured	89 (89.0)	156 (70.3)	3.42[1.72-6.82]	<0.001		
**Distance to health facility**						
Short	44 (44.0)	176 (79.3)	**Ref**		**Ref**	
Far	56 (56.0)	46 (20.7)	0.20[0.12-0.34]	<0.001	0.35[0.02-0.13]	0.001
**Provider for healthcare**						
Self	34 (34.0)	56 (25.2)	**Ref**			
Partner	33 (33.0)	133 (59.9)	2.44 [1.38-4.33]	0.002		
Parents	33 (33.0)	33 (14.9)	0.60 [0.32-1.16]	0.129		

COR=Crude Odds ratio, **AOR**=Adjusted Odds ratio, adjusted for age, marital status, educational level, religion, and distance to health facility.

Participants Knowledge of Antenatal Care Services

Figure 3 shows that 79.2% of the participants had good level of knowledge regarding of antenatal care while 20.8% had poor knowledge regarding antenatal care.

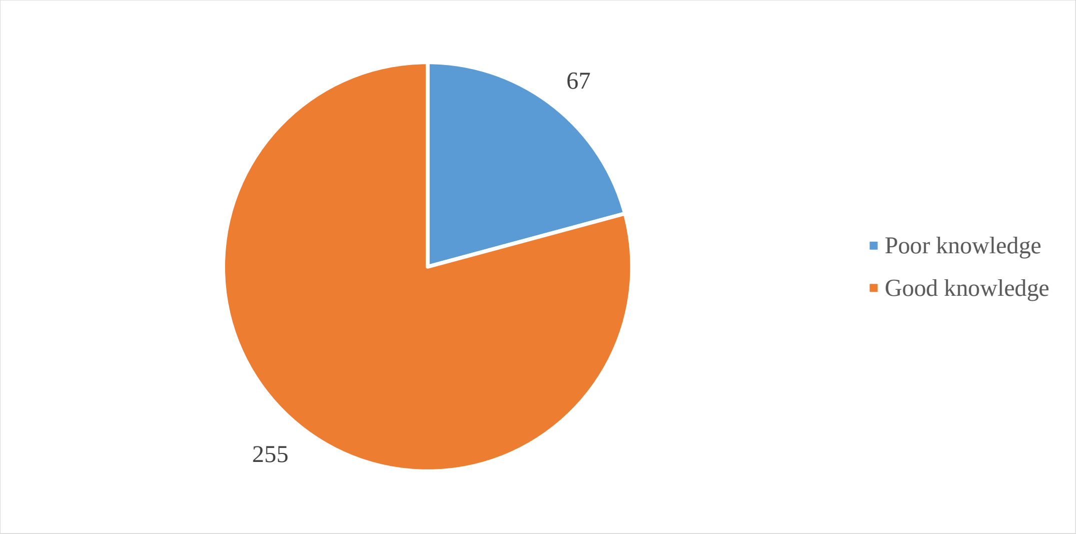

Figure 3: Overall level of knowledge regarding ANC

Association between Socio-demographics characteristics of participants and knowledge of antenatal care

Not being married and divorced women were 75% and 91% respectively less likely to have good knowledge regarding ANC [OR=0.25, (95%CI: 0.13 – 0.51), p<0.001; OR=0.09, (95%CI: 0.03 – 0.26), p<0.001]. Women who attained basic school education were 4.47 times more likely to have good knowledge of ANC compared to those with no education [OR=4.47, (95%CI: 1.75 – 11.39), p=0.002]. Respondents who belonged to the Islamic religion were 85% less likely to have good knowledge on ANC compared to respondents who were Christians [OR=0.15, (95%CI: 0.08 – 0.27), p<0.001]. Regarding family status and its association with women use of ANC services, women who lived in an extended family system were 99% less likely to have good knowledge on ANC compared to those who lived in a nuclear family environment [OR=0.01, (95%CI: 0.00 – 0.03), p< 0.001] (see table 3).

Multi-variable logistic regression analysis revealed that respondents aged >30 years were 98% less likely to have adequate knowledge on antenatal care compared to those aged 20 years and below [AOR=0.02, (95%CI: 0.00 – 0.45), p=0.013]. Not married women were 99% less likely to have adequate knowledge on antenatal care compared to married women [AOR=0.01, (95%CI: 0.00 – 0.28), p<0.001]. Women who had 5+ children were 98% less likely to have adequate knowledge on antenatal care compared to those with 2 children or less [AOR=0.02, (95%CI: 0.00 – 0.43), p=0.012].

Table 3; Logistic regression results of factors associated with women's knowledge of ANC services

Variable	Knowledge of ANC Services		COR [95% CI]	p-value	AOR[95% CI]	p-value
	**Poor n=67 (%)**	**Good n=255 (%)**				
**Age (years)**						
≤ 20	0 (0.00)	44 (17.2)	**Ref**		**Ref**	
21-30	33 (49.3)	144 (56.5)	0.05 [0.00 – 0.81]	0.035	0.10 [0.01 – 1.76]	0.115
>30	34 (50.7)	67 (26.3)	0.02 [0.00 – 0.36]	0.008	0.02 [0.00 – 0.45]	0.013
**Marital Status**						
Married	11 (16.4)	122 (47.8)	**Ref**		**Ref**	
Not married	56 (83.6)	1323(52.2)	0.25[0.13 – 0.51]	<0.001	0.01 [0.00 – 0.28]	<0.001
**Educational status**						
None	11 (16.4)	22 (8.6)	**Ref**			
Basic	11 (16.4)	100 (39.2)	4.47[1.75 –11.39]	0.002		
Secondary	34 (50.8)	77 (30.2)	1.15[0.51 – 2.60]	0.740		
Tertiary	11 (16.4)	56 (22.0)	2.51[0.96 – 6.50]	0.058		
**Occupation**						
Farmer	11 (16.4)	45 (17.6)	**Ref**			
Trader	45 (67.2)	43 (56.1)	0.80[0.38 - 1.65]	0.541		
Civil servant	11 (16.4)	45 (17.7)	0.10 [0.40 – 2.50]	0.001		
Others	0 (0.00)	22 (8.6)	11.37[0.64–21.83]	0.098		
**Religion**						
Christianity	33 (49.3)	222 (87.1)	**Ref**		**Ref**	
Islam	34 (50.7)	33 (12.9)	0.15[0.08 – 0.27]	<0.001	0.23 [0.08 – 0.71]	0.010
**Ethnicity**						
Mo	0 (0.00)	100 (39.2)	**Ref**			
Gonja	22 (32.8)	11 (4.3)	0.01[0.00 – 0.04]	<0.001		
Ewe	0 (0.00)	11 (4.3)	0.11[0.00 – 6.04]	0.280		
Akan	12 (17.9)	22 (8.7)	0.01[0.00 – 0.16]	0.001		
Dagaaba	33 (49.3)	111 (43.5)	0.02[0.00 – 0.27]	0.004		

**Family type**						
Nuclear	33 (49.2)	255 (100)	**Ref**		**Ref**	
Extended	34 (50.7)	0 (0.00)	0.01 [0.00 – 0.03]	<0.001	0.01 [0.00 – 0.02]	<0.001
**Number of children**						
≤ 2 children	45 (67.1)	200 (78.4)	**Ref**		**Ref**	
3-4 children	11 (16.4)	44 (17.3)	0.89 [0.42 – 1.81]	0.725	0.02 [0.00 – 2.82]	0.116
5+ children	11 (16.4)	11 (4.3)	0.23 [0.09 – 0.55]	0.001	0.02 [0.00 – 0.43]	0.012

COR=Crude Odds ratio, **AOR**=Adjusted Odds ratio, Adjusted for age, marital status, religion, family type, number of children.

Community and individual challenges affecting antenatal utilization

Long-distance to the health facility (31.1%) and partner's perception of the importance of ANC were major barriers to the effective utilisation of ANC services by the women. Also, about 27.6% indicated not having enough money to attend antenatal clinic and cultural beliefs (7.9%) as some of the challenges. This is shown in (Figure 4).

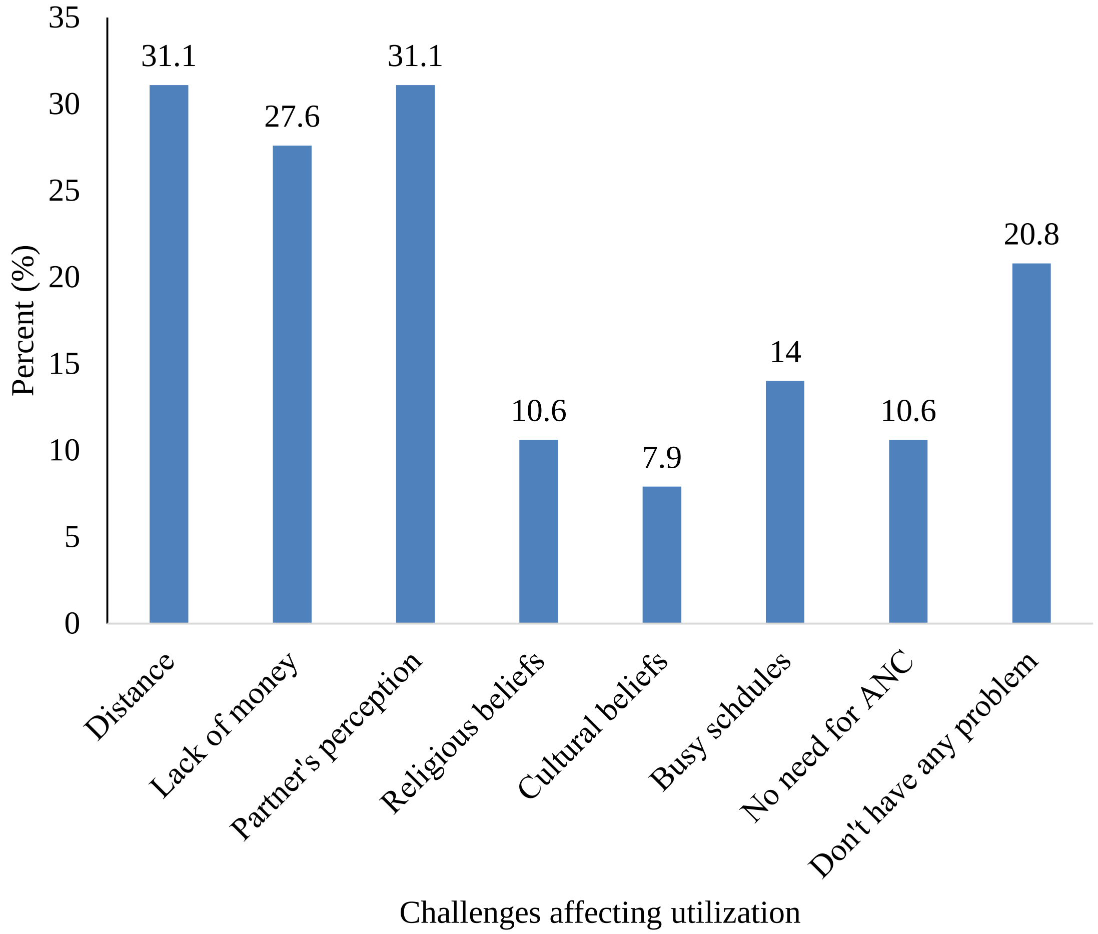

Figure 4: Challenges affecting utilisation of ANC

Facility related barriers to ANC attendance

Figure 5 showed that most, 38.2% of the participants were faced with long waiting times in health facilities where they assessed care, followed by 28% who were scolded by staff and 24.5% who had inconvenient service hours.

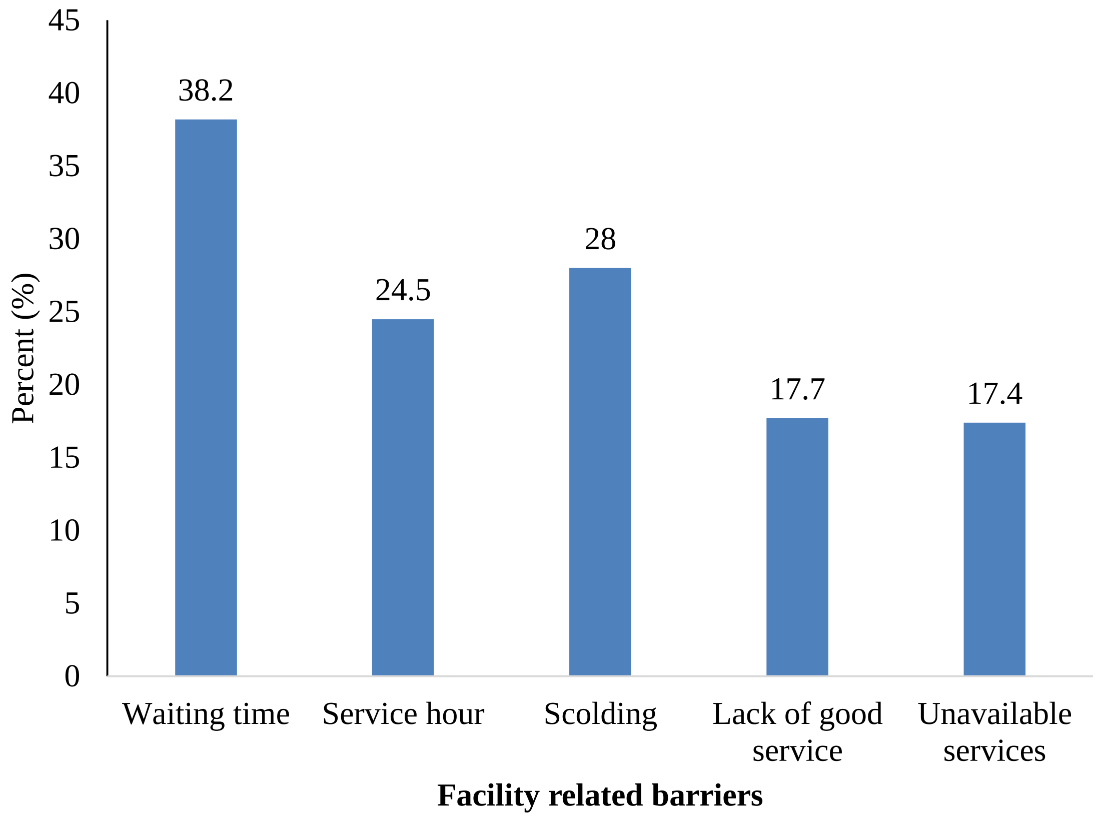

Figure 5: Facility related barriers to ANC utilization

Reasons for ANC Attendance

Figure 6 also showed that majority, 76.1% of the respondents went for antennal services for check-ups, while in 24.2% of them, it was because of normal routine check-ups during pregnancy. Also, about one-tenth, 10.6% went for antenatal care services as a result of health workers' advice.

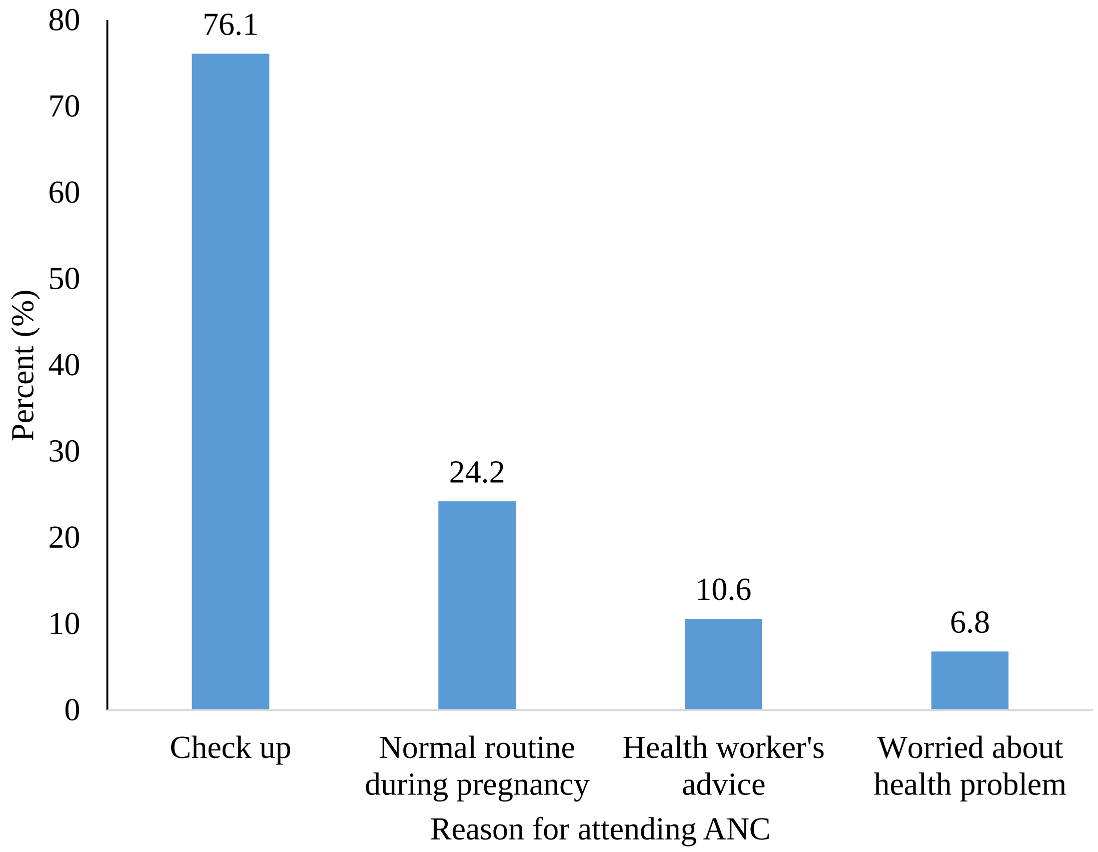

Figure 6: Reason for attending ANC

Discussion

This study applied an integrated conceptual framework to identify individual and facility based factors that influence optimum utilisation of ANC services among mothers in rural areas. Adequacy of ANC services was also assessed. And for a pregnant woman to attain optimum

utilisation or adequacy of ANC services, the World Health Organization recommends four or more ANC visits for women who do not experience any complications throughout their pregnancies [23]. The current study finding revealed that 69.0% of the women attended ANC at least four or more times. This study corroborates results in Ethiopia [24] but higher than some earlier studies in Rwanda [3], Ethiopia [25], and Kenya [26]. Though this study result is high as compared to other countries, it is low when compared to findings in previous studies in Ghana [27] Nepal [28] and Nigeria [9]. The divergence could be due to socio-cultural, economic, and awareness differences among the study populations as purported by other studies [25, 29]. The present finding of 69% is below the national coverage of at least four ANC visits of 75.9% [12]. This difference may be due to the lack of awareness of optimal ANC visits. Also, the differences could have been caused by other potential inhibiting factors such as distance to health facilities and socio-economic factors. Therefore, there is the need to intensify interventions implemented by the Ghana Health Service over the years such as Social and Behaviour Change Communication (SBCC) targeting reproductive-age women on optimum ANC utilisation and the importance of early initiation and regular ANC visits during pregnancy. In addition, current national policy initiatives to extend the Community-based Health Planning Services (CHPS) concept to cover the entire country in order to meet the reproductive health needs of many women, particularly in rural areas of Ghana, remain a laudable intervention in Ghana [12]. CHPS offer opportunity to bridge distance barriers and provide reproductive-age women better chances of obtaining relevant and useful information on ANC and its importance throughout pregnancy as well as serve as an effective Primary Health Care (PHC) strategy to improve access to maternal health and capacity building for improved client-service provider relationship.

Participants' educational level was found to be a strong predictor of optimum utilisation ANC services for four or more times. Participants who had basic, secondary, and tertiary education were more likely to use ANC services compared to those who had no education. This finding is not counter-intuitive because previous studies in developing countries have reported that the educational level of women increases the odds of utilising ANC services for four or more times [9, 23, 30-33]. Probably the educated women are more inclined to issues of their health (identifying danger signs) thereby placing more priority on their health and that of the unborn child. The major cause of health inequality in ANC coverage has been strongly associated with women's poor/lack of education [34]. This highlights the need for a multisectoral approach, with the health sector working jointly with other sectors such as the educational sector to promote female education to the highest level.

Participants who were not married or divorced were less likely to attend ANC for at least four or more times relative to their counterparts who were married. In a Ghanaian community, women who are not married are expected to remain virtuous until marriage [35] in order to prevent public ridicule, so women who get pregnant without a husband could avoid ANC visits [27] to prevent public ridicule. On the other hand, women who are not married and those with low socio-economic status are likely to lack the financial means (for travel cost and medicines) and may be worse affected when it comes to meeting the number of recommended ANC care services during pregnancy. The free fee delivery care policy introduced by the Government of Ghana through the NHIS continues to play a significant role in enhancing lower socio-economic groups' access and use of maternity care services, including ANC care. However, bottlenecks such as poor re-imbursements issues under the NHIS impact negatively on pro-poor women's access to ANC care and other general health care needs. Health system improvements that enhance the efficiency of

the NHIS and broaden the benefits package for women reproductive health care services can go a long way to improve long term health gains for maternal health outcomes in Ghana.

Women who were insured with the NHIS were three times more likely to utilise ANC services for four or more times compared to those who were not insured. This finding is consistent with other studies in Ghana [27, 36-38]. While the number of visits a pregnant woman makes for ANC is key to attaining optimum ANC, the quality of ANC services is also relevant. Ayanore and colleagues in a study on focused ANC among women found that ANC quality predict the adequacy of ANC services relative to the frequency of visits made by a woman [39]. Ghana's implementation of a free delivery policy in July 2008 for pregnant women has played a major role in improving maternal and child health outcomes in Ghana. The NHIS which allows pregnant women access to benefit from this free fee policy has also assisted to improve health care access and utilisation for all age groups. This study findings show that being insured under the NHIS can enhance women's attainment of optimum ANC. Thus, the implementation of the free maternal health care policy under the NHIS instituted by the government of Ghana is key to achieving increased utilisation of ANC services for at least four times during every normal pregnancy [40].

Despite the potential benefit of the NHIS, registration challenges for enrolments often resulting in long queues making it difficult for pregnant women to register on time is documented [27, 41]. There is evidence in Ghana that demonstrates that poor NHIS client registration is mostly limited to the district capital because of the biometric system used; where people including pregnant women will have to trek long distances to register [27, 41]. The NHIS system has over the last few years introduced mobile phone technology to improve client registrations and address current challenges in enrolments under the NHIS. However, non-educated, pro-poor, and rural women are

likely not to benefit from high technological applications on NHIS registrations. NHIS reforms must prioritize how to improve client's engagements for the rural poor, and enhance vulnerable groups' chances to obtain NHIS. New policy reforms that will enhance access for NHIS enrolments in distant and rural areas are recommended in addition to new technological strategies being currently implemented by the NHIS

Women who trek far distances to the health facility for ANC services were 80% less likely to utilise ANC services for four or more times compared to those with trek shorter distances. The current study finding corroborates studies in Uganda and Pakistan where incomplete ANC attendance was attributed to long distance to the health facility [42 43]. Establishment of health facilities within catchment areas in rural communities, employing more qualified health personnel to deliver healthcare to the women in the communities, and access to good roads among others by the government of Ghana is significant in ensuring increased ANC utilisation.

Women's knowledge of ANC is crucial in the utilisation of ANC services during pregnancy. This study revealed that 79.2% of the women had good knowledge of ANC services. The determinants of women having knowledge of ANC services included; woman's age, woman's educational status, husband's educational status, religion, ethnicity, and the number of children. Our study finding is consistent with a study conducted in the southwest of Nigeria [44] but disagrees with a study conducted in Mozambique where the researchers found women's knowledge of ANC services not to have significant importance in their utilisation of ANC services [45]. It is established that women with good knowledge of ANC services have a better understanding and acceptance of the services provided during ANC [46]. Women's chances to have a good knowledge of ANC care services is thus a catalyst for improving ANC care-seeking. National-level policies that ensure focused ANC services, become an integral part of ANC and other maternal care

services are fundamental to supporting improve knowledge, address myths and remove other health care seeking challenges women encounter when using ANC care services.

On the aspect of facility-based factors that hinders ANC utilisation, the study found that waiting for a long time in the facilities to receive care, was the major implicating factor for facility-based barriers to ANC utilisation. This finding is congruent with a study conducted in Mozambique where the women identified long waiting times as a barrier to ANC utilisation [45]. The long waiting time identified by participants as a barrier to ANC service utilisation could be due to the shortage of qualified healthcare professionals needed to provide care to over 26 communities in the study setting. Pregnant women might find it so distressing waiting for long hours without receiving care and forgoing certain duties which might have financial implications on them and this may demotivate women for subsequent ANC service utilisation. Gong et al., [45] further support our assertion by illustrating that long waiting times have direct and indirect cost implications for clients seeking care and that attending to clients on schedule mitigates cost and also improves women accessing ANC services.

Limitations of the study

First, recall bias could have limited the validity of the data collected as some participants might have forgotten about past events involving ANC services. To minimize recall bias, the study assessed the immediate past ANC records and we crosschecked self-reported information against the ANC attendance book for the validity of the information. Secondly, the study design was cross-sectional, hence results should be interpreted with caution since this study did not provide an opportunity to assess cause-effect relationship. Also, several individuals, households, and health system factors influence optimum ANC services, which may have been missed and not included

in this study. This study was facility-based, hence the opportunity to miss out on eligible participants at the population level is acknowledged. Notwithstanding, this study provides useful information on women's knowledge and factors that influence optimum antenatal care utilisation in rural settings in Ghana.

Conclusion

Although the majority of the women had good knowledge of ANC services, a significant number did not complete the recommended number of ANC visits for at least four times during a normal pregnancy. The creation of awareness of adequate ANC attendance in rural settings should be emphasized by frontline health workers especially nurses and midwives working in these communities. Not insured with NHIS lowered the chances for women to have received optimum ANC services. Health system reforms that address NHIS access challenges are essential to support women attain optimum ANC and supportive focused ANC care in Ghana.

Further research is recommended to determine sociocultural, traditional, religious and other related practices at the community level on the utilisation of antenatal care services.

Abreviations

ANC: Antenatal care

CHPS: Community-based Health Planning Services

EN: Enrolled Nurses

GMHS: Ghana Maternal Health Survey

MMR: Maternal Mortality Ratio

NHIS: National Health Insurance scheme

SBCC: Social and Behaviour Change Communication

WHO: World Health Organisation

Conflict of interest

Authors declared no competing interest.

Data Availability Statement:

All relevant data are within the manuscript and its Supporting Information files.

Funding

The study received no external funding

Acknowledgments

Our profound gratitude goes to the health facilities for their support and co-operation during data collection. We are also grateful to the women who took time off their busy schedules to participate in the study.

Author Contributions:

Conceptualization: Agani Afaya, Thomas B. Azongo, Veronica Millicent Dzomeku, Richard Adongo Afaya, Solomon Mohammed Salia, David Adadem, mmanuel Opoku Asiedu

Data curation: Agani Afaya, Richard Adongo Afaya, Solomon Mohammed Salia, David Adadem, Emmanuel Opoku Asiedu

Formal analysis: Agani Afaya, Peter Adatara, Robert Kaba, Martin Amogre Ayanore, Confidence Alorse Atakro

Funding acquisition: No funding

Investigation: Agani Afaya Thomas B. Azongo, Veronica Millicent Dzomeku, Hyeonkyeong Lee, Richard Adongo Afaya, [1]Solomon Mohammed Salia, Peter Adatara, Robert Kaba Alhassan, Martin Ayanoore, Confidence alorse Atakro, Paul Amuna

Methodology: Agani Afaya Thomas B. Azongo, Veronica Millicent Dzomeku, Hyeonkyeong Lee, Richard Adongo Afaya, Solomon Mohammed Salia, Peter Adatara, Robert Kaba Alhassan, Marin Amogre Ayanore, Confidence alorse Atakro, Paul Amuna

Project administration: Agani Afaya Thomas B. Azongo, Veronica Millicent Dzomeku, Hyeonkyeong Lee, Richard Adongo Afaya, [1]Solomon Mohammed Salia, Peter Adatara, Robert Kaba, Martin Amogre Ayanore, Confidence Alorse Atakro, Paul Amuna

Resources: Agani Afaya, Richard Adongo Afaya

Software: Agani Afaya

Supervision: Agani Afaya, Thomas B. Azongo, Veronica Millicent Dzomeku, Hyeonkyeong Lee, Peter Adatara, Robert Kaba, Martin Amogre Ayanore, Paul Amuna

Validation: Agani Afaya, Thomas B. Azongo, Veronica Millicent Dzomeku, Hyeonkyeong Lee, Peter Adatara, Robert Kaba, Martin Amogre Ayanore, Abigail Kusi-Amponsah Diji, Paul Amuna

Visualization: Agani Afaya, Thomas B. Azongo, Veronica Millicent Dzomeku, Hyeonkyeong Lee, Richard Adongo Afaya, [1]Solomon Mohammed Salia, Peter Adatara, Robert Kaba, Martin Amogre Ayanore, Abigail Kusi-Amponsah Diji, Confidence Alorse Atakro, Paul Amuna

Writing – original draft: Agani Afaya, Richard Adongo Afaya

Writing – review & editing: Agani Afaya, Thomas B. Azongo, Veronica Millicent Dzomeku, Hyeonkyeong Lee, Richard Adongo Afaya, Solomon Mohammed Salia, Peter Adatara, Robert Kaba, Martin Amogre Ayanore, Abigail Kusi-Amponsah Diji, Confidence Alorse Atakro, Paul Amuna

Reference

1. World Health Organization. WHO recommendations on antenatal care for a positive pregnancy experience. World Health Organization; 2016.
2. World Health Organization. Trends in maternal mortality: 1990-2015: estimates from WHO, UNICEF, UNFPA, World Bank Group and the United Nations Population Division: executive summary. World Health Organization; 2015.
3. Rurangirwa AA, Mogren I, Nyirazinyoye L, Ntaganira J, Krantz G. Determinants of poor utilization of antenatal care services among recently delivered women in Rwanda; a population based study. BMC pregnancy and childbirth. 2017;17(1):142.
4. Gunawardena N, Bishwajit G, Yaya S. Facility-Based maternal death in Western Africa: a systematic review. Frontiers in public health. 2018 Feb 26;6:48.
5. Ghana Statistical Service (GSS), Ghana Health Service (GHS), and ICF. 2017 Ghana Maternal Health Survey Research Policy Briefs. Accra, Ghana: GSS, GHS, and ICF. 2019
6. UNICEF. Antenatal care. 2018. Available from; https://data.unicef.org/topic/maternal-health/antenatal-care/ (accessed March 7, 2019).
7. WHO: Global Health Observatory data repository**:** Antenatal care coverage Data by country Available from; http://apps.who.int/gho/data/node.main.ANTENATALCARECOVERAGE4 (accessed 17 January 2020)
8. WHO: Global Health Observatory (GHO) data; Country specific: Available from; https://www.who.int/gho/countries/en/

9. Fagbamigbe AF, Idemudia ES. Barriers to antenatal care use in Nigeria: evidences from non-users and implications for maternal health programming. BMC pregnancy and childbirth. 2015 Dec 1;15(1):95.

10. Bustreo F, Say L, Koblinsky M, Pullum TW, Temmerman M, Pablos-Mendez A. Ending preventable maternal deaths: the time is now. Lancet, Glob Heal. 2013;1:e176–7.

11. Ashir GM, Doctor HV, Afenyadu GY. Performance based financing and uptake of maternal and child health services in yobe sate, northern Nigeria. Glob J Heal Sci. 2013;5:34–41.

12. Ghana Health Service, Family Health Division, Annual Report . 2016. https://www.ghanahealthservice.org/downloads/FHD_2016_ANNUAL_REPORT_Final_June%2019.2017%20nat%20final.pdf

13. Hijazi HH, Alyahya MS, Sindiani AM, Saqan RS, Okour AM. Determinants of antenatal care attendance among women residing in highly disadvantaged communities in northern Jordan: a cross-sectional study. Reproductive health. 2018 Dec;15(1):106.

14. World Health Organization. Antenatal care in developing countries: promises, achievements and missed opportunities. 2003. Available from; http://apps.who.int/iris/bitstream/handle/10665/42784/9241590947.pdf?sequence=1

15. Babitsch B, Gohl D, Von Lengerke T. Re-revisiting Andersen’s Behavioral Model of Health Services Use: a systematic review of studies from 1998–2011. GMS Psycho-Social-Medicine. 2012;9.

16. Andersen RM. Revisiting the behavioural model and access to medical care: does it matter? J Health Soc Behav. 1995;3(6):1–10.

17. Andersen R, Newman JF. Societal and Individual Determinants of Medical Care Utilization in the United States. Milbank Q. 2005;83(4):1–28.
18. Ogunba BO, Abiodun OB. Knowledge and attitude of women and its influence on antenatal care attendance in Southwestern Nigeria. J Nutr Health Sci. 2017;4(2):207.
19. Yamane T. Statistics, An Introductory Analysis, 2nd Ed., 1967. New York: Harper and Row.
20. Ministry of Health. National Community-Based Health Planning and Services Policy. Ghana, Accra. 2006
21. Ghana Health Service (GHS). The Community-Based Health Planning and Services initiative: Concepts and plans for implementation. Accra, Ghana. 2002.
22. Ministry of Environment Science and Technology (MEST). Zoning guidelines and planning standards. 2011.
23. World Health Organization, United Nations Population Fund, United Nations International Children's Emergency Fund, The World Bank. Pregnancy, childbirth, postpartum and newborn care: a guide for essential practice (3rd edition). World Health Organization. Available from: http://www.who.int/maternal_child_adolescent/documents/924159084x/en/
24. Birmeta K, Dibaba Y, Woldeyohannes D. Determinants of maternal health care utilization in Holeta town, central Ethiopia. BMC Health Serv Res. 2013;13(1):256.
25. Ayalew TW, Nigatu AM. Focused antenatal care utilization and associated factors in Debre Tabor Town, northwest Ethiopia, 2017. BMC research notes. 2018 Dec 1;11(1):819.

26. Gitonga E. Determinants of focused antenatal care uptake among women in tharaka nithi county, Kenya. Adv Public Health. 2017;2017:4.
27. Sakeah E, Okawa S, Rexford Oduro A, Shibanuma A, Ansah E, Kikuchi K, Gyapong M, Owusu-Agyei S, Williams J, Debpuur C, Yeji F. Determinants of attending antenatal care at least four times in rural Ghana: analysis of a cross-sectional survey. Global health action. 2017;10(1):1291879.
28. Tuladhar H, Dhakal N. Impact of antenatal care on maternal and perinatal outcome: a study at Nepal Medical College Teaching Hospital. Nepal J Obstet Gynaecol. 2011;6(2):37–43.
29. Manyeh AK, Amu A, Williams J, Gyapong M. Factors associated with the timing of antenatal clinic attendance among first-time mothers in rural southern Ghana. BMC Pregnancy and Childbirth. 2020;20(1):47.
30. Nebeb GT, Salgedo WB, Alemayehu YK. Antenatal care utilization in Debre Tabor, North West Ethiopia. Gynecol Obstet. 2015;5:339. https ://doi.org/10.4172/2161-0932.10003 39.
31. Kalule-Sabiti I, Amoateng AY, Ngake M. The effect of socio-demographic factors on the utilization of maternal health care services in Uganda. Afr Popul Stud. 2014;28(1):515–25.
32. Tiruaynet K, Muchie KF. Determinants of utilization of antenatal care services in Benishangul Gumuz Region, Western Ethiopia: a study based on demographic and health survey. BMC pregnancy and childbirth. 2019;19(1):115.
33. Okedo-Alex IN, Akamike IC, Ezeanosike OB, Uneke CJ. Determinants of antenatal care utilisation in sub-Saharan Africa: a systematic review. BMJ open. 2019;9(10):e031890.

34. Ayamolowo SJ, Irinoye O, Oladoyin MA. Job satisfaction and work environment of primary health care nurses in Ekiti state, Nigeria: an exploratory study. *Internatinal Journal of Caring Sciences*. 2013;6:531–42.

35. Anarfi KJ. To Change or not to change: obstacles and resistance to sexual behavioural change among the youth in Ghana in the era of AIDS. Res Rev. 2003; 19:27–45.

36. Nketiah-Amponsah E, Senadza B, Arthur E. Determinants of utilization of antenatal care services in developing countries: recent evidence from Ghana. Afr J Econ Manag Stud. 2013;4:58–73.

37. Amoakoh-Coleman M, Ansah EK, Agyepong IA, Grobbee DE, Kayode GA, Klipstein-Grobusch K. Predictors of skilled attendance at delivery among antenatal clinic attendants in Ghana: a cross-sectional study of population data. BMJ open. 2015;5(5):e007810. e007810.

38. Arthur E. Wealth and antenatal care use: implications for maternal health care utilisation in Ghana. Health Econ Rev. 2012;2:14.

39. Ayanore MA, Pavlova M, & Groot, W. Focused maternity care in Ghana: results of a cluster analysis. BMC Health Serv Res 16, 395 (2016). https://doi.org/10.1186/s12913-016-1654-5.

40. Afaya A, Dzomeku VM, Baku EA, Afaya RA, Ofori M, Agyeibi S, Boateng F, Gamor RO, Gyasi-Kwofie E, Nyaledzigbor PP. Women's experiences of midwifery care immediately before and after caesarean section deliveries at a public Hospital in the Western Region of Ghana. BMC Pregnancy and Childbirth. 2020;20(1):1-9.

41. Duku S, Alhassan RK, Nketiah-Amponsa E. Barriers to Uptake and Renewal of Health Insurance Membership: Experiences from Ghana's National Health Insurance Scheme (NHIS). 2016

42. Kawungezi PC, AkiiBua D, Aleni C, Chitayi M, Niwaha A, Kazibwe A, Sunya E, Mumbere EW, Mutesi C, Tukei C, Kasangaki A. Attendance and utilization of antenatal care (ANC) services: multi-center study in upcountry areas of Uganda. Open journal of preventive medicine. 2015;5(3):132.

43. Ali SA, Dero AA, Ali SA. Factors affecting the utilization of antenatal care among pregnant women: a literature review. J Preg Neonatal Med 2018; 2 (2).

44. Fagbamigbe AF, Akanbiemu FA, Adebowale AS, Manuwa-Olumide A, Korter G. Practice, knowledge and perceptions of antenatal care services among pregnant women and nursing mothers in Southwest Nigeria. International Journal of Maternal and Child Health. 2013;1(1):7-16.

45. Gong E, Dula J, Alberto C, de Albuquerque A, Steenland M, Fernandes Q, Cuco RM, Sequeira S, Chicumbe S, Gudo ES, McConnell M. Client experiences with antenatal care waiting times in southern Mozambique. BMC health services research. 2019;19(1):538.

46. Gupta RK, Shora TN, Verma AK, Jan R. Knowledge regarding antenatal care services, its utilization, and delivery practices in mothers (aged 15-49 years) in a rural area of North India. Tropical Journal of Medical Research. 2015;18(2):89.

Data set

Click here to access/download
Supporting Information
Data set.xlsx

Title page

Women's knowledge and its associated factors regarding optimum utilisation of antenatal care in rural Ghana: a cross-sectional study

[1]Agani Afaya (MSc) [2]Thomas Bavo Azongo (PhD), [3]Veronica Millicent Dzomeku (PhD), [4]Hyeonkyeong Lee (PhD), [3]Richard Adongo Afaya (MPhil), [1]Solomon Mohammed Salia (MSc), [1]Peter Adatara (PhD), [5]Robert Kaba Alhassan (PhD), [6]Martin Amogre Ayanore (PhD), [7]Confidence Alorse Atakro (MN), [1]David Adadem (BN),[1]Emmanuel Opoku Asiedu (BN), [6]Paul Amuna (MB ChB, PhD)

[1]Department of Nursing, School of Nursing and Midwifery, University of Health and Allied Sciences, Ho, Ghana
[2]Department of Public Health, School of Allied Health Sciences, University for Development Studies, Tamale, Ghana
[3]Department of Nursing, Kwame Nkrumah University of Science and Technology, Kumasi, Ghana
[4]College of Nursing, Yonsei University, Seoul, South Korea
[5]Centre for Health Policy and Implementation Research. Institute of Health Research University of Health and Allied Sciences, Ho, Ghana
[6] Department of Health Policy Planning and Management, School of Public Health, University of Health and Allied Sciences, Ho, Ghana
[7]School of Nursing and Midwifery, Christian Service University College, Kumasi, Ghana

Correspondence

Agani Afaya (aagani@uhas.edu.gh)
Department of Nursing, School of Nursing and Midwifery, University of Health and Allied Sciences, Ho, Ghana

Abstract

Introduction: Improving maternal health is a global public health challenge especially in sub-Saharan Africa. The optimum utilisation of antenatal care by pregnant women is known to improve maternal health outcomes. Maternal morbidity and mortality rates in Ghana remain unacceptably high, particularly in rural settings where skilled delivery care often times is disproportionally low. This study assessed factors associated with optimum utilisation of antenatal care in rural Ghana.

Methods: A cross-sectional design was applied to collect data among eligible participants between October 2018 and January 2019. A total of 322 women who gave birth and attended the postnatal clinic were recruited for the study. Consecutive sampling was employed in recruiting participants. The associations between the dependent variables (ANC service utilization and knowledge of ANC) and independent variables (socio-demographic characteristics) were examined using ordinary least squares logistic regression at 95% confidence interval in STATA version 14.0.

Results: Of the 322 participants, 69.0% reported utilising at least four or more times ANC services. Determinants of women attending ANC for four or more times was significantly associated with age [OR=4.36 (95%CI: 2.16-8.80), p<0.001], educational level [OR=10.18 (95%CI: 3.86-26.87), p<0.001], and insured with National Health Insurance Scheme [OR=3.42 (95%CI: 1.72-6.82), p<0.001]. Not married [OR=0.65 (0.39 - 1.09), p=0.011] or divorced [OR=0.33 (95%CI: 0.13 – 0.83), p=0.019] was negatively associated with utilisation of four or more ANC services. The majority (79.0%) of the participants had a good level of knowledge regarding antenatal care.

Conclusion: Although the majority of women in this study had good knowledge of ANC services, a significant number of them did not complete the recommended number of ANC visits for at least four times during a normal pregnancy. Awareness and further education to

reproductive-age women on the significant role adequate ANC attendance plays in advancing health and well-being require further investments, particularly among rural women in Ghana.

Keywords Antenatal care utilisation, women's knowledge, factors, rural setting, Ghana

Introduction

Globally, about 303, 000 women and adolescent girls die as a result of pregnancy and childbirth-related complications [1] of which an alarming 99% of these maternal deaths occur in low-resource settings. Though globally, the number of women and girls dying due to pregnancy-related complications and childbirth decreased by nearly half (50%) from 1990 to 2013, the number of deaths within the West African region remains unacceptably high with Maternal Mortality Ratio (MMR) of 679 deaths per 100,000 live births in 2015 [2-4].

According to the 2017 Ghana Maternal Health Survey (GMHS), the MMR in Ghana is 310 per 100,000 live births, which remains unacceptably high. Data from the 2017 GMHS shows that nearly half of the women (50%) in rural settings of the Northern and Volta regions of Ghana deliver at home compared with the national average of 21% [5]. Delivering at home without the presence of a skilled birth attendant predisposes pregnant women to some life-threatening conditions. Facility-based delivery allows pregnant women to receive emergency care by skilled health professionals when complications arise which can help save the lives of both baby and mother [5]. Optimum antenatal care utilisation can pre-empt women the need for hospital delivery, thereby reducing possible pregnancy-related morbidities and mortalities.

Antenatal care (ANC) is the health care and education provided to pregnant women and adolescent girls by skilled health care professionals to ensure the best health conditions for the mother and the baby during pregnancy [1, 5]. Antenatal care utilisation is an important constituent of maternal health care, which reduces maternal and perinatal morbidity and

mortality both directly through identification and management of pregnancy-related complications, and indirectly through the identification of pregnant women and girls most likely to develop complications during labour and delivery, thus ensuring early referral to an appropriate health facility for further care [1]. Globally, while most women now attend at least one ANC visit (86%), only 62% attend four, with lower rates reported in sub-Saharan Africa and South Asia [6]. An analytical review of the recent WHO Global Health Observatory data repository shows that ANC coverage, between 2000 and 2017, was indirectly correlated with MMR worldwide [7, 8]. The evidence indicates that countries with poor ANC coverage are more likely to have high MMR [9-11]. For example, ANC utilisation in Australia is 94% with MMR of 6 per 100, 000, Finland has 99% ANC utilisation with MMR 3 per 100, 000 and France has 99% ANC utilisation with MMR 8 per 100000 live births. In comparison with sub-Saharan Africa, Nigeria has 49.1% ANC coverage with MMR of 917 per 100000, Cote d'Ivoire has 51.3% ANC coverage with MMR of 617 per 100,000, and Ghana has 89.3% ANC coverage with MMR 310 deaths per 100, 000 live births [7,8].

While ANC coverage remains high in Ghana, the coverage of at least four ANC visits remains lower at approximately 76% [12]. During pregnancy, the WHO recommends at least 4 ANC visits for antenatal care by a skilled health care professional for advice and monitoring of the health and well-being of both the mother and the developing foetus. A minimum of 4 visits constitutes receiving optimum ANC care. In this study, optimum ANC refers to a woman who made at least 4 visits for ANC during pregnancy. Geographically, despite the national rate of 76% for ANC care, disparities exist particularly among rural and poor socio-economic groups in Ghana on health care utilisation. To advance maternal and reproductive health outcomes for women throughout their life cycle and contribute to reducing current MMR, there is a need for more context evidence across Ghana in relation to an important outcome measure such as pregnant women's knowledge and optimum ANC during pregnancy. This study, therefore,

sought to assess women's knowledge and its associated factors that influence optimum utilisation of ANC services (4+ visits) in a context that presents opportunity for health systems learning and policy recommendations as Ghana steps up efforts to ensure universal access to reproductive health care and for the attainment of the Sustainable Development Goals (SDGs) targets on maternal and reproductive health.

Conceptual framework

Deciding to utilise ANC services does not only depend on the individual's preference but also the factors that facilitate the accessibility of the health facility (services) or the factors that impede the utilisation of ANC services [13]. WHO outlined that a woman's ability to access antenatal care services is influenced by several factors that include distance or time taken to travel to a health facility, availability of ANC services, social and cultural factors that can serve as barriers to access, economic status, costs related with utilisation of services, and the ANC quality of services provided [14].

Several models have been developed over the last few decades to identify factors influencing healthcare service utilisation, and the most widely used is the Anderson Behavioral Health care model known as Behavioral Model of Health Services. This study was grounded based on the Andersons Behavioral Model and sought to identify factors that facilitate or impede the use of healthcare and to understand discrepancies in the utilisation of care [15]. This model was first developed to determine why families utilise health care, to identify equitable access to health services, and to support in formulating policies that will promote equitable access [16, 17]. According to the model, utilisation of health services is determined by three dynamics: (1) the predisposing factors, (2) enabling factors, and (3) need factor. Predisposing factors include individual characteristics and health beliefs; enabling factors are the logistic aspect of obtaining

care and need factors are the most immediate cause of healthcare utilisation such as the presence of disease condition and self-perceived functional or health status [16].

The current study sought to assess factors that influence (facilitate or impede) women's use of ANC services; community-based factors that impede or motivate the use of ANC, facility-based factors, and adequacy of ANC visits. Despite the importance of these individual determinants affecting ANC utilisation among women, the current study also sought to assess women's knowledge of ANC services and how that influence ANC use as evident in literature [18] recommended by WHO for pregnant women. The study identified the following (as shown in Figure 1) factors that influence ANC utilisation (either positive or negative) in the study setting among women.

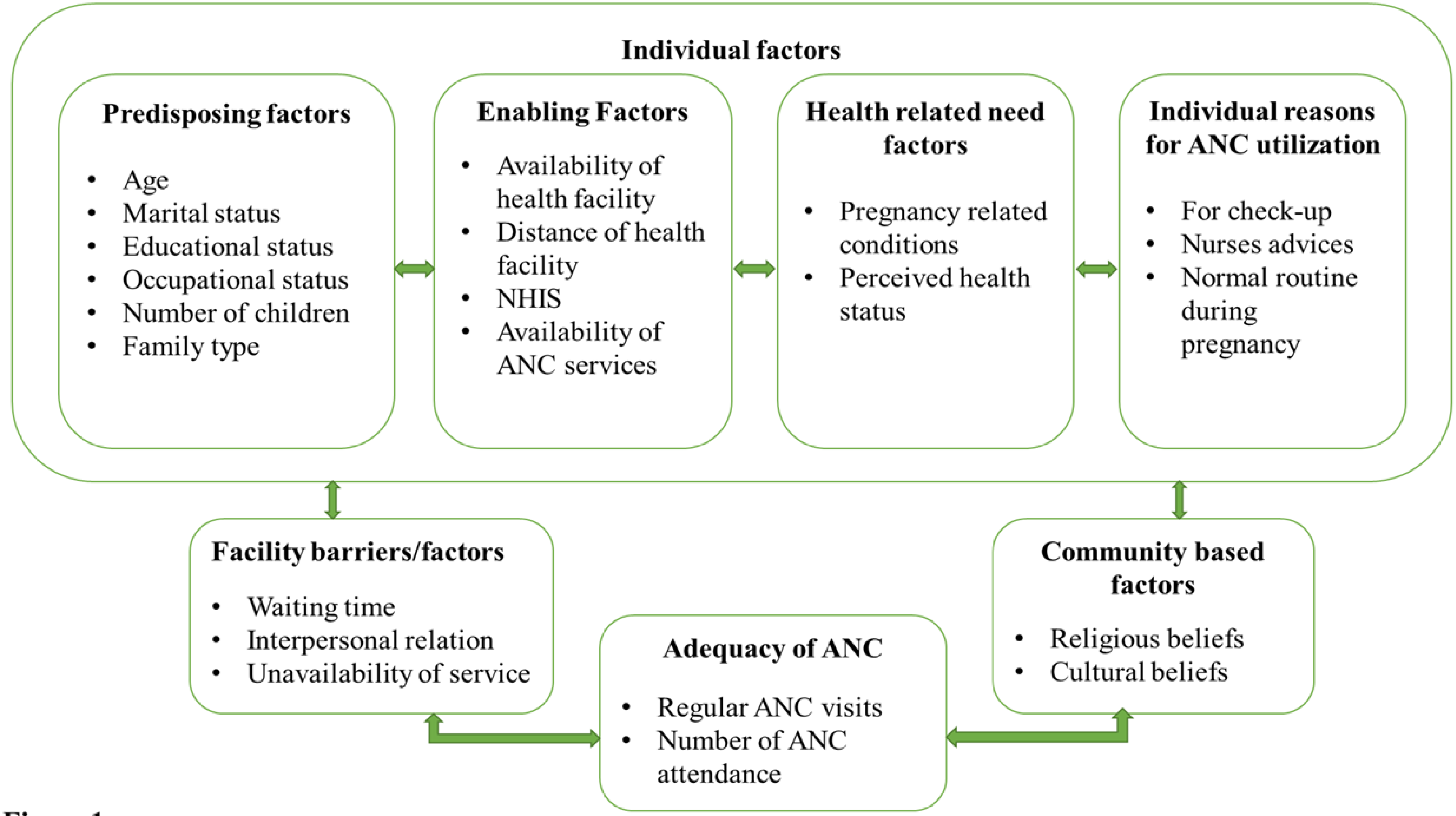

Figure 1:

- Conceptual framework for analyzing factors associated with ANC utilization among women in Northern Ghana
- Adapted from Andersen's behavioral model for health service use (Andersen, 1995)

134

Methods

Study Design

The study applied a cross-sectional analytical facility-based study design to recruit the study participants. Given the design type, quantitative data was collected among eligible study participants after applying the appropriate sampling methodology. This design was appropriate to enable the study team to elicit answers to key questions that underpin the study.

Study setting and population

The study was conducted at four health facilities in Bamboi, a sub-district jurisdiction under the Bole district in the Savanah region of Ghana. The health facilities were categorised as (A, B, C & D). Facility A had two midwives, four Enrolled Nurses (EN) and a community health nurse (CHN), facility B had two CHNs, facility C had a midwife and one CHN and facility D had male practicing midwifery. These health professionals provide maternal and child health services for mothers attending any of these facilities. The Bole District is one of the seven districts located in the newly created Savanah region of Ghana. The District is located at the extreme western part of the northern region of Ghana. The District is bordered to the North by Sawla / Tuna Kalba District, to the West by the Republic of Ivory Coast, to the East by West Gonja District and to the South by Wenchi and Kintampo Districts of Brong-Ahafo region. The District health services are divided into four sub-districts namely Bole, Tinga, Jama, and Bamboi. Each sub-district has an operational area served by a health facility. Bamboi is one of the sub-districts which is to the South of the Bole district. The sub-district, Bamboi where this study was conducted has a relatively small semi-urban area while a large proportion is rural. The Bamboi sub-district has one health center and three Community-Based Health Planning and Services (CHPS) zones serving over 26 communities within the sub-district. In Ghana, CHPS is the lowest level under the health level structures where health care is provided at the community level, closer to the people. The eligible study population were all reproductive-

aged women in the Bambi sub-district who attended the study health facilities in Bamboi sub-district for postnatal care during the time of the study.

Sampling Method and Sample Size Determination

Consecutive sampling was employed in recruiting participants. The average monthly attendance for the four facilities providing postnatal care services was estimated to be about 450. The total population for the four facilities for four months was 1800. The sample size for the study was determined by using the sample size calculation by Yamane [19]. The minimum required sample size was 310 and a 5% non-response rate was added to get a sample size of 340. Finally, 322 questionnaires were considered valid to be included for analysis.

Outcome variables

During data analysis, the outcome variables of interest were knowledge and utilisation of ANC services. The utilisation of ANC services was classified as adequate versus inadequate. The study assessed the overall adequacy of ANC visits by reviewing the mothers' ANC cards to determine the frequency of visits during the immediate past pregnancy. Antenatal care was classified as adequate if the woman had at least four or more antenatal visits during normal pregnancy and less than four visits were defined as inadequate. The knowledge of mothers on ANC services was classified as good knowledge and bad knowledge.

Explanatory variables

The explanatory variables were selected based on review of related literature and the modified version of the Anderson Healthcare Utilization model. Socio-demographic characteristics; Participants' age was categorised into <20yrs, 21-30yrs and 31-46 years. Marital status was categirised into not married, and married. Educational level of participants

was categorised as none, basic, secondary and tertiary. Occupation of participants was classified into farmer, trader, civil servant, and others. The religious status was categorised under Christianity and Islam. Mo, Gonja, Ewe, Akan, and Dagaaba were the ethnic groups of participants. The family type was the nuclear and extended family. The number of children was categorised into 1-2, 3-4 and 5+ children. Participants were either insured or not insured with the National Health Insurance Scheme (NHIS). Other variables of interest were distance to health facility (For CHPS, the distance was between a radius of 5 km (and for Health Centres between 8 and 16 km radius [20-22]. Women who trekked more than 5km and also 16km where considered far and those within the recommended limit were considered short), person who provides healthcare, person who makes healthcare decisions and means of transportation to the facility.

Data collection procedure

Data collection lasted for four months from October 2018 to January 2019. A self-administered semi-structured and pretested questionnaire was used to collect data from eligible participants. Four trained research assistants (nurses) were assisted to collect data in the four study health facilities. The research assistants were trained for 2 days and were supervised for a week by the principal investigator during data collection. The four facilities were visited every week during the postnatal days for questionnaire administration. Eligible participants were approached after receiving postnatal care and provided with further education on the study's aim and objectives. Eligible participants who were willing to participate were offered consent forms to endorse. Questionnaires were interviewer-administered by the study team. Completeness of questionnaires was checked daily after completing each questionnaire before participants were asked to leave. Where minor omissions were detected, immediate corrective measures were taken with participants to ensure validity and to prevent missing information.

Validity and reliability of the study instrument

The study instrument was developed after reviewing relevant related literature and also adopting Anderson health care behavioral model. Preceding data collection, the validity and reliability of the study instrument were assessed through various stages. To assess the content validity, a group of researchers and clinicians were made to review and refine the items in the questionnaire. This group comprised of two senior community midwives and two researchers in the field of maternal and child health. Further, the Pearson correlation coefficient was used to determine the test-retest reliability of the study with a sample of 20 participants. The instrument was administered to the same participants' after one week. The test-retest correlation coefficient for the instrument was 0.73 ($p < 0.001$), which is indicative of acceptable stability over time.

Questions asked on ANC knowledge

1. ANC can prevent complications in pregnancy?
2. Pregnant women may have problems without ANC?
3. Regular ANC medications can promote optimal growth of unborn child?
4. Health facility delivery is safer and better than home delivery?
5. Do you know the recommended place for delivery?
6. Do you know the recommended frequency and timing of ANC visits?
7. ANC is recommended regardless of complications?
8. Do you know items to prepare for before delivery?
9. Do have knowledge of family planning?
10. Do have knowledge of malaria prevention?

Figure 2: Key items used to assess women's knowledge of ANC services

Ethical consideration

Ethics approval for the study was obtained from the University of Health and Allied Sciences Research Ethical Committee (UHAS-REC A. 10[27] 17-18). Permission to conduct the study was sought from the various facilities before the commencement of data collection. Both verbal and written consent was obtained from participants after agreeing to participate in the study. Participants were assured of anonymity, confidentially and privacy. Participants were made aware of the right to withdraw from the study at any particular point in time if they wished to do so.

Data Analysis

Data were entered into SPSS software, cleaned and validated to ensure completeness and quality before analysis. The cleaned data were exported to STATA version 14.0 for analysis. Descriptive statistics were applied to represent frequencies and percentages. The overall level of knowledge on antenatal care was measured by scoring responses that measured participants' knowledge (figure 2) using the following descriptions; a score of 1 was assigned if the participant had knowledge and 0 if they had no knowledge. Total score and mean scores were computed and a score below the mean was considered poor knowledge whiles a mean score or above was considered to be a good knowledge. Ordinary least square logistic regression analysis, computing odds ratio, was used to determine the association between dependent variables (ANC service utilisation and knowledge of ANC) and socio-demographic characteristics. Statistical significance was determined at 95% confidence level.

Results

Participants' socio-demographic characteristics

Slightly more than half, 177 (55.0%) of participants were aged 21-30 years while about half, 167 (51.9%) were not married. A quarter of the women, 111 (34.5%) had basic and secondary education, and more than half, 188 (58.4%) engaged in trading. About 144 (44.7%) belonged to the Dagaaba ethnic group. The majority, 288 (89.4%) had the nuclear family type and over two-thirds had 1-2 children. The majority, 277 (86.1%) had to walk to access healthcare. Most, 210 (65.2%) made their own decision regarding their healthcare needs (Table 1).

Table 1: Participants' socio-demographic characteristics (N=322)

Variable	Frequency (f)	Percentage (%)
**Age**		
≤ 20	44	13.6
21-30	177	55.0
31-40	101	31.4
**Marital Status**		
Married	133	41.3
Not married	189	58.7
**Educational status**		
None	33	10.2
Basic	111	34.5
Secondary	111	34.5
Tertiary	67	6.8
**Employment**		
Employed	122	37.9
Unemployed	189	58.7
Student	11	3.4
**Occupation**		
Farmer	56	17.4
Trader	188	58.4
Civil servant	56	17.4
Others	22	6.8
**Religion**		
Christianity	255	79.2
Islam	67	20.8
**Ethnicity**		
Mo	100	31.1
Gonja	33	10.3
Ewe	11	3.4
Akan	34	10.5
Dagaaba	144	44.7
**Family type**		
Nuclear	288	89.4
Extended	34	10.6
**Number of children**		

1-2 children	245	76.1
3-4 children	55	17.1
5+ children	22	6.8
**Number of ANC visits**		
<4	100	31.0
4+	222	69.0
**Health insurance**		
Insured	245	76.1
Not insured	77	23.9
**Distance to health facility**		
Short	220	68.3
Far	102	31.7
**Transportation to ANC**		
Motorcycle	23	7.1
Foot	277	86.1
Others	22	6.8
**Person who provides for healthcare**		
Self	90	27.9
Partner	166	51.6
Parents	66	20.5
**Person who makes healthcare decision**		
Self	210	65.2
Partner	101	31.4
Parents	11	3.4

Factors associated with the utilization of antenatal care services

Logistic regression analysis showed that women who were aged 21-30 years were 4 times more likely to utilise ANC services four or more times compared to those aged 20 years and below [OR=4.36 (95%CI: 2.16-8.80), p<0.001] and those who were not married were 70% less likely to utilise ANC services at least four times compared to those who were married [OR=0.30 (95%CI: 0.18 – 0.50), p<0.001]. Women who had tertiary education were 10 times more likely to utilise ANC services four or more times than those without education [OR=10.18 (95%CI: 3.86-26.87) p<0.001]. Participants who were insured with the NHIS were 3 times more likely to utilise the ANC compared to those who were not-insured [OR=3.42 (95%CI: 1.72-6.82), p<0.001]. Regarding distance to health facilities, women who reported they stayed far from

health facilities were 80% less likely to utilise ANC compared to women who stayed closer to health facilities [OR=0.20 (95%CI: 0.12-0.34), p<0.001].

After adjusting for other explanatory variables women who were not married were 84% less likely to utilise ANC services four or more times [AOR=0.16 (95%CI: 0.01 – 0.75), p=0.03]. Women who had tertiary education were 10 times more likely to utilise ANC services four or more times than those without education [AOR=5.26 (95%CI: 2.15-17.28) p<0.001]. Women who stayed far from the health facilities were 65% less likely to attend ANC for at least four times [AOR=0.35 (95%CI: 0.02-0.13), P=0.001]

Table 2: Logistic regression results showing optimum utilization of ANC services

Variable	Usage of ANC Services		COR [95% CI]	p-value	AOR [95% CI]	p-value
	<4=**100 n(%)**	**4+=222 n(%)**				
**Age**						
≤20	22 (22.0)	22 (9.9)	**Ref**		**Ref**	
21-30	33 (33.0)	144 (64.9)	4.36[2.16-8.80]	<0.001	2.01[0.32-5.70]	0.043
31-46	45 (45.0)	56 (25.2)	1.24[0.61-2.53]	0.546	0.24[2.16-8.80]	0.067
**Marital Status**						
Married	33 (33.0)	100 (45.0)	**Ref**			
Not married	67 (67.0)	122 (55.0)	0.30 [0.18 – 0.50]	<0.001	0.16 [0.01 – 0.75]	0.030
**Educational status**						
None	22 (22.0)	11 (5.0)	**Ref**		**Ref**	
Basic	22 (22.0)	89 (40.1)	8.09 [3.42 -19.14]	<0.001	3.19 [2.35-16.75]	0.056
Secondary	45 (45.0)	66 (29.7)	2.93 [1.29- 6.64]	0.010	1.29 [1.29- 6.64]	0.045
Tertiary	11 (11.0)	56 (25.2)	10.18[3.86-26.87]	<0.001	5.26[2.15-17.28]	<0.001
**Occupation**						
Farmer	33 (33.0)	23 (10.4)	**Ref**			
Trader	45 (45.0)	143 (64.4)	4.55[2.40- 8.55]	<0.001		
Civil servant	11 (11.0)	45 (20.3)	5.87[2.51-13.69]	<0.001		
Others	11 (11.0)	11 (4.9)	1.43[0.53-3.86]	0.475		
**Religion**						
Christianity	44 (44.0)	211 (95.1)	**Ref**		**Ref**	
Islam	56 (56.0)	11 (4.9)	0.04[0.02-0.08]	<0.001	0.017[0.01-0.29]	0.088
**Family type**						
Nuclear	88 (88.0)	200 (90.1)	**Ref**			
Extended	12 (12.0)	22 (9.9)	0.81[0.38-1.70]	0.573		
**Health insurance**						
Not Insured	11 (11.0)	66 (29.7)	**Ref**			

Insured	89 (89.0)	156 (70.3)	3.42[1.72-6.82]	<0.001		
**Distance to health facility**						
Short	44 (44.0)	176 (79.3)	**Ref**		**Ref**	
Far	56 (56.0)	46 (20.7)	0.20[0.12-0.34]	<0.001	0.35[0.02-0.13]	0.001
**Provider for healthcare**						
Self	34 (34.0)	56 (25.2)	**Ref**			
Partner	33 (33.0)	133 (59.9)	2.44 [1.38-4.33]	0.002		
Parents	33 (33.0)	33 (14.9)	0.60 [0.32-1.16]	0.129		

COR=Crude Odds ratio, **AOR**=Adjusted Odds ratio, adjusted for age, marital status, educational level, religion, and distance to health facility.

Participants Knowledge of Antenatal Care Services

Figure 3 shows that 79.2% of the participants had good level of knowledge regarding of antenatal care while 20.8% had poor knowledge regarding antenatal care.

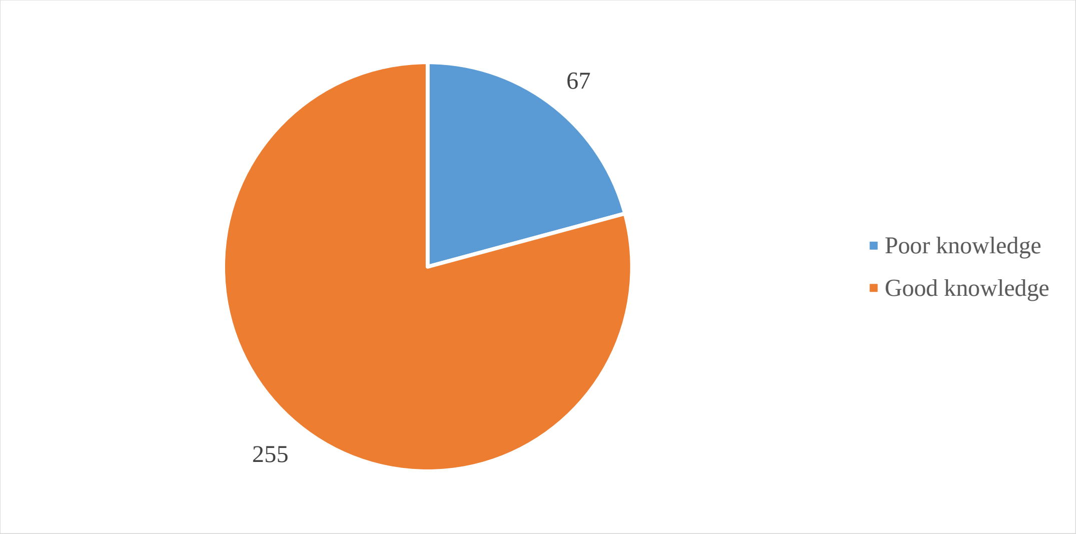

Figure 3: Overall level of knowledge regarding ANC

Association between Socio-demographics characteristics of participants and knowledge of antenatal care

Not being married and divorced women were 75% and 91% respectively less likely to have good knowledge regarding ANC [OR=0.25, (95%CI: 0.13 – 0.51), p<0.001; OR=0.09, (95%CI: 0.03 – 0.26), p<0.001]. Women who attained basic school education were 4.47 times more likely to have good knowledge of ANC compared to those with no education [OR=4.47, (95%CI: 1.75 – 11.39), p=0.002]. Respondents who belonged to the Islamic religion were 85% less likely to have good knowledge on ANC compared to respondents who were Christians [OR=0.15, (95%CI: 0.08 – 0.27), p<0.001]. Regarding family status and its association with women use of ANC services, women who lived in an extended family system were 99% less likely to have good knowledge on ANC compared to those who lived in a nuclear family environment [OR=0.01, (95%CI: 0.00 – 0.03), p< 0.001] (see table 3).

Multi-variable logistic regression analysis revealed that respondents aged >30 years were 98% less likely to have adequate knowledge on antenatal care compared to those aged 20 years and below [AOR=0.02, (95%CI: 0.00 – 0.45), p=0.013]. Not married women were 99% less likely to have adequate knowledge on antenatal care compared to married women [AOR=0.01, (95%CI: 0.00 – 0.28), p<0.001]. Women who had 5+ children were 98% less likely to have adequate knowledge on antenatal care compared to those with 2 children or less [AOR=0.02, (95%CI: 0.00 – 0.43), p=0.012].

Table 3; Logistic regression results of factors associated with women's knowledge of ANC services

Variable	Knowledge of ANC Services		COR [95% CI]	p-value	AOR[95% CI]	p-value
	**Poor n=67 (%)**	**Good n=255 (%)**				
**Age (years)**						
≤ 20	0 (0.00)	44 (17.2)	**Ref**		**Ref**	
21-30	33 (49.3)	144 (56.5)	0.05 [0.00 – 0.81]	0.035	0.10 [0.01 – 1.76]	0.115
>30	34 (50.7)	67 (26.3)	0.02 [0.00 – 0.36]	0.008	0.02 [0.00 – 0.45]	0.013
**Marital Status**						
Married	11 (16.4)	122 (47.8)	**Ref**		**Ref**	
Not married	56 (83.6)	1323(52.2)	0.25[0.13 – 0.51]	<0.001	0.01 [0.00 – 0.28]	<0.001
**Educational status**						
None	11 (16.4)	22 (8.6)	**Ref**			
Basic	11 (16.4)	100 (39.2)	4.47[1.75 –11.39]	0.002		
Secondary	34 (50.8)	77 (30.2)	1.15[0.51 – 2.60]	0.740		
Tertiary	11 (16.4)	56 (22.0)	2.51[0.96 – 6.50]	0.058		
**Occupation**						
Farmer	11 (16.4)	45 (17.6)	**Ref**			
Trader	45 (67.2)	43 (56.1)	0.80[0.38 - 1.65]	0.541		
Civil servant	11 (16.4)	45 (17.7)	0.10 [0.40 – 2.50]	0.001		
Others	0 (0.00)	22 (8.6)	11.37[0.64–21.83]	0.098		
**Religion**						
Christianity	33 (49.3)	222 (87.1)	**Ref**		**Ref**	
Islam	34 (50.7)	33 (12.9)	0.15[0.08 – 0.27]	<0.001	0.23 [0.08 – 0.71]	0.010
**Ethnicity**						
Mo	0 (0.00)	100 (39.2)	**Ref**			
Gonja	22 (32.8)	11 (4.3)	0.01[0.00 – 0.04]	<0.001		
Ewe	0 (0.00)	11 (4.3)	0.11[0.00 – 6.04]	0.280		
Akan	12 (17.9)	22 (8.7)	0.01[0.00 – 0.16]	0.001		
Dagaaba	33 (49.3)	111 (43.5)	0.02[0.00 – 0.27]	0.004		

**Family type**						
Nuclear	33 (49.2)	255 (100)	**Ref**		**Ref**	
Extended	34 (50.7)	0 (0.00)	0.01 [0.00 – 0.03]	<0.001	0.01 [0.00 – 0.02]	<0.001
**Number of children**						
≤ 2 children	45 (67.1)	200 (78.4)	**Ref**		**Ref**	
3-4 children	11 (16.4)	44 (17.3)	0.89 [0.42 – 1.81]	0.725	0.02 [0.00 – 2.82]	0.116
5+ children	11 (16.4)	11 (4.3)	0.23 [0.09 – 0.55]	0.001	0.02 [0.00 – 0.43]	0.012

COR=Crude Odds ratio, **AOR**=Adjusted Odds ratio, Adjusted for age, marital status, religion, family type, number of children.

Community and individual challenges affecting antenatal utilization

Long-distance to the health facility (31.1%) and partner's perception of the importance of ANC were major barriers to the effective utilisation of ANC services by the women. Also, about 27.6% indicated not having enough money to attend antenatal clinic and cultural beliefs (7.9%) as some of the challenges. This is shown in (Figure 4).

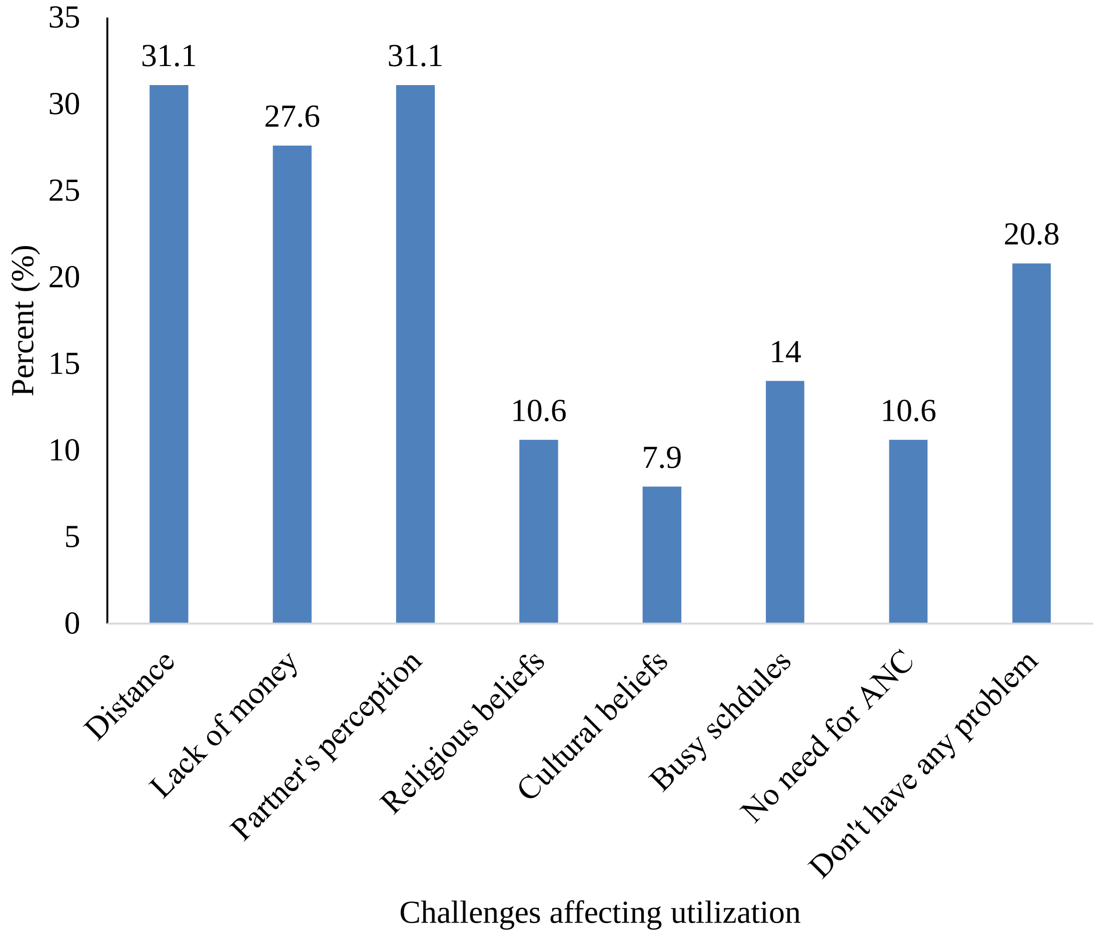

Figure 4: Challenges affecting utilization of ANC

Facility related barriers to ANC attendance

Figure 5 showed that most, 38.2% of the participants were faced with long waiting times in health facilities where they assessed care, followed by 28% who were scolded by staff and 24.5% who had inconvenient service hours.

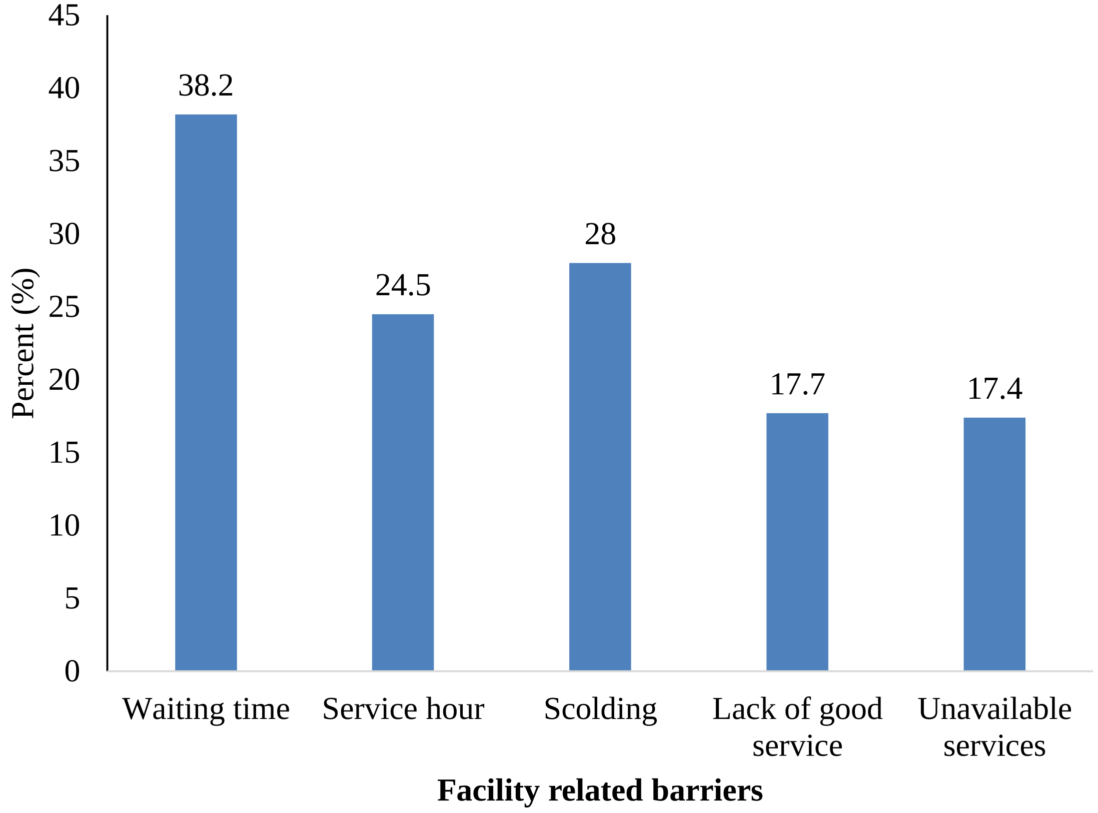

Figure 5: Facility related barriers to ANC utilization

Reasons for ANC Attendance

Figure 6 also showed that majority, 76.1% of the respondents went for antennal services for check-ups, while in 24.2% of them, it was because of normal routine check-ups during pregnancy. Also, about one-tenth, 10.6% went for antenatal care services as a result of health workers' advice.

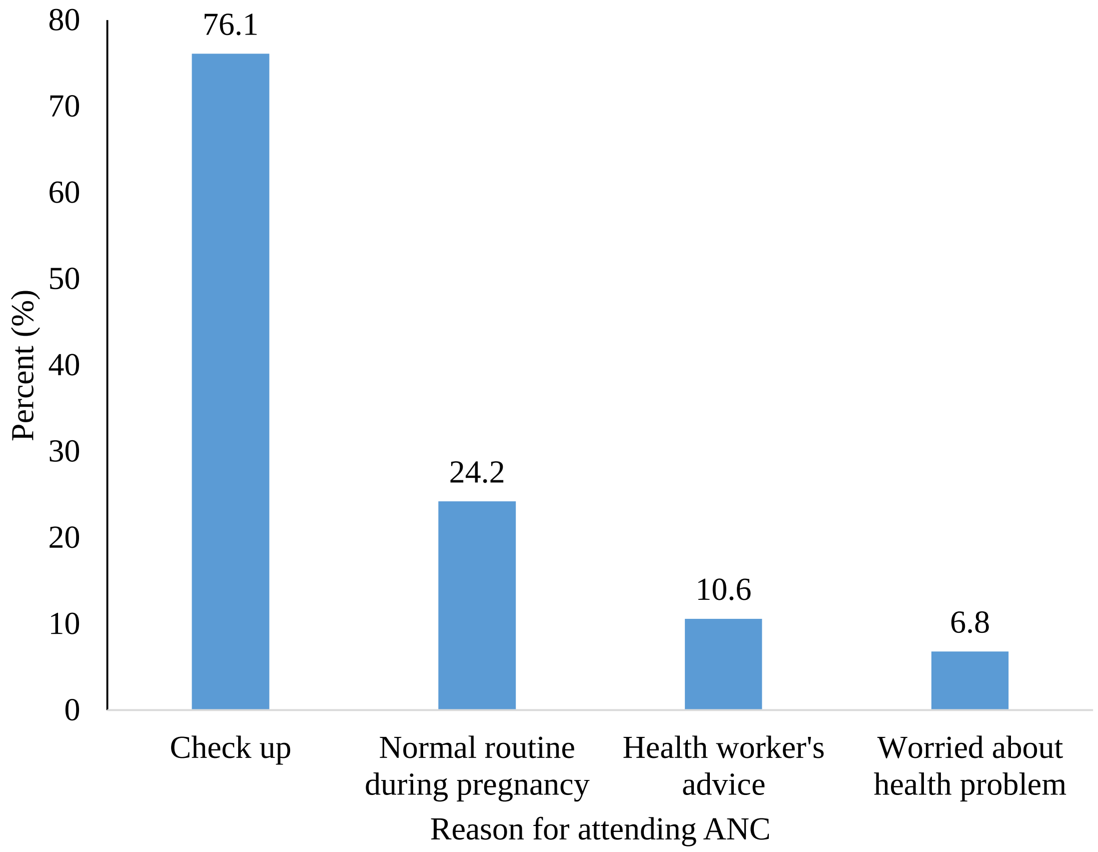

Figure 6: Reason for attending ANC

Discussion

This study applied an integrated conceptual framework to identified individual and facility based factors that influence optimum utilisation of ANC services among mothers in rural areas. Adequacy of ANC services was also assessed. And for a pregnant woman to attain optimum

utilisation or adequacy of ANC services, the World Health Organization recommends four or more ANC visits for women who do not experience any complications throughout their pregnancies [23]. The current study finding revealed that 69.0% of the women attended ANC at least four or more times. This study corroborates results in Ethiopia [24] but higher than some earlier studies in Rwanda [3], Ethiopia [25], and Kenya [26]. Though this study result is high as compared to other countries, it is low when compared to findings in previous studies in Ghana [27] Nepal [28] and Nigeria [9]. The divergence could be due to socio-cultural, economic, and awareness differences among the study populations as purported by other studies [25, 29]. The present finding of 69% is below the national coverage of at least four ANC visits of 75.9% [12]. This difference may be due to the lack of awareness of optimal ANC visits. Also, the differences could have been caused by other potential inhibiting factors such as distance to health facilities and socio-economic factors. Therefore, there is the need to intensify interventions implemented by the Ghana Health Service over the years such as Social and Behaviour Change Communication (SBCC) targeting reproductive-age women on optimum ANC utilisation and the importance of early initiation and regular ANC visits during pregnancy. In addition, current national policy initiatives to extend the Community-based Health Planning Services (CHPS) concept to cover the entire country in order to meet the reproductive health needs of many women, particularly in rural areas of Ghana, remain a laudable intervention in Ghana [12]. CHPS offer opportunity to bridge distance barriers and provide reproductive-age women better chances of obtaining relevant and useful information on ANC and its importance throughout pregnancy as well as serve as an effective Primary Health Care (PHC) strategy to improve access to maternal health and capacity building for improved client-service provider relationship.

Participants' educational level was found to be a strong predictor of optimum utilisation ANC services for four or more times. Participants who had basic, secondary, and tertiary education were more likely to use ANC services compared to those who had no education. This finding is not counter-intuitive because previous studies in developing countries have reported that the educational level of women increases the odds of utilising ANC services for four or more times [9, 23, 30-33]. Probably the educated women are more inclined to issues of their health (identifying danger signs) thereby placing more priority on their health and that of the unborn child. The major cause of health inequality in ANC coverage has been strongly associated with women's poor/lack of education [34]. This highlights the need for a multisectoral approach, with the health sector working jointly with other sectors such as the educational sector to promote female education to the highest level.

Participants who were not married or divorced were less likely to attend ANC for at least four or more times relative to their counterparts who were married. In a Ghanaian community, women who are not married are expected to remain virtuous until marriage [35] in order to prevent public ridicule, so women who get pregnant without a husband could avoid ANC visits [27] to prevent public ridicule. On the other hand, women who are not married and those with low socio-economic status are likely to lack the financial means (for travel cost and medicines) and may be worse affected when it comes to meeting the number of recommended ANC care services during pregnancy. The free fee delivery care policy introduced by the Government of Ghana through the NHIS continues to play a significant role in enhancing lower socio-economic groups' access and use of maternity care services, including ANC care. However, bottlenecks such as poor re-imbursements issues under the NHIS impact negatively on pro-poor women's access to ANC care and other general health care needs. Health system improvements that enhance the efficiency of

the NHIS and broaden the benefits package for women reproductive health care services can go a long way to improve long term health gains for maternal health outcomes in Ghana.

Women who were insured with the NHIS were three times more likely to utilise ANC services for four or more times compared to those who were not insured. This finding is consistent with other studies in Ghana [27, 36-38]. While the number of visits a pregnant woman makes for ANC is key to attaining optimum ANC, the quality of ANC services is also relevant. Ayanore and colleagues in a study on focused ANC among women found that ANC quality predict the adequacy of ANC services relative to the frequency of visits made by a woman [39]. Ghana's implementation of a free delivery policy in July 2008 for pregnant women has played a major role in improving maternal and child health outcomes in Ghana. The NHIS which allows pregnant women access to benefit from this free fee policy has also assisted to improve health care access and utilisation for all age groups. This study findings show that being insured under the NHIS can enhance women's attainment of optimum ANC. Thus, the implementation of the free maternal health care policy under the NHIS instituted by the government of Ghana is key to achieving increased utilisation of ANC services for at least four times during every normal pregnancy [40].

Despite the potential benefit of the NHIS, registration challenges for enrolments often resulting in long queues making it difficult for pregnant women to register on time is documented [27, 41]. There is evidence in Ghana that demonstrates that poor NHIS client registration is mostly limited to the district capital because of the biometric system used; where people including pregnant women will have to trek long distances to register [27, 41]. The NHIS system has over the last few years introduced mobile phone technology to improve client registrations and address current challenges in enrolments under the NHIS. However, non-educated, pro-poor, and rural women are

likely not to benefit from high technological applications on NHIS registrations. NHIS reforms must prioritize how to improve client's engagements for the rural poor, and enhance vulnerable groups' chances to obtain NHIS. New policy reforms that will enhance access for NHIS enrolments in distant and rural areas are recommended in addition to new technological strategies being currently implemented by the NHIS

Women who trek far distances to the health facility for ANC services were 80% less likely to utilise ANC services for four or more times compared to those with trek shorter distances. The current study finding corroborates studies in Uganda and Pakistan where incomplete ANC attendance was attributed to long distance to the health facility [42 43]. Establishment of health facilities within catchment areas in rural communities, employing more qualified health personnel to deliver healthcare to the women in the communities, and access to good roads among others by the government of Ghana is significant in ensuring increased ANC utilisation.

Women's knowledge of ANC is crucial in the utilisation of ANC services during pregnancy. This study revealed that 79.2% of the women had good knowledge of ANC services. The determinants of women having knowledge of ANC services included; woman's age, woman's educational status, husband's educational status, religion, ethnicity, and the number of children. Our study finding is consistent with a study conducted in the southwest of Nigeria [44] but disagrees with a study conducted in Mozambique where the researchers found women's knowledge of ANC services not to have significant importance in their utilisation of ANC services [45]. It is established that women with good knowledge of ANC services have a better understanding and acceptance of the services provided during ANC [46]. Women's chances to have a good knowledge of ANC care services is thus a catalyst for improving ANC care-seeking. National-level policies that ensure focused ANC services, become an integral part of ANC and other maternal care

services are fundamental to supporting improve knowledge, address myths and remove other health care seeking challenges women encounter when using ANC care services.

On the aspect of facility-based factors that hinders ANC utilisation, the study found that waiting for a long time in the facilities to receive care, was the major implicating factor for facility-based barriers to ANC utilisation. This finding is congruent with a study conducted in Mozambique where the women identified long waiting times as a barrier to ANC utilisation [45]. The long waiting time identified by participants as a barrier to ANC service utilisation could be due to the shortage of qualified healthcare professionals needed to provide care to over 26 communities in the study setting. Pregnant women might find it so distressing waiting for long hours without receiving care and forgoing certain duties which might have financial implications on them and this may demotivate women for subsequent ANC service utilisation. Gong et al., [45] further support our assertion by illustrating that long waiting times have direct and indirect cost implications for clients seeking care and that attending to clients on schedule mitigates cost and also improves women accessing ANC services.

Limitations of the study

First, recall bias could have limited the validity of the data collected as some participants might have forgotten about past events involving ANC services. To minimize recall bias, the study assessed the immediate past ANC records and we crosschecked self-reported information against the ANC attendance book for the validity of the information. Secondly, the study design was cross-sectional, hence results should be interpreted with caution since this study did not provide an opportunity to assess cause-effect relationship. Also, several individuals, households, and health system factors influence optimum ANC services, which may have been missed and not included

in this study. This study was facility-based, hence the opportunity to miss out on eligible participants at the population level is acknowledged. Notwithstanding, this study provides useful information on women's knowledge and factors that influence optimum antenatal care utilisation in rural settings in Ghana.

Conclusion

Although the majority of the women had good knowledge of ANC services, a significant number did not complete the recommended number of ANC visits for at least four times during a normal pregnancy. The creation of awareness of adequate ANC attendance in rural settings should be emphasized by frontline health workers especially nurses and midwives working in these communities. Not insured with NHIS lowered the chances for women to have received optimum ANC services. Health system reforms that address NHIS access challenges are essential to support women attain optimum ANC and supportive focused ANC care in Ghana.

Further research is recommended to determine sociocultural, traditional, religious and other related practices at the community level on the utilisation of antenatal care services.

Abreviations

ANC: Antenatal care

CHPS: Community-based Health Planning Services

EN: Enrolled Nurses

GMHS: Ghana Maternal Health Survey

MMR: Maternal Mortality Ratio

NHIS: National Health Insurance scheme

SBCC: Social and Behaviour Change Communication

WHO: World Health Organisation

Conflict of interest

Authors declared no competing interest.

Data Availability Statement:

All relevant data are within the manuscript.

Funding

The study received no external funding

Acknowledgments

Our profound gratitude goes to the health facilities for their support and co-operation during data collection. We are also grateful to the women who took time off their busy schedules to participate in the study.

Author Contributions:

Conceptualization: Agani Afaya, Thomas B. Azongo, Veronica Millicent Dzomeku, Richard Adongo Afaya, Solomon Mohammed Salia, David Adadem, mmanuel Opoku Asiedu

Data curation: Agani Afaya, Richard Adongo Afaya, Solomon Mohammed Salia, David Adadem, Emmanuel Opoku Asiedu

Formal analysis: Agani Afaya, Peter Adatara, Robert Kaba, Martin Amogre Ayanore, Confidence Alorse Atakro

Funding acquisition: No funding

Investigation: Agani Afaya Thomas B. Azongo, Veronica Millicent Dzomeku, Hyeonkyeong Lee, Richard Adongo Afaya, [1]Solomon Mohammed Salia, Peter Adatara, Robert Kaba Alhassan, Martin Ayanoore, Confidence alorse Atakro, Paul Amuna

Methodology: Agani Afaya Thomas B. Azongo, Veronica Millicent Dzomeku, Hyeonkyeong Lee, Richard Adongo Afaya, Solomon Mohammed Salia, Peter Adatara, Robert Kaba Alhassan, Marin Amogre Ayanore, Confidence alorse Atakro, Paul Amuna

Project administration: Agani Afaya Thomas B. Azongo, Veronica Millicent Dzomeku, Hyeonkyeong Lee, Richard Adongo Afaya, [1]Solomon Mohammed Salia, Peter Adatara, Robert Kaba, Martin Amogre Ayanore, Confidence Alorse Atakro, Paul Amuna

Resources: Agani Afaya, Richard Adongo Afaya

Software: Agani Afaya

Supervision: Agani Afaya, Thomas B. Azongo, Veronica Millicent Dzomeku, Hyeonkyeong Lee, Peter Adatara, Robert Kaba, Martin Amogre Ayanore, Paul Amuna

Validation: Agani Afaya, Thomas B. Azongo, Veronica Millicent Dzomeku, Hyeonkyeong Lee, Peter Adatara, Robert Kaba, Martin Amogre Ayanore, Paul Amuna

Visualization: Agani Afaya, Thomas B. Azongo, Veronica Millicent Dzomeku, Hyeonkyeong Lee, Richard Adongo Afaya, [1]Solomon Mohammed Salia, Peter Adatara, Robert Kaba, Martin Amogre Ayanore, Confidence Alorse Atakro, Paul Amuna

Writing – original draft: Agani Afaya, Richard Adongo Afaya

Writing – review & editing: Agani Afaya, Thomas B. Azongo, Veronica Millicent Dzomeku, Hyeonkyeong Lee, Richard Adongo Afaya, Solomon Mohammed Salia, Peter Adatara, Robert Kaba, Martin Amogre Ayanore, Confidence Alorse Atakro, Paul Amuna

Reference

1. World Health Organization. WHO recommendations on antenatal care for a positive pregnancy experience. World Health Organization; 2016.
2. World Health Organization. Trends in maternal mortality: 1990-2015: estimates from WHO, UNICEF, UNFPA, World Bank Group and the United Nations Population Division: executive summary. World Health Organization; 2015.
3. Rurangirwa AA, Mogren I, Nyirazinyoye L, Ntaganira J, Krantz G. Determinants of poor utilization of antenatal care services among recently delivered women in Rwanda; a population based study. BMC pregnancy and childbirth. 2017;17(1):142.
4. Gunawardena N, Bishwajit G, Yaya S. Facility-Based maternal death in Western Africa: a systematic review. Frontiers in public health. 2018 Feb 26;6:48.
5. Ghana Statistical Service (GSS), Ghana Health Service (GHS), and ICF. 2017 Ghana Maternal Health Survey Research Policy Briefs. Accra, Ghana: GSS, GHS, and ICF. 2019
6. UNICEF. Antenatal care. 2018. Available from; https://data.unicef.org/topic/maternal-health/antenatal-care/ (accessed March 7, 2019).
7. WHO: Global Health Observatory data repository**:** Antenatal care coverage Data by country Available from; http://apps.who.int/gho/data/node.main.ANTENATALCARECOVERAGE4 (accessed 17 January 2020)
8. WHO: Global Health Observatory (GHO) data; Country specific: Available from; https://www.who.int/gho/countries/en/

9. Fagbamigbe AF, Idemudia ES. Barriers to antenatal care use in Nigeria: evidences from non-users and implications for maternal health programming. BMC pregnancy and childbirth. 2015 Dec 1;15(1):95.

10. Bustreo F, Say L, Koblinsky M, Pullum TW, Temmerman M, Pablos-Mendez A. Ending preventable maternal deaths: the time is now. Lancet, Glob Heal. 2013;1:e176–7.

11. Ashir GM, Doctor HV, Afenyadu GY. Performance based financing and uptake of maternal and child health services in yobe sate, northern Nigeria. Glob J Heal Sci. 2013;5:34–41.

12. Ghana Health Service, Family Health Division, Annual Report . 2016. https://www.ghanahealthservice.org/downloads/FHD_2016_ANNUAL_REPORT_Final_June%2019.2017%20nat%20final.pdf

13. Hijazi HH, Alyahya MS, Sindiani AM, Saqan RS, Okour AM. Determinants of antenatal care attendance among women residing in highly disadvantaged communities in northern Jordan: a cross-sectional study. Reproductive health. 2018 Dec;15(1):106.

14. World Health Organization. Antenatal care in developing countries: promises, achievements and missed opportunities. 2003. Available from; http://apps.who.int/iris/bitstream/handle/10665/42784/9241590947.pdf?sequence=1

15. Babitsch B, Gohl D, Von Lengerke T. Re-revisiting Andersen's Behavioral Model of Health Services Use: a systematic review of studies from 1998–2011. GMS Psycho-Social-Medicine. 2012;9.

16. Andersen RM. Revisiting the behavioural model and access to medical care: does it matter? J Health Soc Behav. 1995;3(6):1–10.

17. Andersen R, Newman JF. Societal and Individual Determinants of Medical Care Utilization in the United States. Milbank Q. 2005;83(4):1–28.

18. Ogunba BO, Abiodun OB. Knowledge and attitude of women and its influence on antenatal care attendance in Southwestern Nigeria. J Nutr Health Sci. 2017;4(2):207.

19. Yamane T. Statistics, An Introductory Analysis, 2nd Ed., 1967. New York: Harper and Row.

20. Ministry of Health. National Community-Based Health Planning and Services Policy. Ghana, Accra. 2006

21. Ghana Health Service (GHS). The Community-Based Health Planning and Services initiative: Concepts and plans for implementation. Accra, Ghana. 2002.

22. Ministry of Environment Science and Technology (MEST). Zoning guidelines and planning standards. 2011.

23. World Health Organization, United Nations Population Fund, United Nations International Children's Emergency Fund, The World Bank. Pregnancy, childbirth, postpartum and newborn care: a guide for essential practice (3rd edition). World Health Organization. Available from: http://www.who.int/maternal_child_adolescent/documents/924159084x/en/

24. Birmeta K, Dibaba Y, Woldeyohannes D. Determinants of maternal health care utilization in Holeta town, central Ethiopia. BMC Health Serv Res. 2013;13(1):256.

25. Ayalew TW, Nigatu AM. Focused antenatal care utilization and associated factors in Debre Tabor Town, northwest Ethiopia, 2017. BMC research notes. 2018 Dec 1;11(1):819.

26. Gitonga E. Determinants of focused antenatal care uptake among women in tharaka nithi county, Kenya. Adv Public Health. 2017;2017:4.
27. Sakeah E, Okawa S, Rexford Oduro A, Shibanuma A, Ansah E, Kikuchi K, Gyapong M, Owusu-Agyei S, Williams J, Debpuur C, Yeji F. Determinants of attending antenatal care at least four times in rural Ghana: analysis of a cross-sectional survey. Global health action. 2017;10(1):1291879.
28. Tuladhar H, Dhakal N. Impact of antenatal care on maternal and perinatal outcome: a study at Nepal Medical College Teaching Hospital. Nepal J Obstet Gynaecol. 2011;6(2):37–43.
29. Manyeh AK, Amu A, Williams J, Gyapong M. Factors associated with the timing of antenatal clinic attendance among first-time mothers in rural southern Ghana. BMC Pregnancy and Childbirth. 2020;20(1):47.
30. Nebeb GT, Salgedo WB, Alemayehu YK. Antenatal care utilization in Debre Tabor, North West Ethiopia. Gynecol Obstet. 2015;5:339. https ://doi.org/10.4172/2161-0932.10003 39.
31. Kalule-Sabiti I, Amoateng AY, Ngake M. The effect of socio-demographic factors on the utilization of maternal health care services in Uganda. Afr Popul Stud. 2014;28(1):515–25.
32. Tiruaynet K, Muchie KF. Determinants of utilization of antenatal care services in Benishangul Gumuz Region, Western Ethiopia: a study based on demographic and health survey. BMC pregnancy and childbirth. 2019;19(1):115.
33. Okedo-Alex IN, Akamike IC, Ezeanosike OB, Uneke CJ. Determinants of antenatal care utilisation in sub-Saharan Africa: a systematic review. BMJ open. 2019;9(10):e031890.

34. Ayamolowo SJ, Irinoye O, Oladoyin MA. Job satisfaction and work environment of primary health care nurses in Ekiti state, Nigeria: an exploratory study. *Internatinal Journal of Caring Sciences*. 2013;6:531–42.
35. Anarfi KJ. To Change or not to change: obstacles and resistance to sexual behavioural change among the youth in Ghana in the era of AIDS. Res Rev. 2003; 19:27–45.
36. Nketiah-Amponsah E, Senadza B, Arthur E. Determinants of utilization of antenatal care services in developing countries: recent evidence from Ghana. Afr J Econ Manag Stud. 2013;4:58–73.
37. Amoakoh-Coleman M, Ansah EK, Agyepong IA, Grobbee DE, Kayode GA, Klipstein-Grobusch K. Predictors of skilled attendance at delivery among antenatal clinic attendants in Ghana: a cross-sectional study of population data. BMJ open. 2015;5(5):e007810. e007810.
38. Arthur E. Wealth and antenatal care use: implications for maternal health care utilisation in Ghana. Health Econ Rev. 2012;2:14.
39. Ayanore MA, Pavlova M, & Groot, W. Focused maternity care in Ghana: results of a cluster analysis. BMC Health Serv Res 16, 395 (2016). https://doi.org/10.1186/s12913-016-1654-5.
40. Afaya A, Dzomeku VM, Baku EA, Afaya RA, Ofori M, Agyeibi S, Boateng F, Gamor RO, Gyasi-Kwofie E, Nyaledzigbor PP. Women's experiences of midwifery care immediately before and after caesarean section deliveries at a public Hospital in the Western Region of Ghana. BMC Pregnancy and Childbirth. 2020;20(1):1-9.
41. Duku S, Alhassan RK, Nketiah-Amponsa E. Barriers to Uptake and Renewal of Health Insurance Membership: Experiences from Ghana's National Health Insurance Scheme (NHIS). 2016

42. Kawungezi PC, AkiiBua D, Aleni C, Chitayi M, Niwaha A, Kazibwe A, Sunya E, Mumbere EW, Mutesi C, Tukei C, Kasangaki A. Attendance and utilization of antenatal care (ANC) services: multi-center study in upcountry areas of Uganda. Open journal of preventive medicine. 2015;5(3):132.
43. Ali SA, Dero AA, Ali SA. Factors affecting the utilization of antenatal care among pregnant women: a literature review. J Preg Neonatal Med 2018; 2 (2).
44. Fagbamigbe AF, Akanbiemu FA, Adebowale AS, Manuwa-Olumide A, Korter G. Practice, knowledge and perceptions of antenatal care services among pregnant women and nursing mothers in Southwest Nigeria. International Journal of Maternal and Child Health. 2013;1(1):7-16.
45. Gong E, Dula J, Alberto C, de Albuquerque A, Steenland M, Fernandes Q, Cuco RM, Sequeira S, Chicumbe S, Gudo ES, McConnell M. Client experiences with antenatal care waiting times in southern Mozambique. BMC health services research. 2019;19(1):538.
46. Gupta RK, Shora TN, Verma AK, Jan R. Knowledge regarding antenatal care services, its utilization, and delivery practices in mothers (aged 15-49 years) in a rural area of North India. Tropical Journal of Medical Research. 2015;18(2):89.

PONE-D-20-05929
Women's knowledge and its associated factors regarding optimum utilisation of antenatal care in rural Ghana: a cross-sectional study

Agani Afaya (MSc), Thomas B. Azongo (PhD), Veronica Millicent Dzomeku (PhD), Hyeonkyeong Lee (PhD), Richard Adongo Afaya (MPhil), Solomon Mohammed Salia (MSc), Peter Adatara (PhD), Robert Kaba Alhassan (PhD), [6]Martin Amogre Ayanore (PhD), Abigail Kusi-Amponsah Diji, Confidence Alorse Atakro (MN), David Adadem (BN),Emmanuel Opoku Asiedu (BN), Paul Amuna (MB ChB, PhD)

Dear Editor,

We would like to thank you sincerely for the insightful reviewer comments on our manuscript and for the opportunity to resubmit the manuscript for another round of review. We find the comments very useful and have responded to them to the best of our knowledge. We acknowledge that the comments have no doubt helped improve the quality of our manuscript.

We herein provide further details by showing a point-by-point feedback on how each of the comments received were addressed. For easy identification, the reviewers' comments have been repeated while Authors' responses appear in **BOLD** text.

Amendment of Authorship

I would like to amend authorship to include Dr. Abigail Kusi-Amponsah Diji who contributed to conceiving and designing the study and contributed enormously to this paper. Her name has been included in the manuscript and highlighted in yellow ink.

I apologise as this omission occurred during the initial submission process.

Thank you.

Review comments

Reviewer #1
Review comments

1. Line numbering would have made it easy to referencing.

Authors response

The Manuscript was formatted according to the Journal specifications. However, in view of the comment, line numbering has been included in the revised manuscript for easy referencing.

Review comment

The statement "The sub-district has semi-urban area but predominantly rural" under study design section should be put under study settings.

Authors response

Changes have been affected as suggested and now appears in the revised manuscript in page 8, lines 154-155.

Review comment

3. The variable "Number of Children" can't be dichotomy when the categories are more than two. "The number of children was dichotomised into 1-2, 3-4 and 5+ children"

Authors response

The sentence has been revised: dichotomized has been changed to categorized. This is can be found in page 10, line 189 of the revised manuscript.

Review comments

4. How was the distance to health facility measured? Distance from where to health facility? What distance is categorized as "far" and what is "near"?

Authors response

For CHPS, the distance was between a radius of 5 km (GHS, 2002; MoH, 2016) and for Health Centres between 8 and 16 km radius (MEST, 2011). Women who trekked more than 5km and also 16km where considered far and those within the recommended limit were considered short. See page 10 lines 191-193.

[Ministry of Health, (2016). National Community-Based Health Planning and Services Policy. Ghana, Accra.

Ghana Health Service (GHS), (2002). The Community-Based Health Planning and Services initiative: Concepts and plans for implementation. Accra, Ghana.

Ministry of Environment Science and Technology (MEST), 2011. Zoning guidelines and planning standards.

Review comments

5. "Those who sought ANC services less than four times were classified as having poor ANC service utilisation (inadequate) and those who had ANC visits of 4 or more were considered as having good (adequate) ANC service utilisation." This information has been captured under the section on outcome variables already. Please delete it.

Authors response
Repetition deleted under data analysis section see page 10, lines…

Review comment
6. Revise this statement: "Bivariate logistic regression analysis, computing odds ratio, was used to determine the strength of the association…" Because Logistic regression will report odds ratio. Bivariate logistic regression analysis reports more than strength of association. What about direction of the association?
Authors response
In page , lines this has been revised and now reads as; "Ordinary least square logistic regression analysis was applied where odds ratios were computed to determine how the independent variables is associated with the dependent variables (ANC service utilisation and knowledge of ANC)"

Review comments
7. The statement "ANC service utilisation was coded as 0 for poor utilisation (<4 ANC visits) and 1 for good untilisation (4+ ANC visits)" has been repeated under the outcome variables and twice under data analysis sections. Please check and delete where appropriate.
Authors response
The repetition highlighted by the reviewer under data analysis have been deleted in the revised submission.

Review comments
8. Please provide the Ethics approval number.
Authors response
This has been provided in page line and now reads as "Ethics approval for the study was obtained from the University of Health and Allied Sciences Research Ethical Committee (UHAS-REC A. 10[27] 17-18)".

Review comments
9. What is purpose of running a chi-square test and crude logistic regression as reported in table 2?
Authors response
Chi-square results has been removed and replaced with tables only highlighting analysis of logistic regression (for adjusted and unadjusted)

Review comments
10. Table 2 is titled "Regression analysis results for ANC attendance" but reported Chi-square results in addition. I suggest the authors present the results of the logistics regression only in tables 2 and 3

Authors response
As suggested by the reviewer, authors have re-presented the results of the logistics regression only in tables 2 and 3.

Review comments
11. How were confounding effects accounted for in establishing the association between the dependent and independent variables? Think the authors have to explore association in presence of other explanatory variables. This part of the analysis is not clear.
Authors response
National health insurance was controlled for in the adjusted model. When NHIS was controlled for in the adjusted model there was a significant relationship with optimum ANC and these explanatory variables (Age, marital status, educational level, religion, and distance to health facility). There for NHIS was found to have confounder effect when included in the regression model.

Review comments
12. How was the Family type defined? how was the question asked?
Authors response
Nuclear family was termed as a *family* group consisting of two parents and their children (one or more). Extended family: consisting of parents like father, mother, and their children, aunts, uncles, grandparents, and cousins, all living in the same household.
Do you live with your husband and children alone or with your other extended family (grandfather etc)

Review comments
13. In Figure 6, how is "Check-up" different from "Normal routine during pregnancy"? How were those two questions asked?
Authors response
Check-up: the main reason behind they attending ANC was to check their health status and that of their unborn baby. "Normal routine during pregnancy" mothers that were categorized here where those who mainly said their reason for attending ANC was because it is the routine thing to do during pregnancy and has been asked to come on this date by nurses.

Review comments
14. The statistical association cannot be strongly established with only crude results without accounting for explanatory variables. I suggest a revision of the statistical analysis to inform the conclusion of the study.
Authors response
Reviewer concerns have been addressed in table 2 and 3

Reviewer # 2

Review comments
The study seems to assess optimal utilization (four or more visits) of ANC among utilizers of ANC and not utilization of ANC as suggested by the topic.
Authors response
Taking the reviewer suggestion into consideration, authors have revised the title as found in the revised manuscript submitted. See page 1 lines 2-3

Review comments
Also, the authors should be adjusted the title to reflect that they only assessed socio-demographic factors.
Authors response
While acknowledging that this is a fair comment from reviewers, other factors outside individual socio-demographic factors were assessed i.e community and individual challenges affecting antenatal utilization; facility related barriers to ANC attendance; reasons for ANC Attendance etc. Hence, adjusting the title to reflect socio-demographic factors has a potential to limit the scope of factors considered in this study.

Given the title change in this revised submission, authors think we have adequately addressed any concerns regarding the suitability of the title and that the title now reflects the aim/objectives of the study.

Introduction:
review comment

Some of the text require references.
Authors response
References have been provided in relevant areas in the introduction section.

Review comment
The justification is not compelling enough. According to you, ANC attendance is high but utilization for delivery is low. Isn't this a more interesting topic than exploring factors for ANC attendance? The authors need to better justify the investigation.
Authors response
This has been addressed in page 4 and 5 lines 89-104. The highlighted portions provide further clarity on areas these have been addressed.

Review comments
Conceptual framework:

Authors had a beautiful conceptual framework but limited the investigation to a few socio-demographic correlates of optimal ANC attendance. The reason for this is not clear.
The authors seem to suggest that attendance of four or more ANC visits is synonymous with utilization rather than optimal utilization.

Authors response
We adapted the behavioural model framework of Andersen for use of health services to identify the factors that potentially facilitate or impede minimum number of antenatal health services seeking behavior at individuals and community levels.

In considering the review suggestion, authors have made changes and have maintained optimal utilisation instead of utilisation in this revised submission of the manuscript.

Review comments
Could the authors briefly explain the reasons for its (Anderson Behavioral Health care model) wide applicability? Why choose this model for the inquiry? Why not any other model?
Authors response
The frame work is widely used because the three factors identified by Andersons model to facilitate or impede healthcare access cuts across almost every health system across the globe including the developed and developing countries. And several studies have used the model to identify facilitating and impeding factors to healthcare access.

The framework predicts that a series of factors predisposing, enabling and need factors influence the utilization of health services by people. According to the model, predisposing factors are demographics and social structures. Enabling factors facilitates individuals to use services for example, availability of resources such as income, access to free services, availability and access to the service. Need factors motivates service use. We chose this model because we sought to determine factors that impede or facilitate ANC services utilization in health facilities. And Anderson behavioral model best suit our current study objective.

Methods:
Review comments
Study design:
I am not convinced that the method is adequate to answer the research question.
Assessing factors related to utilization of ANC services would be better answered by comparing utilizers and non-utilizers. Rather the design addresses correlates of optimum utilization (four or more) among utilizers. The authors need to clarify this.
Authors response

Reviewer suggestion and comments was helpful in making revisions to the design section of the revised manuscript. This study truly addresses correlates of optimum utilization among users and this has reflected in the revised manuscript under the study design section. See page 8 lines 137-140.

Outcome variable:

Review comments

How many outcome variables did this study have? The first sentence suggests more than one but itemizes just one. Kindly correct.

Authors response

The outcome variables were optimum utilisation and Knowledge of ANC. This has been addressed under outcome variables. See page 9 lines 172-173.

Review comments

The authors should use “good” or “bad” knowledge for the sake of consistency and not knowledgeable or not.

Authors response

Authors acknowledge this as a fair suggestion. However, to ensure consistency in the revised manuscript, authors have used good and poor knowledge.

Review comments

The characterization of participants seems unclear. A part of the manuscript suggests that 50% was used as cut-off, while another part suggests that the participants’ mean score was used as cut-off. Kindly clarify and justify.

Authors response

Authors acknowledge the inconsistent reporting in the methods section with regard to the cut-off point in knowledge assessment. Author has corrected this inconsistency by deleting the 50% cut-off point.

Review comments

Validity and reliability of the study instrument:

How was construct validity assured?

Authors response

construct validity pertains to a specific use of a scale and can often be context or population dependent. The current study instrument was contextually structured to identify factors that facilitate or impede optimal utilization of ANC services within the study population.

Multi-collinearity diagnostics were done and found that the Variance Inflation Factors (VIFs) were all below the 5-10 rule of thumb range, suggesting there is no collinearity among the independent variables to be fitted in the regression model! Effects of multi-collinearity was therefore ruled out and did not have any effect on the validity of the study outcomes.

Review comments
What is the level of expertise of these researchers and clinicians? It may be helpful to state their qualification and give further background about their experience.
Authors response
Thomas B. Azongo (PhD) is a snr lecturer and a public health expert; Veronica Millicent Dzomeku (PhD) is a midwife and a Snr Lecturer, Hyeonkyeong Lee (PhD) is a professor in community health nursing; Peter Adatara (PhD) is a snr lecturer and his specialty area is in maternal and child health; Robert Kaba Alhassan (PhD), Snr research fellow; Martin Amogre Ayanore (PhD) is a Health Economist and a Public health expert, Paul Amuna (MB ChB, PhD) is a professor and his area of specialty is maternal and child health
Clinicians: They both have degree in midwifery and have been practicing midwifery for over 10 years

Review comments
Questions 5, 6, 8, 9, 10 do not actually test what the women know?
Authors response
These questions were added to assess mothers knowledge on the other activities carried during ANC: Mothers that utilize ANC are taught these and they are expected to have basic knowledge in relation to the education or things taught during ANC attendance: for example during ANC, mothers are informed of the recommended ANC visits, so we expected mothers to know these. Additionally, mothers who utilized ANC are educated on the recommended place for safe delivery and the necessary items required for delivery. The items for assessing the mother's knowledge of ANC was developed in context with the study population

Review comments
Ethical consideration:
Could you include the reference number for the IRB approval?
Authors response
Ethics approval for the study was obtained from the University of Health and Allied Sciences Research Ethical Committee (UHAS-REC A. 10[27] 17-18).

Results:
Review comments
Authors did not report results of bivariate analysis despite proposing to do them in the methods section.
Authors response
The results section has been updated and review concerns above addressed. See table 2 and 3 on pages 16 &17 lines 284-285

Review comment
There is a need to report the salient findings even if all has been presented on table 2.
Authors response
Authors acknowledge this and have done the needful by focusing on only the salient findings. See page 15 lines 278-283.

Review comment
What is the mean age of the participants?
Authors response
Age of participants were categorized during data collection therefore the mean age could not be calculated.

Review comments
The title of table 2 need to be updated to bivariate and regression analysis. The authors need to make some comments about results of bivariate analysis in prose.
Authors response
Table 2 and 3 have been updated.

Review comments
Figure 3 is better presented as a pie chart.
Authors response
Figure 3 has been changed to a pie chart. Refer to page 18.

Review comment
On table 3 and age factor, if the reference group is the first group, then the Odds ratio need to be reviewed.
Authors response
This critical suggestion was reviewed and significant changes made to the odds ratios. Table 3 provide further details on the revised submission after addressing this concerns. See page 20.

Review comments
For figure 6, kindly remove the bars with zero responses.
Authors response
Changes has been made as suggested. Refer to page 14

Discussion:

Review comment

“The present finding of 69% is below the national coverage of at least four ANC visits of 75.9%”. Is there something about the study population that could account for this disparity?

Authors response

This has been addressed in page 25 line 348-351 and now reads as;

This difference may be due to the lack of awareness of optimal ANC visits. Also, the differences could have been caused by other potential inhibiting factors such as distance to health facilities and socio-economic factors.

Review comments

“Our study finding is consistent with a study conducted in the southwest of Nigeria [39] but disagrees with a study conducted in Mozambique where the researchers found women’s knowledge of ANC services not to have significant importance in their utilisation of ANC services [40]. It is established that women with good knowledge of ANC services have a better understanding and acceptance of the services provided during ANC [41], and this knowledge will, therefore, be a catalyst for the utilising ANC services during pregnancy.” It seems more plausible that utilization of ANC services would result in better knowledge of ANC not the other way round as suggested by the authors. Anyway, the study is cross-sectional, hence the use of the word determinant is better replaced with association.

Authors response

We totally agree with assertion (It seems more plausible that utilisation of ANC services would result in better knowledge of ANC). In the discussion section, this emphasizes has been made in relation to the findings of the study.

Limitations:

Review comments

I can immediately think of some other limitations of this study e.g. cross-sectional nature, therefore, just associations can be measured, bias due to self-report, non-exploration of many other factors, etc. Kindly develop this section further.

Authors response

A section on limitations has been added and some potential limitation of the study acknowledged. See page 29 lines 448-458

Review comments

Other comments”

I have made some further suggestions on the text of manuscript

Authors response

Review concerns on inconsistencies in the manuscript have been addressed. Authors read thoroughly through the revised manuscript to identify these inconsistencies and sentences that were not clear and corrections effected.

Reviewer #3:

Manuscript Title

Women's knowledge of and factors associated with the utilization of antenatal care in rural Ghana: analysis of a community-based cross-sectional study

Review

General Comments

Organization of the various sections need clarity and simplification of sentences. all sections must conform to the STROBE checklist for presenting prevalence studies.

Authors response

We acknowledge and used the STROBE checklist to guide the study (methods)

Specific Comments

Review comments

• Study Setting- is this a community based or facility-based study? The title says community but the methods state facility based.

Authors response

The title has been revised to reflect these concerns. See page 1

Review comment

• Questionnaire administration- were the questionnaires self-administered or was administered by a research assistant or both?

Authors response

The questionnaires were administered by only the four research assistants trained for data collection

Review comment

• Table 2 needs to be simplified, Summarized; the detailed version can be attached as an appendix.

Authors response

Authors acknowledge and have addressed it. Authors reported on only salient findings of the study but not every variable see table 2 on pages 16 & 17 lines 284-285.

Review comment

• "Instead, the current study found women with low socioeconomic status more likely to utilize ANC services at least four times due to their enrolment into the national health insurance scheme" where was this result presented?

Authors response

This was a general assumption for the study participants. This have been deleted because it lacked evidence from the current study.

Review comments

Are there any reasons why Bole had a lower than National ANC4+, What is the regional average?

Authors response

The difference may be due to the lack of awareness of optimal ANC visits. Also, the differences could have been caused by other potential inhibiting factors such as distance to health facilities and socio-economic factors. Refer to page 25 line 348-351.
